# Report from a Workshop on Accelerating the Development of Treatments for Inherited Retinal Dystrophies Associated with Mutations in the *RDH12* Gene

**DOI:** 10.1167/tvst.9.8.30

**Published:** 2020-07-17

**Authors:** Francesca Sofia, Silvia Cerolini, Todd Durham

**Affiliations:** 1 Science Compass, Milan, Italy; 2 Through Vicky's Eyes, London, UK; 3 Foundation Fighting Blindness, Columbia, MD, USA

**Keywords:** nonclinical models, gene therapy, clinical trial, endpoints, natural history

## Abstract

The Foundation Fighting Blindness, *RDH12* family organizations, and the *RDH12* Fund for Sight convened a jointly organized workshop in Columbia, Maryland, on November 19, 2019. The purpose of the workshop was to share perspectives on what is known about the *RDH12*-associated retinal dystrophies (RDs) and discuss the advancement of therapies, primarily gene therapy, for people with mutations in the *RDH12* gene which cause Leber congenital amaurosis 13 (LCA13). The workshop began with presentations on the *RDH12* landscape, patient perspectives, the use of statistical modeling for clinical trial design, and the Foundation's My Retina Tracker Registry. An afternoon roundtable discussion focused on four key areas essential to advance research toward gene therapy clinical trials: trial design, dose projection from nonclinical to clinical studies, natural history, and regulatory considerations. In their comments, the 27 participants from academic centers, affected families, biotechnology and pharmaceutical companies, and the regulatory community highlighted a number of research priorities, including the following: systematic inventory of retrospective natural history studies and planning for a multicenter prospective study, development of large animal models, and evaluation of novel tests of functional vision in young children. Despite these research opportunities, the workshop participants agreed that the field could be ready now for a clinical trial aimed initially at testing the safety and, possibly, efficacy of *RDH12* gene therapy. Advancements in this field are being fostered by the emergence of an innovative multi-stakeholder research endeavor that relies on the effective engagement of the patients.

**Translational Relevance:**

This initiative serves as a model for how affected individuals and their families can be research partners on the path to treatments and cures.

## Introduction

The Foundation Fighting Blindness and the *RDH12* family organizations convened a jointly organized workshop in Columbia, Maryland, on November 19, 2019. The overall goal was to bring together a group of patient representatives, clinicians, and academic and industry researchers to discuss clinical trial readiness for treatments of *RDH12*-associated inherited retinal disorders (IRDs). Specific objectives included the following: share perspectives and insights on what is known about the *RDH12*-associated IRDs; identify specific challenges to the successful development of treatments for *RDH12*-associated IRDs; explore key trial elements, including trial design and regulatory implications; and generate a prioritized list of research questions and an opportunity roadmap with the ultimate goal of improving the probability of successful treatment development.

The workshop was organized by Todd Durham, PhD, Vice President of Clinical & Outcomes Research, Foundation Fighting Blindness; Silvia Cerolini, a patient family representative; and Francesca Sofia, PhD, Consultant, Science Compass. The format of the workshop was a morning of presentations followed by an afternoon roundtable discussion. The remainder of this paper highlights the key points of the workshop, which have been grouped for readability by topical area rather than in chronological order.

### Current Landscape of *RDH12* Retinal Dystrophies

Francesca Sofia briefly presented the highlights of her full landscape report of *RDH12* RDs (see Supplementary Material), from the current understanding of genetics, pathophysiology, clinical manifestations, therapeutic approaches, and research precedents from other clinical trials, including the development of Luxturna for *RPE65*-associated RD.

As she summarized in her presentation, Leber congenital amaurosis (LCA) is a molecularly and clinically heterogeneous group of inherited retinal disorders characterized by vision loss, involuntary eye movements (nystagmus), and severe retinal dysfunction.^1^^–^^3^ LCA, which accounts for 5% of all IRDs, is the leading cause of inherited childhood blindness in the first decade of life. It has high genetic heterogeneity, with 25 causative genes identified to date. Among those, *RDH12*, which encodes a photoreceptor-specific retinal dehydrogenase, causes LCA type 13 (LCA13).

The pathogenetic mechanism associated with *RDH12* mutations is not completely understood; however, it correlates with the loss of RDH12 enzymatic activity and consequent accumulation of toxic compounds in photoreceptor cells. Over 100 different mutations in *RDH12* have been found to cause LCA13, which manifests with visual field constriction, loss of visual acuity, and night blindness in the first years of life and inescapably progresses to legal blindness in early adulthood.

At present, there is no treatment for LCA13, but the field of *RDH12* research is quite active, as evidenced by the numerous publications, involvement of the affected families in research, and participation of leading researchers in the workshop itself. Basic and preclinical research addressing *RDH12* disease mechanisms and/or testing potential therapeutic interventions is hampered by the lack of reliable in vivo and in vitro models. Furthermore, due to the rarity of LCA13, clinical knowledge of the disease is confined to a few natural history studies conducted by individual clinical and research institutions. Nevertheless, interviews with experts in the field (see Supplementary Material) revealed that there are several opportunities in the therapeutic pipeline ranging from gene therapy—the most promising and advanced one on the path to cure—to other gene-agnostic approaches. Among those, neuroprotective strategies involving either gene therapy or neuroprotective compounds may slow down disease progression and provide a longer time window for treatment. Efforts are also being made in the field of optogenetics that are aimed at vision restoration by precisely exciting the neural apparatus. In addition, cell replacement therapies are being explored in IRDs and could, therefore, be applied to *RDH12* RD, although several concerns have yet to be addressed. Finally, gene editing and antisense oligonucleotides may provide additional means to tackle the dominant forms of the disease.

In order to make the most of this promising pipeline of therapies, some major gaps will have to be filled; for example, mouse models developed so far do not recapitulate the human phenotype and better animal models would be needed. In addition to this nonclinical testing ground for new therapies, in vitro preclinical testing using induced pluripotent stem cells (IPSCs) and organoids holds great promise; however, these models are relatively early in their development and require further refinement. Similarly, clinical knowledge of the disease is limited to a few natural history studies conducted by individual centers. For this reason, two steps would be beneficial: an inventory of completed clinical studies and the available data and, if warranted, a multi-center longitudinal observational study.

Any clinical study, whether observational or an experimental clinical trial, may benefit from the precedents of earlier LCA clinical trials. These studies shared several efficacy outcome measures including the following: multi-luminance mobility test (MLMT), full-field light sensitivity threshold (FST) testing, best-corrected visual acuity, pupillometry (pupillary light reflex), optical coherence tomography (OCT), and fundus autofluorescence. Safety assessments included adverse events, ophthalmic examination, physical examination, and laboratory testing. A few patient-reported outcomes have been used in trials thus far, although none has been developed specifically for the largely pediatric LCA population.

### *RDH12* Patient Family Perspectives

The unique perspectives of *RDH12* patients were represented by the *RDH12* family group that includes over 100 families worldwide affected by *RDH12*-associated LCA/retinitis pigmentosa. These families share a common goal to accelerate research and support and develop the community of patients. Consistent with this objective, family representatives attending the workshop described their journey with *RDH12* retinal dystrophy after Silvia Cerolini presented results of a survey that was distributed prior to the workshop. Among the 61 patients responding to the survey, the most commonly reported symptoms were lack of or limited peripheral vision (78%), night blindness (73%), and poor visual acuity (69%). The most bothersome disease impacts included reduced independence, inability to drive, progression to total blindness, fear of the unknown, and feeling different and socially isolated. Most of the responding patients reported deterioration in their vision since their diagnosis; changes that have led to loss of the ability to read; reliance on others, canes, or guide dogs; struggles to recognize faces; and an overall negative impact on mental health. The patients expressed the hope that further deterioration in vision would be halted by treatments studied in future clinical trials. And if trials were to be conducted in the future, nearly all patients expressed an interest in being informed about them, despite having concerns about the treatments themselves, including the risk of further deterioration, safety, and postoperative complications and side effects.

### Use of Disease Progression Models to Inform Clinical Trial Design

Barbara Wendelberger presented statistical innovation in the design of clinical trials for rare diseases and showed how disease progression for various outcome measures can be modeled as a function of disease onset and other patient characteristics. Experience from these models in rare diseases with long-term progression has shown that the most useful clinical trial endpoints are those with low variability and a high rate of decline. Careful evaluation of outcomes and endpoints derived from them requires natural history studies with multiple outcome measures reported systematically. When the disease progression models have been developed, they can be used in virtual patient simulation to optimize elements of the clinical trial design, including the length of follow-up, patient population, primary endpoints, sample size, and statistical operating characteristics.

### My Retina Tracker Registry as a Recruitment Tool

Brian Mansfield presented My Retina Tracker Registry (MRTR), a patient-centric registry that collects data from patients and clinicians about several thousands of people with inherited retinal dystrophies and eye disorders. To date, approximately 14,300 useful online profiles have been recorded. These registrations accelerated to about 400 new entries per month after 2017 when the Foundation launched a genetic testing program. Among all MRTR members with genetic test results, 49 carry at least one *RDH12* allele, 47 were independent reports, and 11 have *RDH12* as a secondary variant. Overall, 36 MRTR members have *RDH12* as a primary genetic finding and are affected with an IRD.

### Commentary on the Presentations

During the discussions that followed the morning's presentations, several noteworthy comments and suggestions were raised by the participants:

• Debra Thompson stated that her team showed that gene therapy protects the retinas of *RDH12* knockout mice exposed to damaging levels of light. She also shared the news that MeiraGTX (New York, NY) had licensed *RDH12* gene therapy from her institution.
• Bart LeRoy suggested that the Foundation should bring MRTR to the attention of the European Reference Network–Eye Diseases.
• James Tobin emphasized the need to develop a molecular description of the disease (i.e., how different *RDH12* mutations correlate with the disease).
• Wiley Chambers advised not to commit time and resources collecting quality-of-life parameters, as there are other measures that would be more valuable to the U.S. Food and Drug Administration (FDA) during a possible approval process.

## Roundtable Discussion

### Natural History

Bart LeRoy stated that many people with *RDH12* mutations retain a significant amount of vision well into adulthood; therefore, he cautioned against labeling the associated disease as LCA. He and other meeting participants agreed that many adults with *RDH12* mutations are genetically undiagnosed and identifying them would help populate natural history studies and clinical trials. He also emphasized that patients with *RDH12* mutations exhibit areas of the retina that are much better preserved and mentioned the so-called patchy preservation of the peripapillary and peripheral retina as a predictive feature of *RDH12* retinal dystrophy, a sign that should immediately call for targeted gene testing.

Wiley Chambers pointed out that natural history is normally very valuable but seems to be less valuable in *RDH12*, as there are many ophthalmologists—such as those who were sitting around the table—who are monitoring *RDH12* natural history. Therefore, he suggested that it is not worth investing in and waiting for natural history before moving on with an interventional trial. Abigail Fahim added that a multicenter prospective observational study for *RDH12* would be feasible and that the more centers involved, the better the study.

Rick Ferris highlighted the value of prospective natural history studies by describing the example of the Foundation's Usher syndrome study, RUSH2A. In this study, data are collected prospectively and specific outcome variables are being measured longitudinally with the idea of assessing not only the variability of the measure but also its rate of progression. This study is showing that longitudinal observations can be very informative but difficult due to high variability across a small number of patients who are genetically different and in different stages of the disease. He added that it is likely that *RDH12* would benefit from the same systematic collection of data in which imaging can be used. However, he pointed out that investigators can identify outcome measures from retrospective studies and, at the same time, use data from a control group to learn about natural history. In this context, even if the treatment does not work, a trial would reveal insights for a future trial because it is a source of data on disease progression.

The group discussed the merits of prospective versus retrospective natural history studies. Barbara Wendelberger noted that not having a prospective natural history study is not an impediment to moving forward. Particularly in diseases whose mechanisms and natural history are poorly understood, any available information to either rule out or use what is assumed to be a good outcome measure can serve to improve their statistical model of disease progression. Everyone agreed that prospective studies are richer and better in terms of quality of data and standardization, but these studies are very expensive and demanding. By contrast, retrospective studies have lower quality because of the heterogeneity of collection methods. This might be overcome if imaging methods were standardized and a model for progression rate could be developed using retrospective data, although it would be equally challenging to collect these data. The remaining questions were as follows: What data are currently available on these measures? Is there a way to understand disease progression from the current data? How do we consolidate those data? And, do we have natural history study data that allow us to move ahead with clinical trial design?

Abigail Fahim noted that data are available from retrospective cohorts; however, they are scattered. Every patient has visual acuity, a lot have OCT, not everyone does the visual field, and they undergo imaging but not every year. This is because when doctors see the patients they do not think in terms of natural history; however, data are available, and there are data centers that might be able to handle data coming from different sources and in different formats. So, knowing what would be acceptable by the regulators is paramount to deciding what to collect and consolidate as a first step toward understanding the natural history of the disease.

In the end, it was agreed that there are two paths to follow: One is to move on with a clinical trial making use of data from existing natural history studies. The other one would be to enable a longitudinal prospective study seeking the involvement of industry or another cost-sharing arrangement.

### Clinical Trial Design

Wiley Chambers explained that both functional and anatomical endpoints are important. Referring to previous experience in LCA, he stated that neither FST nor pupillometry would be acceptable as primary efficacy outcomes. However, a measure that shows how and if a treatment can prevent the loss of photoreceptors would provide an ideal anatomic endpoint that would logically translate into a functional outcome. On the other hand, considerable variability affects functional measures where a function is measured against a threshold that cannot always be correlated with the structural finding. Therefore, to the extent that there are clear anatomical measures, those should be employed in any *RDH12* gene therapy trial.

Bart LeRoy stated that the objectively assessed MLMT developed by Spark Therapeutics (Philadelphia, PA) for the Luxturna program, full-field stimulus testing, and detailed imaging of typically preserved areas would provide the outcome measures that are needed for an *RDH12* trial. FST and MLMT may be able to show results much faster than the anatomical preservation seen with OCT and other imaging modalities.

Katherine Uyhazi agreed that FST is a useful outcome measure. She added that her research institution also uses pupillometry because it is an objective measure of visual function that correlates very well with FST. Pupillometry is more useful than FST in young children because it does not require the cooperation of the patient.

Adam Dubis pointed out there were many virtues of pooling data. For one, a larger dataset would lead to better conclusions because poor-quality observations could be de-emphasized or removed. Additionally, larger data would open up the possibility of machine learning-based prediction modeling either alone or as a transfer learning exercise from other IRD datasets. The biggest challenge is accumulating enough data in one place. Other requirements include having graders trained in identifying where the boundary edges are, studying the data, and standardizing the methods. He also pointed out that data from at least 100 patients would be needed as long as multiple, evenly spaced datapoints were available. The sensitivity and specificity of this approach depend on how precisely the data going in are curated for type of data and duration between visits. Ultimately, the more data available, the better the models can be made.

Based on her experience with the study of Luxturna for *RPE65*-associated RD, Jean Bennett reported that Spark Therapeutics had several exploratory endpoints, which may be a useful approach. Spark thought that the electroretinograms were going to improve, but in fact they did not. However, a few exploratory endpoints, including pupillometry and light sensitivity, did show an improvement. So, the data from the phase I study were informative in terms of going forward with primary endpoints to demonstrate efficacy in phase III.

### Dose Projection

Wiley Chambers stated that from the FDA perspective, animal (i.e., nonclinical) testing would be needed in at least two species to show that the test product can be given to an animal without causing harm. These nonclinical toxicology studies need not be in a specific disease model (e.g., a large/non-human primate animal model).

With regard to doses, Debra Thompson stated that her laboratory's research in mice was done using the same doses that were used for other drugs or other gene therapies, and these proved to be primarily safe in the *RDH12* model. From this experience, she concluded that doses that have been used in mice could likely be proportionally given to patients.

The overall conclusion from the group was that 6-month nonclinical toxicology studies conducted in at least two species would be sufficient before going on to an initial human safety study.

### Regulatory Considerations

In addition to his earlier comments about endpoints and clinical trial design, Wiley Chambers raised a challenge of developing a gene therapy whose greatest benefit may be in children. He pointed out that rules within the United States would not allow children to be tested in a trial until there is some prospect for a direct benefit. Also, due to the lack of a good animal model showing success of the approach, adults ages 18 and older would be tested first in phase I. This initial study would not require many adults nor a definitive endpoint, but surely the trial would have to be done with people who could give informed consent. The obvious downside of this initial study is that adults are too far along in the disease; therefore, the results would not necessarily provide information about the product's true potential in children.

He further explained that the phase I study would require a cohort of three patients treated with the lowest dose to identify any safety problems before escalating to higher doses. If the data support overall safety at the lowest dose, the study could proceed to test a higher dose in three more patients and check again for safety problems. The study may include a third highest dose. From the first patients, one can usually assess whether or not adverse effects arise. From there, additional study patients can be randomized to the selected dose groups. Within the phase I studies, it would be necessary to demonstrate there was a prospect of benefit for children. The benefit could be restoration of some measure of vision or, usually, not getting worse.

Debra Thompson asked the group whether evidence of safety in adults, coupled with the nonclinical data showing a decrease in light damage, would be predictive that children might benefit from therapy. There was not a clear answer to this question, other than agreement that results from humans would be preferable to nonclinical data.

### Current State of Preclinical Evidence of Activity

Concerning preclinical in vivo evidence, Debra Thompson stated that there were two main findings supporting activity in mice. One is replacement of the enzyme in the retina from an in vivo injection that can be measured ex vivo by high-pressure liquid chromatography analysis. The other is protection against light damage susceptibility; that is, treated *RDH12* mice experience less photoreceptor cell death in the injected areas. However, evidence of this benefit for functional measures has not yet been tested, and the data to date support the role of RDH in photoreceptor death. It was clearly seen that knockout mice had much lower RDH activity than control mice; after gene therapy, the levels went up. The amount of activity present in injected mice per area was about twice the level of the wild-type enzyme. Overall, these data show that transduction and expression of the recombinant protein are very good. Of note, although *RDH12* mice may provide enough evidence in support of a gene therapy trial, other preclinical models in which retinal degeneration occurs would be needed for testing different therapies such as those for preventing damage from oxidation or combinatorial therapies. Also, this is likely where the field will eventually end up in order to help a lot more people affected by these diseases.

Jean Bennett confirmed that her group is developing a model using iPSCs, although these cells require a very long time to differentiate into patient-specific photoreceptors. Unfortunately, these cells do not become fully functional photoreceptors, likely because they are not attached to the retinal pigment epithelium. However, immature photoreceptors generated by the iPSCs may be useful in providing some preliminary data even though the system must be developed further.

### Priorities for Future Research

The group then brainstormed to develop a list of priority research areas and next steps to advance *RDH12* gene therapies. Overall, three main areas of interest were identified as being feasibly actionable:

*Gene therapy clinical trial*

1. Proceed with the gene therapy trial, because there is limited additional preclinical information that is going to increase confidence in a clinical trial at this point.
2. Develop a functional vision test that is appropriate for children. One idea is to put different toys on a table under varying light levels and ask children to pick up a specific toy to assess their ability to function in low light.

*Natural* *history*

1. Systematically assess what information is already available from retrospective cohorts, including images, from various researchers. This important first step would reveal any gaps that a prospective natural history might fill.
2. Build a centralized or federated database of de-identified natural history data, and obtain some interpretation of the images and other analyses of the data. Aggregating these data may also help in the development of gene-independent therapies.
3. To learn the most from images compiled from individual research groups, support artificial intelligence-based image analyses (including OCT images, autofluorescence images, fundus images, widefield images) with the involvement of reading centers.
4. Continue to involve families of affected individuals to help people in the patient community understand the importance of genetic testing and natural history and what they mean to drug development. It is important to recognize that conducting prospective studies is not trivial in terms of the burden on families.
5. Continue to support genetic testing to learn more about the disease mechanisms, including the role of modifier genes.

*Research* *the mechanisms of* *the* *disease*

1. Take the time to generate a large animal model and characterize it. If there is a group interested in generating a large animal model, it would be a very interesting cue to better understand the disease biology and would serve as insurance in case the current strategies are blocked in some ways in terms of moving forward.

In the end, the commitment and vision that were shared by this multi-stakeholder group nurtured the hope of families and laid the foundation for these actions to be accomplished collaboratively and in a timely manner. Ultimately, this initiative has the potential to provide a benchmark model for furthering translational research in the field of rare eye diseases.

## Supplementary Material

Supplement 1

## Appendix A: Invited Workshop Participants and Contributors

Kate Arkell, Retina International, Buckingham, UK

Jean Bennett, University of Pennsylvania, Philadelphia, PA, USA

Silvia Cerolini, Family Patient Representative, London, UK

Wiley Chambers, Center for Drug Evaluation, U.S. Food and Drug Administration, Silver Spring, MD, USA

Janet Cheetham, Foundation Fighting Blindness, Columbia, MD, USA

Jogin Desai, Family Patient Representative, Bangalore, India

Adam Dubis, Moorfields Eye Hospital and University College of London, London, UK

Todd Durham, Foundation Fighting Blindness, Columbia, MD, USA

Blair Ettles, MeiraGTX, New York, NY, USA

Abigail Fahim, University of Michigan, Ann Arbor, MI, USA

Rick Ferris, Foundation Fighting Blindness, Columbia, MD, USA

Mike Fiore, Family Patient Representative, Syossett, NY, USA

Allison Galloway, Family Patient Representative, Westminster, CO, USA

Jessica Imrie, Limelight Bio, Philadelphia, PA, USA

Rusty Kelley, RD Fund of Foundation Fighting Blindness, Columbia, MD, USA

Bart Leroy, Ghent University, Ghent, Belgium

Brian Mansfield, Foundation Fighting Blindness, Columbia, MD, USA

Jason Menzo, Foundation Fighting Blindness, Columbia, MD, USA

Mariya Moosajee, Moorfields Eye Hospital, London, UK

Mathew Pletcher, Family Patient Representative, Basel, Switzerland

Sean Ring, Editas Medicine, Cambridge, MA, USA

Ben Shaberman, Foundation Fighting Blindness, Columbia, MD, USA

Francesca Sofia, Science Compass, Milan, Italy

Debra Thompson, University of Michigan, Ann Arbor, MI, USA

James Tobin, Janssen Pharmaceutical Companies of Johnson & Johnson, Titusville, NJ, USA

Katherine Uyhazi, University of Pennsylvania, Philadelphia, PA, USA

Barbara Wendelberger, Berry Consultants, Austin, TX, USA

Ben Yerxa, Foundation Fighting Blindness, Columbia, MD, USA
