# Supplementary material for: Report from a Workshop on Accelerating the Development of Treatments for Inherited Retinal Dystrophies Associated with Mutations in the RDH12 Gene
Source: Transl Vis Sci Technol. 2020 Jul 17;9(8):30. doi: 10.1167/tvst.9.8.30 (PMC7422783; doi:10.1167/tvst.9.8.30)
Supplement: Supplement 1 [file tvst-9-8-30_s001.docx]

**RETINAL DYSTROPHY ASSOCIATED TO RDH12 GENE MUTATIONS:**

**STATE OF ART AND PERSPECTIVES FOR THE FUTURE**

A scientific and medical landscape analysis

Carried out between March and June 2019 by Dr. Francesca Sofia, PhD (Chief and Founder at Science Compass) and Dr. Cristina Colombelli, PhD (Research Manager at Science Compass)

Contracted by: Silvia Cerolini and Enrico Orlandi

First draft release: July 2019

Final release: October 2019

**CONTENTS**

Foreword

Executive summary

Acknowledgements

1. **Leber Congenital Amaurosis type 13**

1.1 Introduction

1.2 RDH12-associated LCA (LCA13): genetics and clinical manifestations

1.3 Population and market size

1.4 RDH12 gene expression, function and pathomechanism

1.5 RDH12 overall scientific scenario

1. **State of pre-clinical research infrastructures**

2.1 In vitro disease models

2.1.1 iPSCs: an in vitro platform to study retinal dystrophies

2.2 In vivo disease models

2.2.1 Vertebrates

2.2.2 Invertebrates

2.3 Are new RDH12 models worthy and feasible to develop?

1. **Therapeutic Pipeline**

3.1 Introduction

3.2 Gene therapy

3.3 Trophic factors

3.4 Optogenetics

3.5 Antisense oligonucleotides and gene editing

3.6 Pharmacological approaches

3.7 Cell therapies

1. **Clinical assessment**

4.1 Diagnosis

4.2 Clinical studies

4.3 Clinical endpoints

4.4 Medical devices

4.5 Patient registry and biobank

1. **Key messages and gaps identified**

**APPENDIX A** – RDH12 known mutations (September 2019)

**APPENDIX B** – RDH12 Complete list of publications (March 2019)

**APPENDIX C** – Bibliographic analysis performed through Scopus and PubMed in March 2019

**FOREWORD**

Early in January 2019, Silvia Cerolini and Francesca Sofia, Founder and Chief of Science Compass^^[[1]](#footnote-2)^^, discussed the activities of an international network of families who are committed to support scientific research on a rare eye disease, Leber Congenital Amaurosis (LCA) type 13.

The aim of the family network is to foster the identification and development of potential new therapies. To this end, a better understanding of the global scientific landscape of LCA research -including the state of research, the availability of translational research infrastructures and of public and private funding -will be instrumental in planning a coherent strategy focused on the most effective support actions to be undertaken in the future.

The present document is the result of a comprehensive study conducted to meet this need.

**EXECUTIVE SUMMARY**

Leber Congenital Amaurosis (LCA) is a molecularly and clinically heterogeneous group of inherited retinal disorders (IRDs) characterized by vision loss, involuntary eye movements (nystagmus) and severe retinal dysfunction. It is the main cause of inherited childhood blindness, accounting for 5% of all inherited retinal dystrophies. It has high genetic heterogeneity with 25 causative genes identified to date. Among those, *RDH12* -which encodes a photoreceptor-specific retinal dehydrogenase- causes Leber congenital amaurosis type 13 (LCA13).

The pathogenetic mechanism associated with *RDH12* mutations is not completely understood. However, it correlates with loss of RDH12 enzymatic activity and the consequent accumulation of toxic compounds in photoreceptor cells. Over 100 different mutations in RDH12 have been found to cause LCA13 that, in the first years of life, manifests with visual field constriction, loss of visual acuity and night blindness, and inescapably progresses to legal blindness in early adulthood.

At present, there is no treatment for LCA13 representing a relatively new area of scientific investigation. In terms of publications, it accounts for 5.6% of the total scientific production in the field of LCA. Basic and pre-clinical research addressing RDH12 disease mechanisms and/or testing potential therapeutic interventions is hampered by the lack of reliable in vivo and in vitro models. Similarly, and also due to the rarity of LCA13, clinical knowledge of the disease is confined to a few natural history studies conducted by individual clinical and research institutions.

Nevertheless, the field sees several opportunities within a therapeutic pipeline that presently involves a variety of different possible approaches from gene therapy - which is the most promising and advanced one in the path to a cure, to other approaches. Among those, neuroprotective strategies either involving gene therapy or neuroprotective compounds, may slow down disease progression and provide a wider time window for treatment. Efforts are also being made in the field of optogenetics which aims at vision restoration by precisely exciting the neural apparatus. In addition, cell replacement therapies are being explored in IRDs and could, therefore, be applied to RDH12 although several concerns need yet to be addressed. Finally, gene editing and antisense oligonucleotides may provide additional means to tackle the dominant forms of the disease.

In order to make the most of these opportunities, some major gaps need to be filled. For example, mouse models developed so far do not recapitulate the human phenotype and better models would be needed. Expectations are growing from iPSCs and organoids which would be extremely valuable for in vitro pre-clinical testing of new therapeutic approaches. At the same time, multi-center longitudinal observational studies would be beneficial

The present study addresses all aspects of RDH12 scientific research and depicts the state of the art while also highlighting gaps and opportunities. Its ultimate aim is to allow strategic planning and identification of the most feasible steps going forward. It can also be used as a tool for stakeholder engagement.

**ACKNOWLEDGMENTS**

We are deeply thankful to the experts who devoted their time to this study by participating in one-to-one interviews that were held via web-conference between May and June 2019.

List in alphabetical order:

**Tomas Aleman -** Scheie Eye Institute, The Children's Hospital of Philadelphia, the Center for Advanced Retinal and Ocular Therapeutics (CAROT), Perelman School of Medicine, University of Pennsylvania, Philadelphia, Pennsylvania.

**Jean Bennett -** Center for Advanced Retinal and Ocular Therapeutics, Scheie Eye Institute, University of Pennsylvania Perelman School of Medicine, Philadelphia, PA, USA

**Li Bin -** Department of Ophthalmology Shiyan, Hubei University of Medicine - Taihe Hospital, Hubei. China

**Jogin Desai -** founder and CEO at Eyestem Research

**Abigail T. Fahim -** Department of Ophthalmology and Visual Sciences, University of Michigan, Ann Arbor, Michigan, USA

**Krzysztof Palczewski** - Department of Ophthalmology, Gavin Herbert Eye Institute, University of California, Irvine, California, USA

**Mathew Pletcher -** Head of Rare Disease – Discovery Roche, Basel, Switzerland

**Jose-Alain Sahel** - Department of Ophthalmology, University of Pittsburgh School of Medicine, Pittsburgh, PA, USA and Sorbonne Université, INSERM, CNRS, Institut de la Vision, Paris, France

**Hendrik Scholl -** Wilmer Eye Institute, Johns Hopkins University, Baltimore, MD, USA. Institute of Molecular and Clinical Ophthalmology Basel (IOB) and Department of Ophthalmology, University of Basel, Basel, Switzerland.

**Ruifang Sui -** Department of Ophthalmology, Peking Union Medical College Hospital, Peking Union Medical College, Chinese Academy of Medical Sciences, Beijing, China

****************************

Our thanks go also to those who did not attend formal interviews but provided us with updates about their ongoing research efforts in the field:

**Robin Ali,** UCL Institute of Ophthalmology, London, UK

**Robert Molday**, Department of Biochemistry and Molecular Biology, University of British Columbia, Vancouver, British Columbia, Canada

**Anne Kasus-Jacobi**, Department of Pharmaceutical Sciences, Department of Physiology and Oklahoma Center for Neuroscience, University of Oklahoma Health Sciences Center, Oklahoma City, OK, USA

**Debra A. Thompson**, University of Michigan Medical School, Department of Ophthalmology and Visual Sciences and Department of Biological Chemistry, Ann Arbor, Michigan, United States

**Stephen H. Tsang**, Department of Ophthalmology, Department of Pathology and Cell Biology and Jonas Children's Vision Care, Bernard and Shirlee Brown Glaucoma Laboratory, Columbia University, New York, NY, USA

**1. LEBER CONGENITAL AMAUROSIS TYPE 13**

**1.1 Introduction**

The term Leber congenital amaurosis (LCA) was introduced by Theodore Leber in 1869 to describe a group of very early-onset rod-cone dystrophies. Some years later, Leber described what he considered a milder form of the disease which has gotten different names thereafter including early-onset severe retinal dystrophy (EOSRD), severe early childhood-onset retinal dystrophy (SECORD) and early-onset retinitis pigmentosa (RP).

LCA and EOSRD comprise a molecularly and clinically heterogeneous group of inherited retinal disorders (IRDs) characterized by vision loss, involuntary eye movements (nystagmus) and severe retinal dysfunction. The onset is in the first months of life. However, EOSRD can also present early in infancy, generally before the age of 5 years. Other clinical manifestations may be present and include high hypermetropia, markedly reduced or absent rod and cone responses at the electroretinogram (ERG), light-sensitivity (photodysphoria), eye poking (oculodigital sign), cone-shaped cornea (keratoconus), cataracts, and a variable appearance of the fundus (eg. waxy optic disc, attenuation of the arterioles). Better residual visual function and small ERG signals usually characterise EOSRD.

Although people with LCA/EOSRD usually present with isolated ocular signs and symptoms, some infants with severe early vision deficits may experience speech delay and behavioural difficulties.

LCA is the leading cause of inherited childhood blindness, accounting for 5% of all inherited retinal dystrophies. It has high genetic heterogeneity with 25 causative genes identified to date (**Table 1**) and is typically inherited as an autosomal recessive genetic condition. However, a few cases of autosomal dominant inheritance have been reported (for example, heterozygous pathogenic variants in CRX, OTX2 and IMPDH1). Though rare, those cases must be carefully addressed when it comes to design and test of potential therapeutic approaches since haploinsufficiency and dominant negative mutations have very different pathomechanisms.

The majority of LCA genes are expressed solely or predominantly in the retina or in the retinal pigment epithelium (RPE), and code for proteins with various retinal functions including phototransduction and visual cycle, and photoreceptor development and integrity.

There is significant overlap between the molecular causes of LCA and EOSRD, with some genes causing both clinical phenotypes. Nonetheless, certain genes are more frequently associated with LCA, like GUCY2D, NMNAT1, CEP290 and AIPL1, whereas mutations in others including RPE65, LRAT and RDH12, more commonly result in an EOSRD phenotype.

Different mutations in the same LCA/EOSRD-associated genes are known to cause other retinal dystrophies like retinitis pigmentosa (RP) and cone-rod dystrophy (CORD). **Therefore, it is broadly shared that LCA, EOSRD, RP and CORD belong to the same spectrum of retinal abnormalities with LCA-associated loss-of-function mutations lying at the severe end of the spectrum.**

| **Locus name** | **Gene** | **Protein** | **Function** | **Frequency** |
| --- | --- | --- | --- | --- |
| LCA1 | *GUCY2D* | Guanylate cyclase-1 | Phototransduction | 10-20% |
| LCA2 | *RPE65* | Retinoid isomerase | Retinoid cycle | 5-10% |
| LCA3 | *SPATA7* | Spermatogenesis-associated protein 7 | Photoreceptor ciliary transport | 3% |
| LCA4 | *AIPL1* | Aryl-hydrocarbon-interacting-protein-like 1 | Phototransduction/ protein biosynthesis | <5% |
| LCA5 | *LCA5* | Libercilin | Photoreceptor ciliary transport | 1-2% |
| LCA6 | *RPGRIP1* | Retinitis pigmentosa GTPase regulator-interacting protein 1 | Photoreceptor ciliary transport | 5% |
| LCA7 | CRX | Cone-rod homeobox | Photoreceptor morphogenesis | 1% |
| LCA8 | *CRB1* | Crumbs homologue 1 | Photoreceptor morphogenesis | 10% |
| LCA9 | *NMAT1* | Nicotinamide nucleotide adenyltransferase1 | Coenzyme NAD biosynthesis | Unknown |
| LCA10 | *CEP290* | Centrosomal protein 290 kDa | Photoreceptor ciliary transport | 15-20% |
| LCA11 | *IMPDH1* | Inosine 5’-monophosphate dehydrogenase 1 | Guanine synthesis | 5% |
| LCA12 | *RD3* | Retinal degeneration 3 | Protein trafficking | <1% |
| **LCA13** | ***RDH12*** | **Retinol dehydrogenase 12** | **Retinoid cycle** | **3.4-10.5%** |
| LCA14 | *LRAT* | Lecithin:retinol acyl transferase | Retinoid cycle | <1% |
| LCA15 | *TULP1* | Tubby-like protein | Photoreceptor ciliary transport | <1% |
| LCA16 | *KCNJ13* | Kir7 inwardly rectifying potassium channel | Phototransduction | Unknown |
| LCA17 | *GDF6* | Growth differentiation factor 6 | Photoreceptor morphogenesis | Unknown |
|  | *OTX2* | Orthodenticle homeobox 2 protein | Photoreceptor differentiation | Unknown |
|  | *CABP4* | Calcium-binding protein 4 | Phototransduction | Unknown |
|  | *CLUAP1* | Clusterin-associated protein 1 | Photoreceptor ciliary transport | Unknown |
|  | *IQCB1 or NPHP5* | IQ motif containing B1 protein | Photoreceptor ciliary transport | Unknown |
|  | *DTHD1* | Death-domain containing protein 1 | Signaling/Apoptosis pathway | Unknown |
|  | *IFT140* | Intraflagellar transport 140 chlamydomonas homologue protein | Photoreceptor ciliary transport | Unknown |
|  | *ALMS1* | ALMS protein | Photoreceptor ciliary transport | Unknown |
|  | *PRPH2 or RDS* | Peripherin/Retinal degeneration slow | Photoreceptor OS structure/stabilisation | Unknown |

**Table 1.** List of identified LCA genes

*Modified from Kumaran et al., 2017 and GeneReviews 2018 (*<https://www.ncbi.nlm.nih.gov/books/NBK531510/>*)*

**1.2 RDH12-associated LCA (LCA13): genetics and clinical manifestations**

Leber congenital amaurosis type 13 (LCA13) is caused by homozygous or compound heterozygous mutations in *RDH12*, encoding a photoreceptor-specific retinal dehydrogenase. Currently there are over 100 known *RDH12* mutations (HGMD®), including deletions, missense, nonsense, frameshift, and splice site mutations (see Appendix A for the complete list of mutations). LCA13 is a severe retinal dystrophy, with typical onset in the first years of life (2-4 years) and progression to legal blindness in early adulthood (18-25 years). Visual field constriction, loss of visual acuity and night blindness are generally reported at diagnosis. Visual function is usually poor, yet useful in early life, followed by progressive decline and often accompanied by nystagmus, sluggish or near-absent pupillary responses, mild hyperopia and keratoconus. The retinal architecture is distorted, precluding the identification of the normal laminae (Jacobson et al., 2007), and is characterized by: massive accumulation of bone spicule pigment in the peripheral retina, RPE atrophy and macular scarring. One feature of *RDH12*-associated disease is represented by early-dense intraretinal pigmentation and early progressive macular atrophy with pigmentation, yellowing and loss of fundus autofluorescence (Mackay et al., 2011).

Two recent publications report peripapillary sparing as a novel phenotypic feature in a large proportion of *RDH12*-lead LCA cases (Garg et al., 2019; Fahim et al., 2019). Peripapillary sparing is usually considered pathognomonic of Stargardt disease, a juvenile-onset maculopathy caused by mutations in the ATP Binding Cassette Subfamily A Member 4 (*ABCA4*) gene, that results in the accumulation of toxic bisretinoid-containing lipofuscin in the RPE (Cideciyan et al., 2005). **The presence of areas of preserved retina in *RDH12*-associated disease might be relevant for the development of therapeutic strategies.**

Of note, mutations in *RDH12* have been also associated with relatively milder phenotypes classified as juvenile-onset retinitis pigmentosa (RP53), early-onset retinal dystrophies (EORD), and rarely CORD. RP53 is a retinal dystrophy belonging to the group of pigmentary retinopathies. Retinitis pigmentosa is characterized by retinal pigment deposits visible on fundus examination and primary loss of rod PR cells followed by secondary loss of cone PRs. Patients typically have night vision blindness and loss of mid-peripheral visual field. As the condition progresses, they lose their far peripheral visual field and eventually central vision as well. RP53 inheritance is generally autosomal recessive, though rarely *RDH12* can segregate with a milder phenotype in an autosomal dominant fashion: Fingert and co-authors, found that 776delG mutation might exert a gain-of-function or dominant negative effect (Fingert et al., 2008).

**1.3 Population and market size**

The prevalence of LCA/EOSRD is between 1/33,000 and 1/81,000 live births and accounts for 5% of all IRDs and 20% of blindness in school age children (Koenekoop, 2004; Stone, 2007; Orphanet). This disorder affects males and females in equal numbers.

The identified genes (**Table 1**) account for 70-80% of all LCA/EOSRD cases, with *GUCY2D*, *CEP290*, *CRB1*, *RDH12* and *RPE65* being the most common (Kumaran et al., 2017). Frequency of *RDH12* gene mutations in LCA/EOSRD patients varies among published papers with the most recent estimate being 3.4%‐10.5% (Fahim et. al, 2019).

According to key opinion leaders (KOLs) who agreed to be interviewed for the purposes of this landscape analysis ***RDH12*-related degeneration is equally distributed worldwide** with patients in Asia, USA and various European countries. Seemingly higher rates have been noted in India. In China there are currently 100 children with a diagnosis of *RDH12*-associated LCA (Ruifang Sui) and the USA present a similar situation (M. Pletcher). An unspecified but seemingly significant number of RDH12 patient is also monitored in Paris (Jose-Alain Sahel).

**1.4 RDH12 gene expression, functions and pathomechanism**

**Gene.** *RDH12* gene, also known as LCA13, RP53 or SDR7C2**,** was cloned in 2002 (Haeseleer et al., 2002) by the laboratory of Krzysztof Palczewski at Case Western Reserve University, Cleveland, OH (USA). It maps to LCA13 locus, on human chromosome 14 (14q23.3-24.1). It is approximately 12 kbs in length and consists of seven coding regions (exons) (**Figure 1**).


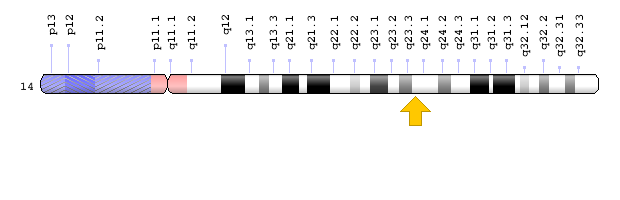


**Figure 1**: Cytogenetic location: 14q24.1, Molecular location: base pairs 67,701,886 to 67,734,451 on chromosome 14 (Homo sapiens Annotation Release 109, GRCh38.p12). From *Genetics Home Reference NCBI.*

RDH12 is expressed predominantly in the eyes, with the highest expression

levels observed in the retina. In situ hybridization in monkey and mouse retinas indicated that RDH12 localizes to the inner segments of rod and cone photoreceptors (Haeseleer et al., 2002; Kurth et al., 2007; Maeda et al., 2006). RDH12 expression is also detected in the kidneys, brain (pineal gland), skin, skeletal muscle, liver and stomach. Interestingly, it has been found that RDH12 transcription is tightly regulated in a circadian clock-dependent manner (Vancura et al., 2018). The rhythmic expression of RDH12 suggests the presence of a daily adjustment of retinal function so that vision can adapt to the alternating day-night cycle. Specifically, RDH12 is upregulated at the earlier times of the day and this may fulfill the demand to protect photoreceptors from increasing concentrations of retinaldehydes released during light exposure/daytime (Vancura et al., 2018).

**Protein**. RDH12 is a 316 amino acid-long enzyme with a molecular mass of about 35 kDa. It localizes intracellularly to the endoplasmic reticulum (ER) (<https://www.uniprot.org/uniprot/Q96NR8#subcellular_location>) with the majority of its polypeptide chain exposed on the cytosolic side of the membrane (Lee et al., 2010).


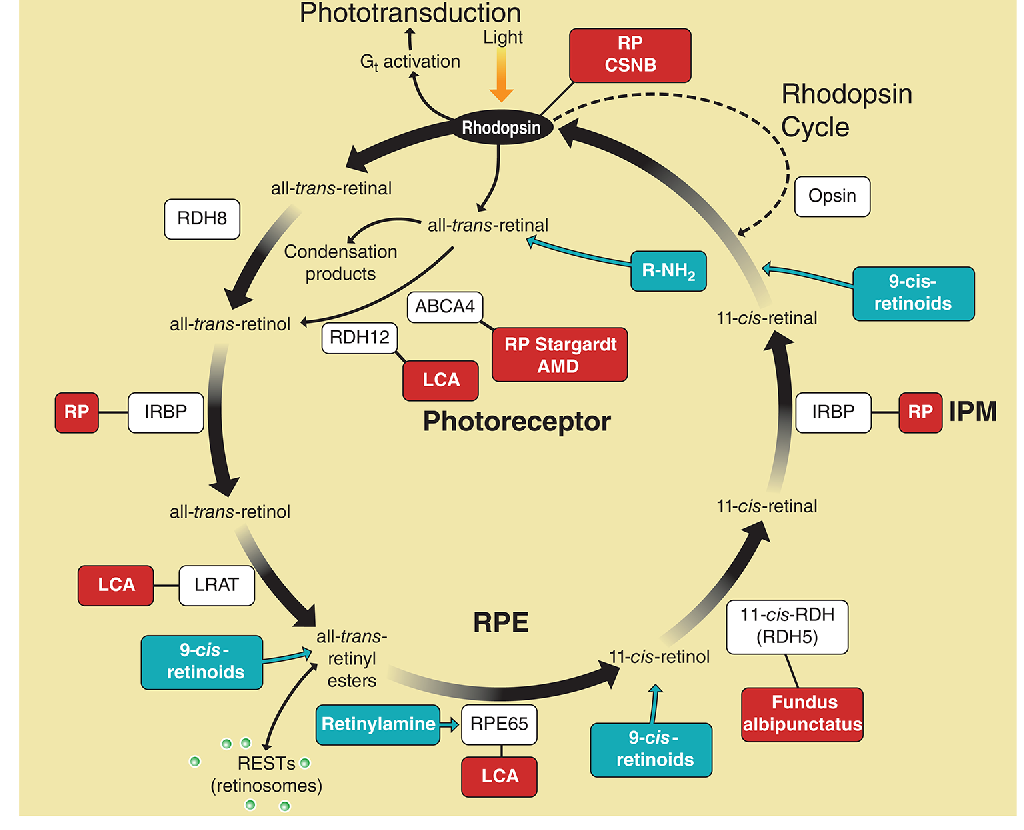
 RDH12 is a member of the short chain alcohol dehydrogenases/reductases (SDR) family (Jornvall et al., 1995) and functions as a dual-specificity dehydrogenase that metabolizes photosensitive vitamin A derivatives known as retinol and retinal, which are important for light detection (Haeseleer et al., 2002). Particularly, RDH12 exhibits its highest activity toward 9-cis and all-trans-retinol. The biologic events that lead to visual perception begin when light is absorbed by cone opsins and rhodospin in the retina and triggers the isomerization of 11-cis retinal (11-cis RAL) to all-trans retinal (all-trans-RAL). This reaction initiates the transmission of visual information as a chemical signal. For continued signal transduction, all-trans-RAL must be converted back into 11-cis RAL through a series of enzymatic steps known as the visual or retinoid cycle. RDH12 plays an important role in this cycle by catalyzing the NADPH-dependent reduction of all-trans-RAL to all-trans retinol (ROL). The same reaction is also catalyzed by RDH8, another member of the SDR family located in the photoreceptor’s external segment.

**Figure 2** The visual cycle. Adapted from Kiser et al., 2014

Once generated, all-trans-ROL is transported to the RPE and converted to 11-cis RAL by a transporter protein and at least three enzymes: interphotoreceptor retinoid binding protein (IRBP/RBP3), lecithin:retinol acyl transferase (LRAT), retinoid isomerohydrolase (RPE65), and 11-cis retinol dehydrogenase (11-cis RDH/RDH5 and RDH11) (**Figure 2**).

**The importance of the visual cycle is emphasized by the large number of retinal disorders associated with mutations in the genes coding for these proteins**: high myopia with retinal dystrophy or autosomal recessive retinitis pigmentosa (RP66) for *IRBP*, LCA14/EOSRD and juvenile retinitis pigmentosa for *LRAT*, LCA2 and RP20 for *RPE65*, and fundus albipunctatus, a mild form of stationary night blindness, for *RDH5* (**Table 2**).

| **Gene** | **Protein** | **Disease** |
| --- | --- | --- |
| *IRBP*/*RBP3* | Interphotoreceptor retinoid binding protein | - High myopia with retinal dystrophy  - Retinitis pigmentosa 66 (arRP66) |
| *LRAT* | Lecithin: retinol acyl transferase | - LCA14/EOSRD  - Juvenile retinitis pigmentosa |
| *RPE65* | Retinoid isomerohydrolase | - LCA2  - Retinitis pigmentosa 20 (RP20) |
| *11-cis RDH*/*RDH5* | 11-cis retinol dehydrogenase | Fundus albipunctatus |
| *RDH12* | Retinol dehydrogenase 12 | - LCA13/EOSRD  - Retinitis pigmentosa 53 (RP53) |
| *ABCA4* | ATP Binding Cassette subfamily A member 4 | - Stargardt disease 1 or Fundus flavimaculatus  - arRP19  - CORD3  - EOSRD |
| *RGR* | Retinal G protein–coupled receptor | Retinitis pigmentosa 44 |
| RLBP1/CRALBP | Retinaldehyde binding protein 1 | - Retinitis punctata albescens  - Fundus albipunctatus  - arRP Bothnia type  - Newfoundland rod-cone dystrophy |

**Table 2** Diseases linked to changes in visual cycle proteins. *Modified from Travis et al., 2008*

RDH12 is also involved in the metabolism of other non-retinoid alcohols/aldehydes, like *N-*retinylidene-*N*-retinylethanolamine (A2E), and toxic products resulting from light-induced lipid peroxidation, including 4-hydroxynonenal (4-HNE). By detoxifying these compounds RDH12 protects photoreceptors (PRs) against light-induced apoptosis (Maeda et al., 2007; Marchette et al., 2010). Indeed, the loss of RDH12 activity is likely to increase sensitivity of PR cells to oxidative injury, contributing to the retinal degeneration phenotype of patients with *RDH12* mutations.

**Pathomechanisms.** The pathogenetic mechanism associated with *RDH12* mutations is not completely understood. However, it correlates with loss of RDH12 enzymatic activity since mutant RDH12 proteins associated with LCA/EOSRD are inactive or display only residual activity (Janecke et al., 2004; Sun et al., 2007; Thompson et al., 2005). Only a few mutations were reported to retain enzymatic activity, at least *in vitro*, but lead to accelerated degradation via the proteasome (Lee et al., 2010).

Most of the KOLs interviewed in the study agreed that the main pathogenetic mechanism of the disease has more to do with the accumulation of retinaldehydes in the inner segment of the retina, in conditions of continuous light exposure, than with impairment of the visual cycle. Indeed, in the absence of a functional RDH12, components of the visual cycle are not processed and accumulate thus leading to toxicity.

**1.5 RDH12 overall scientific scenario**

LCA13 is a relatively new area of investigation within the inherited retinal disorders’ field. It was prompted by the discovery of the responsible gene, RDH12, in 2002 and saw a progressive though moderate growth of interest from the scientific community (**Figure 3**).

In March 2019, **98 bibliographic items** (see Appendix C for the complete list) could be retrieved by Scopus^^[[2]](#footnote-3)^^ search using “RDH12” as keyword within titles, abstracts, and papers’ keywords. This figure corresponds to **5,6% of the total scientific production in the LCA field** (totaling 1757 publications, Scopus, March 2019).


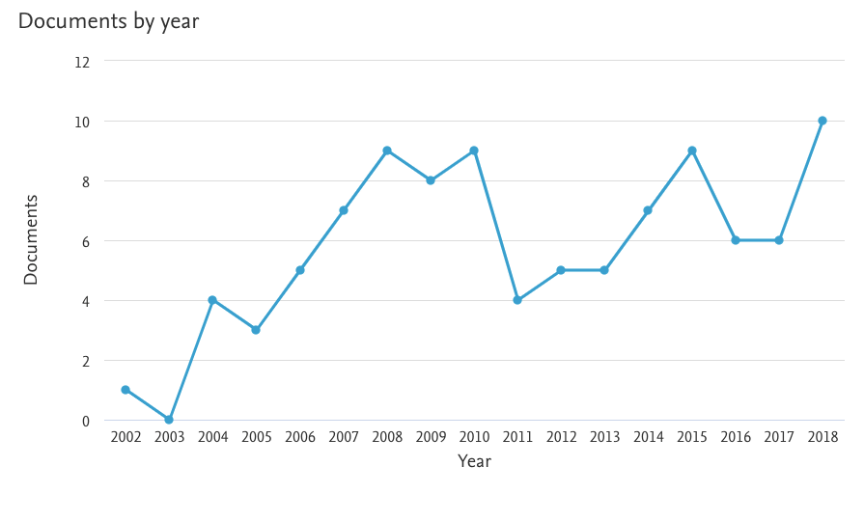


**Figure 3.** Number of papers addressing RDH12 per year of publication

Major players, in terms of number of publications in the RDH12 research field, are investigators in Ophthalmology and Biochemistry with the following being the top three in the ranking:

1) **Debra A. Thompson** (11 publications) - professor of Ophthalmology and Visual Science and of Biological Chemistry at the Kellogg Eye Center, Ann Arbor, MI (US). Her research focuses on the molecular mechanisms responsible for inherited forms of retinal degeneration.

2) **Olga V. Belyaeva** - senior researcher in the lab of **Natalia Kedishvili**, University of Alabama at Birmingham. Their work focuses on the enzymes and the mechanisms that control the biosynthesis of retinoic acid, the bioactive derivative of vitamin A.

3) **Andreas Gal**, MD, at the University of Hamburg and Institut für Humangenetik, Universitätsklinikum Hamburg-Eppendorf, Germany.

Research on RDH12 localizes predominately in the USA (53 publications) and Europe (above 30 publications) with China, India and Japan contributing for the remaining.

In terms of subject area, the vast majority of publications falls in the fields of: 1) Medicine (36,4%) comprising mainly clinical and case reports, 2) Biochemistry (30,5%) and 3) Neuroscience (14,9%).

Original data from Scopus and PubMed searches are reported in Appendix C.

**2. STATE OF PRECLINICAL RESEARCH INFRASTRUCTURES**

**2.1 *In vitro* disease models**

Apart from cell lines expressing mutant *RDH12* or somatic cells derived from affected patients (fibroblasts, keratinocytes, blood cells), to date no effective *in vitro* model for *RDH12*-associated disease is available.

Several different approaches to develop *in vitro* models of PR cells are under investigation and those are:

1. Whole retinal explants/organotypic cell cultures;
2. PRs from induced pluripotent stem cells (iPSCs) reprogrammed from patients-derived somatic cells: they can be the basis of a personalized medicine approach and can be used to screen drug libraries and identify therapeutics for specific patients;
3. PRs derived from human/murine embryonic stem cells (ESCs) (Considerable data and several protocols have been published regarding differentiation of ESCs *in vitro* toward photoreceptor lineages).

**2.1.1 iPSCs: an *in vitro* platform to study retinal dystrophies**

The mammalian retina does not regenerate after damage, therefore biopsies of the posterior segment of the eye cannot be obtained from living patients. Hence, affected ocular tissues are derived only post-mortem, usually at advanced stages of the disease. That is why they are not ideal tools for studying disease pathogenesis or testing potential therapeutic interventions. Instead, a good *in vitro* model for retinal disorders is represented by induced Pluripotent Stem Cells^^[[3]](#footnote-4)^^ (iPSCs). These can form embryoid bodies containing cells generating all three germ layers and can be used for disease modelling, drug screening and/or regenerative medicine.

iPSCs are very similar to Embryonic Stem Cells (ESCs) in that they have the potential to give rise to virtually every body tissue. iPSCs have been generated for several retinal inherited disorders thanks to their ability to self-organize and differentiate into relevant cell types, like RPE cells and photoreceptors.

In the last few years, protocols for the differentiation of RPE cells and the generation of pure populations of photoreceptor-like cells have been optimized (Chen et al., 2014; Barnea-Cramer et al., 2016). Moreover, 3D multilayer eyecup-like structures with retinal precursor cells and PR cells capable of responding to light have been developed (Zhong et al., 2014).

Patients-derived iPSCs have been recently established for various types of LCA, RP, Usher syndrome, Best disease and gyrate atrophy. Examples are:

- RPE65-LCA: patient-specific iPSCs were used to confirm the pathogenicity of a previously unknown intronic mutation in *RPE65* (Tucker et al., 2015);

- AIPL1-LCA and RP caused by CRB1 mutations: established human iPSC lines (Lukovic et al., 2018; Zhang et al., 2018);

- LCA5: LCA5 patient-derived iPSC-RPEs were used as a proof-of-concept for the restoration of lebercillin protein and the rescue of cilia defects upon *LCA5* cDNA delivery (Song et al., 2018);

- X-linked RP caused by *RP2* nonsense mutations: RPE cells derived from patient-specific iPSCs were treated with translational read-through inducing drugs and up to 20% of normal RP2 protein can be restored with reversal of the cellular phenotype defects (Schwartz et al., 2015);

- autosomal recessive RP caused by an intronic variant of *USH2A* gene: iPSCs were derived from patient’s keratinocytes and differentiated into multilayer eyecup-like structures with features of PR precursor cells. The latter were transplanted in an immunodeficient mouse to confirm the pathogenicity of the mutation (Tucker et al., 2013);

- *BEST1* mutations causing Best disease, characterized by a severe maculopathy: RPE cells were derived from patient-specific iPSCs and used to determine the role of bestrophin in the regulation of intracellular calcium stores (Singh et al., 2013);

- *CEP290*-LCA caused by an intronic mutation: 3D optic cups with opsin-expressing PRs were generated and used to demonstrate the pathogenicity of aberrant *CEP290* splicing, which was corrected by specific antisense oligonucleotide (Parfitt et al., 2016);

- mutations in RPGR causing X-linked RP: patient-specific iPSCs were differentiated in RPE and PRs, revealing the pathomechanism of the disease (Megaw et al., 2017).

An important consideration concerning the use of iPSCs is the development of iPSC control lines; they can be derived from different persons who don’t have the disease, but this can introduce confounders due to different genetic background or differences in the processes of iPSC reprogramming or differentiation. It would be better to use gene editing techniques that enable the creation of control lines identical to the patient ones except for the mutation in the gene of interest.

**Even though the examples of patient-specific iPSC lines in retinal dystrophies are abundant, there are no published data on RDH12-iPSCs yet.** However, a project funded last year by the RDH12 family network through Retina UK is aiming at developing stem cells derived from patients with *RDH12* mutations. The project is led by Dr Mariya Moosajee at UCL Institute of Ophthalmology, London (UK). According to the first-year report, Dr. Moosajee and collaborators have taken skin biopsies from two adult patients with *RDH12* mutations and a blood sample from a child with the condition^^[[4]](#footnote-5)^^. So far, they reprogrammed one patient's skin cells into stem cells. For the second patient the process is underway, as is for the blood sample, which requires the application of a more complex methodology. The next step will be to convert the stem cells into early eye cups in a dish. This project has the potential to pave the way for the generation of various lines of patient-derived iPSCs so that mutation-specific therapies can be developed.

KOLs interviewed in this study agreed that iPSCs represent an ideal model for enabling translational research in RDH12. The scientific community is gaining expertise that will make iPSC-derived 3D retina a reality in the years to come. However, one KOL identified a major limitation with this approach - that PR derived from iPSCs cannot interact with the RPE.

Additional information gathered during KOLs’ interviews regard RDH12 patient-specific iPSC lines being developed by:

***- Jogin Desai – Dr. Desai started to develop an iPSC line for an RDH12 nonsense mutation. For the time being, he has started with one patient only, but in the future the number of patients might increase as the incremental costs of the procedure would make it affordable;***

***- Jean Bennett – Dr. Bennet is going to develop RDH12 patient-specific iPSCs to be differentiated into PRs both to study the disease and to demonstrate that the gene therapy she is developing is working (proof-of-concept study);***

***- Ruifang Sui – during the interview, Dr. Sui briefly mentioned that they are developing iPSCs, however no further detail was added.***

**2.2 *In vivo* models**

**2.2.1 Vertebrates**

**Mouse.** Mice are generally used as convenient animal models of many human disorders, including retinal dystrophies. For RDH12, two murine lines not expressing *Rdh12*, also known as knockout (KO) or null, were generated by two independent research groups:

1. the *Rdh12* KO mouse generated by Krzysztof Palczewski’s lab in Cleveland, OH, US (Maeda et al., 2006), with deletion of exons 1-3;
2. the *Rdh12* KO mouse created by Hübner’s lab in Hamburg, DE (Kurth et al., 2007), with deletion of exons 1-3, coding one third of the protein N-terminal.

Both *Rdh12* KO mice have been described to be more susceptible to PR light-induced apoptosis, i.e. they show higher vulnerability to the toxic effect of light (with experimental light conditions equivalent to daylight brightness). **However, they do not exhibit overt retinal degeneration or major slowing of the visual cycle**; in fact, they have a normal regeneration of visual pigments, with only a delay of dark adaptation due to slower kinetics of all-trans-RAL reduction (Kurth et al., 2007; Maeda et al., 2006 and 2007). Hence, their phenotype is milder than that of humans carrying *RDH12* null alleles, and the retinal pathology does not resemble LCA exactly. As an explanation, redundancy of other murine Rdhs or substantial biological differences of mouse versus human RDH12 was suggested (Maeda et al., 2007). Nonetheless, redundancy can be excluded for two main reasons: different cellular and subcellular localizations of different Rdh isoforms (**Table 3**) and only mild abnormalities and minor effects on chromofore synthesis in *Rdh8/Rdh12* double and *Rdh5/Rdh8/Rdh12* triple KO mice (Maeda et al., 2007). Rather, it is more likely that human PRs are more susceptible than murine ones because they need a faster regeneration of rhodopsin after bleaching, i.e. a higher rate of 11-cis RAL delivery.

To better understand the role of RDH12 in the retina, researchers have investigated other functions. For example, it was shown that RDH12 protects inner segments of PRs from leakage of retinal or other toxic aldehydes from the outer segments during light exposure (Chen et al., 2012). In particular, free all-trans RAL can condense into the lipofuscin component A2E, which is highly accumulated in *Rdh12*KO mice retinae and increases with aging (Chrispell et al., 2009); this and other very reactive aldehydes might induce toxic effects on mitochondria and other vital organelles within the inner segments of PRs (Maeda et al., 2006; Chen et al., 2012). Another toxic compound that is metabolized by RDH12 is 4-HNE, a molecule that accrues in the presence of oxidative stress and upon light exposure. In these conditions, it enables the formation of protein adducts in the inner segments; an early event that leads to PR cell death. Indeed, it was shown that *Rdh12* KO mouse is significantly more affected than the wild type by exposure to bright light (Marchette et al., 2010). Therefore, RDH12 loss-of-function relative to detoxifying 4-HNE could contribute to the pathogenetic mechanism of LCA associated with *RDH12* mutations, as absence of endogenous protection could lead to a progressive accumulation of 4-HNE during daily exposure to moderate light.

Even though *Rdh12* KO mice shed light on the role of RDH12 in the retina, it is not the best model for LCA13. As a matter of fact, it is difficult to model a phenotype that is triggered by bright light in animals like rodents that are nocturnal in nature, thus exposed to low levels of illumination (they have slower retinoid regeneration kinetics vs. humans and they have less cones). Thus, alternative animal models should be generated.

| **RDH isoform/disease** | **Localization** | **Function** | **Mouse model features** |
| --- | --- | --- | --- |
| **RDH8** (prRDH)  No pathologic mutation | Photoreceptor Outer Segments (OS) | all-trans-RAL 🡪 all-trans-ROL  in OS,  at higher light intensities and prolonged illumination | *Rdh8*KO |
|  |  |  | Mild phenotype:  - no structural retinal abnormalities  - slower clearance of all-trans-RAL  - slight increase of A2E  - delayed dark adaptation  - normal level of regenerated visual pigment (auxiliary role in visual cycle) |
| **RDH5** (11-cis RDH)  Fundus albipunctatus | RPE | 11 cis-ROL 🡪 11 cis-RAL | *Rdh5*KO |
|  |  |  | Mild phenotype:  - delayed dark adaptation after intense light  - normal level of regenerated visual pigment  - no retinal degeneration |
| **RDH10** | RPE, Müller glia | 11 cis-ROL 🡪 11-cis RAL | RPE conditional *Rdh10*cKO |
|  |  |  | - normal retinal morphology  - delayed regeneration of 11 cis-RAL  - delayed dark adaption after bright light |
| **RDH12**  LCA, EOSRD, RP | Photoreceptor Inner Segments (IS) | all-trans-RAL 🡪 all-trans-ROL  - Reduction of A2E and 4-HNE  - Activity for trans-, cis-RALs, and aldehydes | *Rdh12*KO |
|  |  |  | - no gross retinal abnormalities  - slower clearance of all-trans RAL  - delayed dark adaptation  - accelerated 11 cis-RAL synthesis  - increase susceptibility to light-induced photoreceptor apoptosis |
| **RDH11** (11-cis RDH)  Syndromic retinitis pigmentosa | RPE, photoreceptor IS | 11 cis-ROL 🡪 11-cis RAL  - Reduction of 4-HNE *in vitro* only | *Rdh11*KO |
|  |  |  | - no obvious retinal defect  - delayed dark adaptation following high bleaching level  - kinetics of 11-cis RAL not altered |
| **RDH13** and **RDH14** | Photoreceptor IS (mitochondria) | all-trans-RAL🡪 all-trans-ROL (Rdh13 and Rdh14) | *Rdh13*KO |
|  |  |  | - *Rdh13* protects the retina against acute light-induced retinopathy |

**Zebrafish.** Owing to ∼70% sequence identity between human and zebrafish orthologous genes and ease of genome manipulation, *Danio rerio*, or zebrafish, is a powerful vertebrate model to study physiology and disease. Not being a nocturnal animal makes it more convenient than rodents in modelling retinal disorders. Other advantages include a human-like cone-dominated retina, rapid eye development with functional vision by 5 days post fertilization (dpf), visual function assays on a large number of animals, and easy retinoid quantification in the eye.

**Table 3** RDH isoforms in the retina. RPE: retinal pigment epithelium; OS: outer segment; IS: inner segment; KO: knockout. Also generated: *Rdh5/Rdh10* dKO; *Rdh5/Rdh11* dKO; *Rdh8/Rdh12* dKO; Rdh5/Rdh8 dKO; Rdh5/Rdh8/*Rdh12* tKO*; Rdh8/Abca4* dKO*; Rdh8/Rdh12/Abca4 t*KO.

Gene knockdown with morpholino oligonucleotides is usually performed, however, transient silencing and discrepancies between knockdown and knockout models highlight the need of alternatives in zebrafish model synthesis. Recently CRISPR/Cas9 in zebrafish has emerged as a technology enabling high efficiency targeted gene knockout, patient-specific knockins, activation, or silencing to single base-pair resolution (Ward et al., 2018).

By searching PubMed to determine if any zebrafish mutants in RDH genes had been previously generated, it was found that Rdh5 morphants have been generated by morpholino knockdown and were characterized; they revealed the presence of retinal coloboma and ventral retinal abnormalities including abnormal development of RPE and PR cells (Nadauld et la., 2006). Interestingly, in the same work, Rdh12 expression was detected in the eye of 72 hours post fertilization (hpf) wild type larvae (Nadauld et al., 2006). Rdh12 was found to be slightly expressed also in retinal ganglion cell layer at larval day 5 (<http://zfin.org/ZDB-FIG-060810-745>). Furthermore, Rdh10a deficient zebrafish embryos were also generated, but no detailed description of the eye was provided in the paper (d’Aniello et al., 2015).

Zebrafish *rdh12* is located on chromosome 13, consisting of a 7-exon (1422 bp) gene producing the 320 amino acid protein. It is comparable in size to the human ortholog, which is 316 amino acids. Zebrafish *rdh12* gene shares 71% homology with human *RDH12,* and there is 64% protein sequence homology.

At the Zebrafish International Resource Center there are two rdh12 mutated lines: one with a LacZ insertion and the second with a point mutation that causes premature STOP (<https://zfin.org/ZDB-GENE-040718-9>).

Dr Mariya Moosajee and her team at Moorfields Eye Hospital are developing a zebrafish disease model that will allow the screening of drugs which were approved for unrelated conditions; this approach can potentially be used for a range of inherited retinal diseases. As confirmed by their first report (April 2019), they have used CRISPR/Cas9 editing techniques to introduce a mutation in the zebrafish *rdh12* gene. After confirming that the zebrafish are carrying the genetic change, they are now breeding them to enable fish with the *rdh12* mutation to mate with each other and produce offspring with two defective copies of the gene in order to examine the clinical features.

**Pig.** Considering that the available *Rdh*12 knockout mice do not resemble the human pathology, researchers from China are proposing to generate a knockout or even a mutation-specific knockin minipig model. Both Dr. Ruifang Sui and Dr. Li Bin - who accepted to be interviewed in this study, agree that it would be very useful to test gene therapy in a pig model before going into humans. Dr. Ruifang Sui has experience in the generation and characterization of minipig models of ocular disorders like Bietti crystalline retinopathy and oculo-cutaneous albinism and she states that the advantage of the swine versus smaller animals is represented by higher similarity of the eye between humans and pigs, also in terms of size.

Another potential use of minipigs is to exploit them as a source of allogenic organs (i.e. eyes) by using pluripotent stem cells/blastocyst complementation strategy (Zhang et al., 2018). In the future, porcine-derived organs might be used as a xenogeneic source for transplantation in patients with end-stage organ failure. Enthusiasm in this direction was also paved by Zhang and collaborators who combined somatic cell nuclear transfer technology and an anophthalmic pig model (MITF^L247S/L247S^, Hai et al., 2017), obtaining allogenic RPE cells and a regenerated intact eye.

Other KOLs interviewed acknowledged that a minipig model would likely increase our understanding of the disease. However, opinions about feasibility in terms of costs and timing were divergent.

**2.2.2 Invertebrates**

**Drosophila melanogaster.** A study showed that Drosophila has six genes encoding short chain dehydrogenase/reductase (SDR) with ∼50% identity to human retinol dehydrogenase 12 (*RDH12*). Five are clustered on chromosome 2 and one is on chromosome X. Phylogenetically, Drosophila SDRs and human RDH12 have a common ancestry and similarities in the substrates and cofactor specificities suggest conservation of their function in retinoid metabolism (Belyaeva et al., 2009).

Even though a visual cycle was thought to be absent in Drosophila, it has been recently demonstrated that an enzymatic visual cycle exists in flies for chromophore regeneration and requires a photoreceptor retinol dehydrogenase, PDH (pigment cell enriched dehydrogenase), expressed in retinal pigment cells. Absence of PDH resulted in progressive light-dependent loss of rhodopsin and retinal degeneration, possibly because of toxicity associated with accumulation of retinaldehydes. These defects are partially rescued by ectopic expression of the mammalian RDH12 (Wang et al., 2010). The crystal structure of Drosophila PDH was recently determined and, based on it, the researchers analyzed mutations causing LCA13 in a homology model of human RDH12 to obtain insights into the molecular basis of *RDH12* disease-causing mutations (Hofmann et al., 2016).

The enzymatic activity relies on the dimerization of the protein as observed for other SDRs. A model of functionally related RDH12 allowed prediction of the impact of disease-causing mutations within the catalytic domain, including the dinucleotide-binding site of the protein or the dimerization interface.

**Caenorhabditis elegans.** Unlike Drosophila, C. elegans lacks opsins, utilizing a different mechanism for light detection (Edwards et al., 2008). Thus, nematodes have no need for production of retinoic acid or retinoid chromophores and, therefore, their RDH12 homologs probably possess different functions. However, nematode SDRs may represent the ancestral forms that gave rise to the family of enzymes capable of utilizing retinaldehyde.

**2.3 Are new RDH12 models worthy and feasible to develop?**

KOLs agreed that the lack of a reliable animal model limits the researchers’ ability to study RDH12 pathomechanism and is a major gap along the path from research to cure.

Although the translation of RDH12 gene therapy could probably be pursued even in the absence of proof-of-concepts from animals similar to the human disease, until effective in vitro and/or in vivo models are available, there will still be many unanswered scientific questions and untestable therapeutic approaches.

KOLs are optimistic about progress that is being made towards iPSCs development: this system would provide a meaningful platform for testing different strategies from gene therapy to compounds.

Concerning animal models, opinions are controversial. Some KOLs believe that animal models are not necessary to move on with a clinical trial since data gathered in vitro and from control animals would support approval from regulatory agencies. Others believe that new engineered mice or pig models could potentially pave the way to a better understanding of the disease, hence to better therapeutic interventions. Although feasibility would not be a problem, given the existence of several facilities around the world that could be contracted for model creation, costs and timing needed to build and study new disease models are a major issue.

**3. THERAPEUTIC PIPELINE**

**3.1 Introduction**

For most inherited eye diseases, including LCA/EOSRD and RP, no effective treatment exists. Management of most forms of LCA/EOSRD is symptomatic. Affected children benefit from correction of refractive error, use of low vision aids, when possible, and access to educational and work-related opportunities. However, the research field is very active and for a few genetic subtypes of LCA gene therapy has been recently developed and/or clinically tested (Russell et al., 2017).

Nonetheless, not all LCA patients can be treated with the same approach but specific therapies should be directed to each patient depending on the genetic background and the stage of retinal degeneration. For those who present an early stage of the disease, the aim would be to arrest and prevent vision loss, for example by gene augmentation or gene editing. For those with significant vision loss, but residual PRs at the time of presentation, a mix of gene augmentation and cell replacement would be useful. If the patient has already lost his/her vision due to PR cell death, gene augmentation will not be helpful. Improvement of visual function is potentially achievable only when evidence of dissociation of function and structure is present (Jacobson et al., 2005) that is, structural preservation, even in the presence of functional losses, is a prerequisite for certain types of therapies like gene therapy (Cideciyan and Jacobson, 2019). For LCA forms with no residual PR cell to be targeted, other kinds of therapies like regenerative medicine, optogenetics or a retinal prosthesis would be suitable.

An assessment of all the therapeutic approaches under investigation/development for IRDs and RDH12 was performed and is reported in the following paragraphs. A summary table of the therapeutic pipeline (**Table 5**), including the stage of development and the main barriers, can be found at the end of this chapter.

**3.2 Gene Therapy**

Gene therapy applied to retinal disorders aims at replacing the mutated gene and/or providing additional genes with neuroprotective or light-sensitive properties (optogenetics).

Gene replacement therapy, also known as gene augmentation therapy, is the most straightforward option for treating IRDs caused by a single recessive gene defect. It consists in the addition of a normal copy of the gene of interest into the affected cells to compensate for the loss of function due to the mutation/s. Gene therapy has been experimented in animal models of several retinal diseases ranging from achromatopsia and choroideremia to RP, LCA and Stargardt disease to mention a few. For the majority of these disorders clinical trials have been started and are underway (see table 1 in Dias et al., 2018). The most relevant example is represented by gene therapy for the treatment of *RPE65*-LCA, which has been recently approved and commercialized in the USA (2017) and Europe (2018) for adult and pediatric patients (voretigene neparvovec-rzyl/Luxturna by Spark Therapeutics). Luxturna is based upon an adeno-associated viral vector 2 (AAV2/2) delivering human *RPE65*. It is administered to patients with viable retinal cells via subretinal injection and known side effects are mainly associated with the surgical procedure itself, including eye redness, inflammation, pain and retinal detachment. The risk of compromising residual central vision in late-stage patients represents a roadblock for subretinal surgical manipulation, especially in those conditions where the retinal architecture is already fragile. Besides the risk associated to the surgical procedure, other limits of gene therapy are the need to treat at early stages of the disease, the degeneration of PRs despite gene correction and the questionable therapeutic longevity.

**Advantages of gene therapy for eye diseases are the immune-privilege status of the eye as well as accessibility and compartmentalization that reduces systemic unwanted effects and limits vector’s dosage.**

These points have emerged from the clinical trials conducted during the last decade on *RPE65*-LCA/RP patients. First, phase 1 and 2 trials launched by for-profit organizations (Spark NCT00516477; National Eye Institute/University of Pennsylvania NCT00481546; UCL/Targeted Genetics NCT00643747; Applied Genetic Technologies AGTC/Oregon Health and Science University/University of Massachusetts NCT00749957) demonstrated that AAV2 could be safely delivered to the retina despite variations in clinical trial conditions (Pierce et al., 2015). In 2012 a phase 3 clinical trial was started on 31 patients to examine the therapeutic efficacy of AAV2-*hRPE65*. The treated population showed a statistically significant improvement in the primary outcome, standardized multi-luminance mobility test (MLMT), and several secondary endpoints; moreover, stability up to the 1-year follow-up was confirmed (Russell et al., 2017). However, one challenge of gene therapy is the long-term sustained therapeutic effect of the vector: from the several RPE65 trials, a decline after initial improvement was noted and was related to the use of a cell specific RPE promoter. On the other hand, in other trials for *RPE65*-LCA, transgene stability was found when a ubiquitous promoter (CBA) was used (Jacobson et al., 2012; Cideciyan et al., 2013; Testa et al., 2013). This emphasizes the need to select optimal driver promoters on the basis of the vector type, target cells, undesirable off-target effects and desirable therapeutic outcomes. This point has been confirmed by other gene therapy trials like the one established to test safety and efficacy of a subretinal AAV2-h*MERKT* to treat patients with *MERKT*-driven RP. In this case as well, after early improvement there was a subsequent decline possibly due to the use of an RPE-specific promoter (VMD2) (Ghazi et al., 2016).

Besides the selection of the promoter, the choice of the vector is also important. It could be viral or non-viral. If the first is chosen, as in all ongoing clinical trials, the type of virus depends on the size of the gene to be delivered, the desired target cells, and the route of administration. Recombinant AAVs are the most frequently used vectors in retinal gene therapy, with a maximum packaging capacity of 4.7 kb. Different AAV serotypes have different cell tropism: almost all AAVs can transduce RPE cells, with AAV2/1, AAV2/4 and AAV2/6 being the most specific, whereas AAV2/5, 2/7, 2/8 and 2/9 can efficiently transduce PRs. The vector variant AAV7M8, which has been isolated by in vivo directed evolution, is capable of transducing the mouse outer retina after intravitreal administration and is currently undergoing further testing in other species (Ramachandran et al., 2017).

However, in some cases higher capacities are needed, hence lentiviral vectors, with a capacity of 8 kb, are used. For instance, gene therapy for Stargardt disease, caused by mutations in *ABCA4*, should be based on either lentiviral or dual AAV vectors because of the gene size exceeding the capacity of an AAV. Indeed, this disease is being treated in clinical trials using a lentiviral vector of Oxford BioMedica's Lentivector platform (NCT01736592 and NCT01367444), which has been shown to be safe and well tolerated in preclinical studies. Drawbacks of lentiviral vectors are potential insertional mutagenesis, complexity of production, and the large diameter (80–100 nm).

Given that *RDH12* cDNA is around 2 kb and is expressed in PRs, an AAV virus would be appropriate and the favoured serotypes would be AAV5, which has been shown to mediate efficient transduction of PRs in the murine and primate retina (Yang et al., 2002; Lotery et al., 2003) or AAV8, which has high tropism to PRs when administered in the subretinal space (Vandenberghe et al. 2011). Currently, AAV2 is the most common serotype used in ocular gene therapy, but AAV8 should be considered for those IRDs in which the primary damage is located to the PRs like *RDH12*-LCA. Another recent example of the use of AAV8 is represented by X-linked retinoschisis (XLRS) caused by mutations in *RS1*, a gene expressed in several retinal cell types, including PRs, ganglion cells, amacrine cells, Müller cells and bipolar cells. A phase 1/2 trial (NCT02317887) is evaluating the safety and tolerability of an intravitreal injection of an AAV8-*RS1*. In this case the choice of the intravitreal route is dependent on the need to treat the retina in its wholeness and to limit the risk of ruptures in an already fragile tissue.

Several other clinical trials targeting other genes mutated in RP, choroideremia and achromatopsia have been initiated and are registered in *clinicaltrials.gov* (**Table 5**). Most of them are in phase 2 and most of the therapies in development aim to restore expression of the mutated gene (*CNGA*, *CNGB*, *RPE65*, *RS1*, *RPGR*, *PDE6B*, *RLBP1*, *REP1*). Currently, AAV2-*REP1* for choroideremia is the only gene therapy in phase 3, the closest to potential approval. Phase 1/2 studies of *AAV2-REP1* in patients with choroideremia have produced mixed results. A small study from Nightstar Therapeutics suggests that it is possible to slow or stop the decline in vision and also to improve visual acuity in some patients but a study from Spark Therapeutics failed to demonstrate a meaningful benefit. Nightstar Therapeutics’ Phase 3 will complete global recruitment of 140 patients in the first months of 2020.

At the beginning of July 2019, a new publication from Dr. Thompson’s group came out on *Human Gene Therapy* describing the preclinical test of a gene therapy approach for *RDH12*-related degeneration (Feathers et al., 2019). Specifically, they have demonstrated that subretinal injection of rAAV2/5 carrying a human *RDH12* cDNA driven by a human rhodopsin-kinase promoter (rAAV2/5‐hGRK1p.hRDH12) exhibits stable and correctly localized transgene expression and is not toxic to the retina. In addition, this vector reconstitutes the reductase activity in the retinas of *Rdh12*KO mice and decreases their higher susceptibility to light-induced retinal damage. This strongly suggests that such a vector could be a potential therapeutic candidate for RDH12 patients, and it represents a significant advancement in the progress needed to move towards a Phase 1/2 clinical trial. Moreover, despite the presence of early and severe degeneration in RDH12 patients**, the researchers suggest that the best strategy would be to target those areas of preserved PRs in the peripheral retina that have been described** (Aleman et al., 2018), in an effort to decrease the rate of cell death and delay or prevent further loss of peripheral vision.

Other information gathered from KOLs’ interviews are as follows:

Jean Bennett and Tomas Aleman stated that they want to go into a gene therapy clinical trial for RDH12 within one year. They are setting up the proof-of-concept study in iPSCs and they are working to secure funds to do that. And, then, they will obtain the safety data that are needed to start the clinical trial.

Other gene therapy approaches that do not target gene replacement, include therapies for age-related macular degeneration (AMD), in which the gene therapy encodes a therapeutic molecule that inhibit angiogenesis or cell death, and vectors that deliver trophic factors or “optogenes” to restore vision. In the next paragraphs the latter approaches are discussed.

**3.3 Trophic factors**

In contrast to gene therapies, the advantages of neuroprotective strategies are that they do not depend on any specific mutation and may provide a longer time window for treatment.

A lot of preclinical studies have been performed using AAV vectors to carry genes related to the protection of PRs in several forms of RP. For instance, rAVV coding for the CNTF (ciliary neurotrophic factor) was tested on three rodent models of RP: morphological protection and preservation of PRs were obtained, but there was a suppression of the electrophysiological function (Liang et al., 2001). The delivery of anti-apoptotic (AAV-*XIAP*) or ER stress-relieving (AAV5-*BiP*) factors was also tested preclinically and gave positive results, but no clinical development has followed yet (Leonard et al., 2007 and Gorbatyuk et al., 2010). AAVs were also used to mediate the expression of two rod-derived cone viability factor (RdCVF) isoforms in two RP murine models with improvement of retinal cone function and delayed cone loss, after systemic and intravitreal administration (Byrne et al., 2015). These encouraging results has led Sparing Vision to develop RdCVF gene therapy. José-Alain Sahel has told us that a clinical trial for RdCVF is going to start by the end of 2020. Since RdCVF was shown to protect PRs from photo-oxidative damage (Elachouri et al., 2015) and considering the light-induced PR degeneration in *Rdh12*KO mice, RdCVF might be beneficial to *RDH12*-LCA patients.

In addition, neuroprotective strategies can act synergistically with gene replacement therapy. For example, the use of an AAV vector carrying the glial cell line-derived neurotrophic factor (GDNF) in combination with a vector carrying the causative genes of two types of RP led to significant improvements in two models of RP, suggesting that in the future a combination approach of gene replacement and neuroprotective factor therapy may provide enhanced rescue over either treatment alone (Dias et al., 2018).

**3.4 Optogenetics**

Optogenetics is the approach in which new genetic information encoding a light-sensitive protein is introduced into neurons with the purpose of stimulating them with light. Through this technique the novel light-sensitive protein converts secondary (bipolar cells) and tertiary (retinal ganglion cells) neurons into PRs or, more rarely, restores sensitivity to PRs themselves. The level of vision restoration possible through the current generation of optogenetics is limited (i.e. light detection/perception) and often requires additional hardware to achieve maximum benefit.

Two types of photosensitive proteins can be used: type 1 or microbial opsins (channelrhodopsins, halorhodopsins and archeorhodopsins), and type 2 animal opsins. While type 1 opsins are themselves ion channels or pumps, type 2 opsins exert their effect on cation channels via a G-protein coupled cascade. These optogenetic molecules are encoded by adeno-associated viral vectors (AAV) that deliver them to retinal neurons. Some AAV serotypes show tropism to particular neurons (AAV2, 5, 7-9) (Day et al., 2014) and they are delivered to the retina via two principal routes: subretinal and intravitreal. The light-sensitive proteins can be under the regulation of a ubiquitous promoter or under promoters that are either photoreceptor- bipolar cell- or ganglion cell-specific. Careful assessment of the status of the remaining retinal cells using high-resolution non-invasive imaging will inform the decision about which retinal cell type to select. Cone targeting is expected to produce optimal results, followed by targeting of bipolar cells and, lastly, ganglion cells. The outcome of the optogenetic therapy will depend on many factors, such as the degenerative state of the retina, the ability of the patient to learn the new retinal language driven by the “optogene”, and the rehabilitation program provided (Scholl et al., 2016).

Optogenetic approaches have yielded promising results in several pre-clinical models (Simunovic et al., 2019). Efficient expression of optogenes in cones and ganglion cells has been demonstrated in the retina of non-human primates *in vivo* and in post-mortem human retinas *in vitro*. A phase 1/2a trial sponsored by Allergan is currently underway in the USA, testing safety and tolerability of intravitreal RST-001 (AAV2-ChR 2) in patients with advanced RP (NCT02556736). A second clinical trial (NCT03326336), sponsored by GenSight Biologics, is evaluating safety and tolerability of GS030 in subjects with non-syndromic RP. GS030 is a combination of a gene therapy vector containing a photoactivatable channelrhodopsin protein (rAAV2.7m8-CAG-ChrimsonR-tdTomato) and a medical device consisting of visual interface stimulating glasses that amplify the external visual stimulus to the optogenetically engineered retina.

One of the main challenges of optogenetics is the poor sensitivity to light, that is high light intensity is required for activating channelrhodopsins. Moreover, unlike the retinal rod and cone PRs, channelrhodopsins cannot adapt their range or adjust their sensitivity. Another concern is the possibility of immune reaction to ectopically expressed proteins. However, compared to retinal prostheses, these strategies use a more naturalistic form of light stimulation and the clinical application can be less invasive. Optogenetics should be used at medium-advanced stages of retinal disease.

**3.5 Antisense oligonucleotides and gene editing**

Two novel nucleic-acid-based treatments that can be used especially in dominant forms of RP are: antisense oligonucleotides (AONs) and Crispr-Cas9 gene editing. Both of them are being tested in clinical trials in patients affected by LCA10.

The first approach is using QR-110, an RNA antisense oligonucleotide injected intravitreally in patients carrying the LCA10-causing IVS26 variant, which leads to aberrant splicing. Currently a randomized controlled multiple-dose phase 2/3 study (NCT03913143) is starting with the aim of evaluating efficacy, safety and tolerability of QR-110 after 2 years of treatment.

The second approach is based on Crispr-Cas9, which allows a one-time modification of the genome and leads to a precise and permanent intervention that can halt disease progression. This intervention, called AGN-151587/EDIT-101, consists of an AAV5 vector encoding SaCas9 with two gRNAs flanking the IVS26 mutation, and it has been recently translated into clinical development with a phase 1/2 trial (NCT03872479).

**3.6 Pharmacological approaches**

In the early stage of the disease, pharmacological treatments using neuroprotective substances may delay the progression of PR and RPE cell loss. Pharmacological treatment is used most often in the early stage, but it can also be used in all stages as an adjunctive treatment. However, there is no pharmacological therapy that has been undoubtedly proven to prevent the development and/or the progression of LCA and RP or to restore vision. Most pharmacological agents attempt to slow down disease progression through neuroprotection and to preserve useful vision of the affected individuals during their lifetime. Such strategies do not correct the underlying disease causes but aim at providing a supportive and conservative treatment.

Several classes of pharmacological agents and small molecules have been tested in retinal disorders so far:

- neurotrophic factors,

- anti-apoptotic agents,

- antioxidant agents,

- other drugs (zuretinol, …),

- primary amine-containing FDA-approved drugs (Maeda et al., 2011),

- combination of FDA-approved drugs acting on GPCRs (Maeda et al., 2016).

Neurotrophic factors are endogenously secreted small proteins with short half-lives. In the eye they are generally synthesized by Müller glia and RPE. Several neurotrophic and growth factors have been shown to be effective in protecting PRs in animal models. The most studied are: CNTF, BDNF, GDNF, bFGF, PEDF, LIF, NGF, RdCVF and LEDGF. Due to their short half-lives, strategies that promote their sustained release should be developed. Some examples are delivery through viral vectors or devices containing cells that secrete the recombinant protein of interest. Among the various trophic factors, only CNTF has been tested in large randomized trials (NCT00447980; NCT01530659; NCT00447993). However, intravitreal implant of an encapsulated cell technology releasing CNTF (NT-501; Neurotech) led to reversible loss of total visual field sensitivity in the short term and did not improve visual acuity, visual field sensitivity, or retinal structure over the long term in early and late-stage RP (Birch et al., 2016). J.A. Sahel has suggested that probably it was not the right factor, whereas T. Aleman and J. Bennett think that the experimentation was conducted on the wrong population of patients or using inappropriate outcome measures.

Based on the **assumption that PR degeneration mainly occurs through apoptosis in LCA and RP**, anti-apoptotic agents were also tested on several RP animal models and showed certain degrees of PR preservation. As for RDH12, increased apoptotic cell death was found in the retinas of *Rdh12*-deficient mice exposed to high intensity illumination (Maeda et al., 2006). If this type of cell death was confirmed to cause PR degeneration in human patients (i.e. iPSCs) as well, we could have a stronger rationale to use anti-apoptotic factors in LCA13.

Numerous are the active substances presently used to target apoptosis. However, patient use of anti-apoptotic molecules is rarely experimented, and no caspase inhibitor used for retinal disorders has reached phase 3 clinical trial despite the high number of molecules synthetized by drug companies. Some apoptotic agents are: tauroursodeoxycholic acid (TUDCA), rasagiline, norgestrel and myriocin (Dias et al., 2018).

TUDCA is a water-soluble bile salt that derives from the ancient Chinese pharmacopoeia; it acts by interfering with the upstream mitochondrial pathway of cell death, inhibiting caspase activation and oxygen-radical production and reducing endoplasmic reticulum (ER) stress. Tested in models of neural and retinal degeneration, TUDCA has been found to have protective effects in the eye, especially concerning retinal degenerative disorders (Boatright et al. 2009; Phillips et al. 2008). Clinical trials for ocular diseases are not reported (Vang et al., 2014).

The synthetic derivative of progesterone norgestrel, commonly used in hormonal contraception, showed neuroprotective effect in different animal models of RP (Doonan et al., 2011), likely through bFGF and LIF, one of the key survival factors in retinal cells under stress conditions. Nonetheless it has not been tested in humans yet.

Myriocin, a small-molecule inhibitor of serine palmitoyl-CoA transferase, is able to reduce ceramide, an important component of apoptosis signaling. It has been shown to rescue PRs from death when injected intraocularly in a mouse model of RP (Strettoi et al., 2010). Subsequent investigations made use of a non-invasive transcorneal drug delivery system based on eye drops of a suspension of solid lipid nanoparticles loaded with myriocin. This system is patented by Nanovector, has received ODD, and is suitable for pharmaceutical development, warranting further clinical evaluation (Platania et al., 2019). It has also been found that upon longer treatment cone morphology and function were well preserved even after major rod death. The authors speculate that myriocin can have a direct effect onto mechanisms that influence cone survival, maybe due to its immunosuppressant activity (through inflammation lowering).

Since oxidative damage has been implicated in PR degeneration (Campochiaro et al., 2015), antioxidants have been also suggested as potential therapeutic agents in RP and LCA. Among these are: N-acetylcysteine (NAC) and its amide (NACA), carcinine, vitamin A, docosahexaenoic acid (DHA) and lutein supplements. Oral and topical NAC, a glutathione precursor, and NACA have been shown to prevent retinal degeneration in preclinical studies of RP (Lee et al., 2011; Schimel et al., 2011). FIGHT-RP1 (NCT03063021), a phase 1 clinical trial of oral NAC in RP patients, was recently concluded, but the results are not known yet.

Carcinine (β-alanyl-L-histamine) is a natural imidazole-containing peptide derivative that has antioxidant properties and can scavenge reactive aldehydes produced by lipid peroxidation thus preventing these from reacting with cellular proteins. Anne Kasus-Jacobi and collaborators have shown that in the mouse retina RDH12 reduces 4-HNE to non-toxic alcohol, protecting PRs from light-induced apoptosis (Marchette et al., 2010). Moreover, by forming adducts with 4-HNE, carcinine administered orally or by intravitreal injection, strongly protects mouse retinae against light-induced PR degeneration (Marchette et al., 2012).

***However, when we contacted Anne Kasus-Jacobi she told us that carcinine is available in the US as an oral supplement, but it was never reviewed by the FDA for retinal dystrophies. Since she hasn’t a patent on it, it would be difficult for her to test and develop it further for this indication.***

The effect of vitamin A, DHA and lutein have been studied in large-scale, long-term randomized trials, but none of these studies definitively proved the efficacy of these factors, as the primary outcomes were not significant and only the analyses of either pooled data, subgroups, or secondary outcomes gave significant results (Dias et al., 2018). It should be noted that dietary supplementation of vitamin A can actually be deleterious and speed PR degeneration in some retinal disorders, like Stargardt disease.

There are contrasting data about oxidative stress in RDH12. Some groups reported accumulation of by-products of outer segment lipid peroxidation in the absence of RDH12 (Chrispell et al., 2009) and the ability of RDH12 to detoxify aldehydes and end-products of lipid peroxidation (Marchette et al., 2010). Others did not observe elevated lipid peroxidation products and other measures of oxidative stress in *Rdh12*KO mice (Kurth et al., 2006). Therefore, oxidative stress and its role in the pathogenesis of the disease should be further addressed (perhaps using novel in vivo and in vitro models).

This fact was confirmed by Jogin Desai who, during his interview, mentioned that several types of antioxidants have been tested so far, however no evidence of efficacy on preventing/slowing retinal degeneration has ever been gathered.

Other pharmacological treatments. In some forms of LCA/RP, impairment of rod PR function and subsequent degeneration of the retina are caused by a block of the visual cycle that leads to absence of 11-cis-RAL. Two examples are LRAT- and RPE65-related LCA. These forms of retinal degeneration can be treated by bypassing the biochemical defect through delivery of 9-cis-RAL. After successful tests in animal models (Van Hooser et al., 2000 and 2002), an open-label, proof-of-concept phase 1b clinical trial, in which 14 patients with RPE65- and LRAT-LCA were treated with 9-cis-retinyl acetate (QLT091001 or zuretinol), reported that this treatment was well tolerated and resulted in improved vision (Koenekoop et al., 2014). Most recently, a multicenter, open-label study (NCT01014052) in patients with RPE65- or LRAT-related RP confirmed safety and efficacy of a once-daily oral dose of QLT091001 for seven consecutive days (Scholl et al., 2015). This kind of compound is not applicable to RDH12-related degeneration which, differently from RPE65- and LRAT-related disorders, is not characterized by reduced chromophore levels.

Other pharmacological compounds shown to protect PRs in animal models of RP are: calcium channel blockers, calpain inhibitors, valproic acid, and isopropyl unoprostone. Some of them were tested in clinical trials as well.

Given the evidence of an increase of Ca2+ influx in PRs upon cell death, some calcium blockers were tested; on one hand D-cis diltiazem did not show any protective effects (Pearce-Kelling et al., 2001; Pawlyk et al., 2002), whereas nilvadipine was able to reduce disease progression in small clinical trials (Nakazawa et al., 2011 and 2013).

Valproic acid, a GABA-inhibitor originally used to treat epilepsy, gave controversial results regarding improvement of visual field and visual acuity in retrospective case series and small prospective studies (Bhalla et al., 2013; Kumar et al., 2014 Iraha et al., 2016; Sandberg et al., 2011; Shanmugam et al., 2012). Finally, isopropyl unoprostone, used as a topical treatment for glaucoma under the trade name Rescula, showed dose-dependent improvement in the sensitivity of the central retina in RP patients in a phase 2 trial (Yamamoto et al., 2012), but this beneficial effect was not confirmed in a subsequent phase 3 clinical trial (NCT01786395).

FDA-approved drugs containing primary amines have been shown to lower peak concentrations of all-trans RAL, without inhibiting chromophore regeneration in mouse models of retinal degeneration (Maeda et al., 2011). Through their amine groups, these drugs can form Schiff base adducts with all-trans-RAL, decreasing the formation and accumulation of toxic condensation byproducts like A2E and RALdi (retinal dimer), hence protecting against light-induced retinopathy. In particular, pre-treatment of *Rdh*12KO mice with one such compound (A20) prevented retinal degeneration induced by illumination at 10,000 lx for 60 min (Maeda et al., 2011). It is noteworthy that the selected compounds are primarily directed against infectious diseases, and they are less likely to target human proteins. Moreover, they have a good safety profile even at high doses. Krzysztof Palczewski thinks that these compounds should be tested in humans through a controlled clinical trial.

Another option suggested by Krzysztof Palczewski is represented by a cocktail of FDA-approved drugs comprehending agonists and antagonists of G protein-coupled receptors (GPCRs). Taking a systems pharmacology approach, Palczewski’s laboratory identified a group of compounds that exhibited synergistic activity protecting retinae from light-induced degeneration even when each drug was administered at a low dose (Chen et al., 2016). These drugs act by either stimulating GPCR signaling through the Gi/o family of G proteins or antagonizing GPCR signaling through Gq and Gs families of G proteins. One component of such cocktail is tamsulosin (TAM), an adrenergic receptor α1-antagonist approved for use in benign prostate enlargement. Other compounds are: the adrenergic receptor-α1 antagonist doxazosin (DOX), the β1-receptor antagonist metoprolol (MTP) and the synthetic ergot derivative bromocriptine (BRM). Preservation of both rod and cone photoreceptor structure and function was achieved using a combination of TAM+MTP+BRM or BRM+MTP+DOX administered by oral gavage or intraperitoneal injection. Bipolar and horizontal cells were also preserved by these combined treatments. The biological half-life of these drugs in human serum could enable once- or twice-daily oral administration for effective treatment of retinal degenerative disorders (Chen et al., 2016; Orban et al., 2018). Another possibility would be to formulate the mix as eye drops and test their efficacy experimentally to further minimize side effects. Even for these drug cocktails Krzysztof Palczewski has proposed to set up toxicology studies and a clinical trial.

Another pharmacological approach would be the use of translational readthrough inducing drugs (TRIDs) for those patients who carry nonsense variants resulting in premature termination codons (PTCs). TRIDS, like G418 and PTC124/ataluren, stimulate the bypass of the premature stop codon, allowing continuation of translation and restoration of a full-length protein. They have been tested in iPSC models of X-linked RP caused by a mutation in RP2 (Schwarz et al., 2015) and of MERTK-associated RP (Ramsden et al., 2017) with positive results. Similarly, Jogin Desai wants to test ataluren on PRs differentiated from an iPSC line of one RDH12 patient with a nonsense mutation.

PTC124 was also used in Usher syndrome type 1C where it was able to rescue translational readthrough of an USH1C mutation, restoring the functional properties of the protein (Goldmann et al., 2012). A clinical advantage of PTC124 over G418 is its use by oral administration or topical application. So far, PTC124 (PTC Therapeutics) has been tested in clinical trials – though without proven efficacy - on Duchenne muscular dystrophy, cystic fibrosis and hemophilia. Furthermore, it is not clear how to safely dose this drug at efficacious levels. The therapeutic outcome of TRIDs is influenced by the type of PTC, the genomic context of the nonsense mutation and the pathophysiology of the disorder.

**3.7 Cell therapies**

In IRDs, PR cells are susceptible to degeneration due to their malfunctions or to a primary dysfunction of the RPE; once lost, they cannot be endogenously regenerated in humans, and cell therapy to replace them is one of the promising strategies to restore vision. Depending on the nature of the primary defect and on the stage of the disease, PR, RPE cells, or both, might be transplanted to achieve therapeutic effects (M’Barek and Monville, 2019). The main sources of cells for transplantation are: RPE or retinal photoreceptor/precursors cells derived from fetal and adult tissues, embryonic stem cells (ESCs), induced pluripotent stem cells (iPSCs) or other stem cells, like bone marrow mesenchymal stem cells, iris pigment epithelium cells, umbilical cord stem cells, or retinal neurospheres.

**Being independent from the specific genetic defect, cell replacement therapies can be broadly applied to many IRDs.** However, some **concerns** are present:

- ethical problems for ESCs and fetus-derived cells,

- the risk of immune system rejection in case of allogeneic grafts and the need for immunosuppressive treatment,

- potential malignant transformation,

- difficulty of integration in the pre-existing retinal circuitry,

- limited source of the graft (especially from cadavers or autologous grafts),

- donor-to-donor variability for allogeneic transplants.

Human RPE cells from several sources have been derived and transplanted in various animal models of AMD and IRDs, like RP and Stargardt disease. In general, once derived, RPE cells can be injected in the subretinal or intravitreal space as a cell suspension as well as a monolayer. Prior to human testing, the quality of the cell formulations in terms of purity, safety, potency, and stability must be certified.

The first-in-man clinical trial with ESC-derived RPE cells (suspension formulation) was conducted in patients with dry AMD or Stargardt disease (Schwartz et al., 2012; Schwartz et al., 2015; Song et al., 2015). Even though the primary endpoint was safety, some improvements in visual acuity were reported. These effects were sustained up to four years in some patients.

The field of RPE transplantation is now moving in the direction of delivery of RPE-sheets on top of biological supporting scaffolds. Several clinical trials have been initiated with already promising first results (Mandai et al., 2017; da Cruz et al., 2018; Kashani et al., 2018). RPE cells derived from ESCs, autologous iPSCs or HLA-matched iPSCs, have been differentiated in vitro and then used for transplants. An early phase 1 clinical trial based on the treatment of RP and LCA by primary human RPE transplantation is currently underway in China (NCT03566147). These studies demonstrate the relative safety and practicality of stem cell-based therapies, even though it is too early to draw conclusions regarding efficacy. Transplantation strategies (e.g. using sheets or cell suspensions) may need further refinement or optimization to obtain substantial efficacy.

**For retinal disorders like RDH12-related degeneration**, in which patients carry a mutation in a PR gene, or for those carrying a mutation in an RPE gene and AMD patients at a stage where PRs are dead, **a transplant of immature PR cell suspension or RPE cells plus PR tissue is needed.** **The stage of development of grafted PRs is crucial for their correct integration.** Indeed, mature PRs will be less prone to establish the proper synaptic connections they require to be functional, whereas early postnatal retina has the best ability to integrate in the pre-existing circuitry. In the last few years a lot of protocols for the differentiation of ESCs and iPSCs into PRs have been developed (Maeda et al., 2019). PRs are generated in vitro through the formation of organoids resembling the optic vesicle or the optic cup stage and containing the neural retina and RPE cells. Graft sheets or enriched transplantable PRs are derived and transferred. The retinal sheet has an organized structure which includes unnecessary inner cells, whereas cell transplantation allows for direct contact between the host bipolar cells and graft PRs but in a disorganized structure. Some of the transplanted PRs, either in grafted retinal tissue or in cell suspensions, were reported to contact host bipolar dendrites, but in general, attempts at PR transplantation have led to few numbers of integrated cells. In one case an improved electrical response to light was detected after transplantation of suspended human ESC-derived PRs in a mouse model of LCA7 (Lamba et al., 2009). Jogin Desai, at Eyestem, is developing transplantable retinal PRs from iPSCs (EyecytePR) for the treatment of RP. He has already characterized them, tested for safety and purity, and started in vivo experimentation in a mouse model of RP. Even if preliminary data on in vivo survival have been reported, results are due in the next 6 months.

Recently it has been demonstrated that instead of, or in addition to, integration of donor cells into the host retina, photoreceptor precursors transplanted into animal models of retinal degenerative disease showed transfer of cytoplasmic material from donor to recipient PRs. Thus, transplanted donor cell integration and material transfer between transplanted and host cells may both underlie the therapeutic benefits associated with transplantation therapy.

Currently, around **20 clinical trials** involving cell-based therapy for degenerative retinal diseases are underway. However, the field of regenerative medicine is still at the beginning and attention should always be posed to safety issues. As a note of caution, there is evidence of severe visual loss, including complete blindness, after intravitreal injection of autologous adipose tissue-derived stem cells from unregulated clinics in the United States (Kuriyan et al., 2018; Duncan et al., 2018). The transformation of intravitreal stem cells into myofibroblasts was suspected to have caused proliferative retinopathy and retinal detachment in those cases.

| **Therapeutic approaches** | **LCA/RP/IRD status** | **Results/learning** | **Rdh12 status** | **RDH12 potential (High/low…)** | **Barriers** |
| --- | --- | --- | --- | --- | --- |
| **Gene Therapy** (gene replacement) | - Luxturna (RPE65): approved - RDH12: preclinical - GUCY2D: preclinical - LRAT: preclinical - CEP290: preclinical - MERKT: Phase 1 - RPGR: 3 Phase 1/2 - RLBP1 Phase 1/2 - PDE6B: Phase 1/2 - RS1: Phase 1/2 - Choroideremia (REP1): Phase 3  - Achromatopsia (CNGA3, CNGB3): Phase 1/2 - LHON (ND4): Phase 1/ 2 - Stargardt disease: Phase 2 - Usher syndrome type 1B: Phase 2 | - Early treatment for better outcome - Need of sufficient remaining target cells (PRs) - AAV2/8, the most efficient serotype to transduce PRs by subretinal injection - Selection of optimal driver promoters - Risks and invasiveness of subretinal injection - Questionable therapeutic longevity | - Preclinical: on RDH12KO mice (Feathers et al., 2019) | Medium/High depending on the stage of the disease and the spared retinal areas. | - Fast PRs degeneration: need of an early treatment - Long-term safety studies - Time and money for the proof-of-concept study - Time and money for the experimental and the approval processes - Rarity of the disease renders development of gene therapy costly and not commercially viable |
| **Other gene therapy approaches** | - AAV-RdCVF: preclinical. CT starting in 2020 for various forms of RP/LCA | - Potential intravitreal injection - It slowed the rate of cone cell death and increased the photopic ERG amplitude - It protects PRs from photo-oxidative damage | Potential enrollment in a CT in 2020 | Medium, because *RDH12*-lead degeneration is characterized by photo-oxidative damage | - Absence of a proper preclinical model on which to test it - Time to start the trial (end of 2020) - Inclusion criteria of the trial (still not known) - Number of residual cones |
| **Optogenetics** | - Restoration of light response in animal models -Two phase 1/2a CT underway: RST-001 and GS030 | - Poor sensitivity to lights - Potential immune reaction - Neural remodeling - Phototoxicity | - No preclinical test  - No clinical trial | It depends on the stage of the disease: need to assess the status of the remaining retinal cells | - Absence of a proper preclinical model - Evaluation of the retinal remodeling (reorganization of neurons?) - The rehabilitation program is critical to the success |
| **Gene editing and antisense oligonucleotides (AONs)** | - CEP290: QR-110: Phase 2/3 - CEP290: EDIT-101: Phase1/2 | - Vision improvement but questionable longevity. Need of frequent re-administration - Persistent expression of Cas9 in retinal cells, off-target cuts | - Idea | High for the few dominant forms of *RDH12*-related RP | - Time and money to develop a therapy directed to a very restricted population |

| **Pharmacology -** Trophic factors, anti-apoptotics, antioxidants and others | - NT501 (CNTF): Phase 3 - RdCVF: preclinical - TUDCA: preclinical - Myriocin: preclinical and development of a transcorneal delivery system - Norgestrel: preclinical - NAC: Phase 1 - Carcinine - Calcium Channel Blockers (CCB), Calpain Inhibitors (CI) and Valproic acid (VA): preclinical or small clinical trials/case series | - CNTF: no efficacy, but potential interest in the patented delivery system (ECT-NTC-200) - RdCVF: it protects against light-induced PR degeneration.  - TUDCA and Myriocin: potential clinical development - NAC: no results - Carcinine: preclinical efficacy against light-induced damage - CCB, CI and VA: variable results | - No preclinical test - No clinical trial | Variable, depending on the drug, with potential of reducing the progression (conservative effect) | - Short half-life of trophic factors: need for a sustained delivery system - Money and patients to develop a clinical trial - Potential need to test them in combination with a targeted therapy - Better understanding of apoptosis, oxidative stress and calcium signaling in RDH12-LCA - Need of an early treatment |
| --- | --- | --- | --- | --- | --- |
| **Pharmacology -** other small molecules | - 9-cis-retinyl acetate (QLT091001) for LRAT and RPE65: Phase 1 - FDA-approved drugs containing primary amines (Maeda et al, 2011) - FDA-approved cocktail of GPCR agonists and antagonists (Chen et al., 2016) | - QLT091001: safe and effective - Cocktails of FDA-approved drugs: good safety profiles and effective in preclinical tests | Cocktails of FDA-approved drugs tested on models of light-induced retinal degeneration | Medium: potential conservative effect | - QLT091001 not applicable due to lack of rationale - FDA-approved cocktails: money to develop toxicology studies and clinical trials;  - Need of an early treatment |
| **Pharmacology -**TRIDs | - G418, Ataluren/PT124: tested in vitro for nonsense RP-causing mutations | Positive results in terms of restoration of the full-length protein | In vitro testing by J. Desai | Medium, depending on the efficiency of the read-through | - For a limited subpopulation (few variants) - Need to test in RDH12-specific iPSCs - Type of PTC and genomic context of the mutation |
| **Cell therapies** | - ESC-derived RPE: Ph1/2 in Stargardt and AMD - Primary hRPE for LCA and RP: Ph1 - Human retinal progenitor cells for RP: Phase 2 (Jcyte) - ESC/iPSC-derived PRs: preclinical | - Need for refinement and optimization of the delivery procedures - Intravitreal injection can cause fibrosis and retinal traction | No pre-clinical test | Medium | - Ethical problems for hESCs and fetus-derived cells - Immune rejection risk and - immunosuppressant burden for allogeneic transplants - Risk of malignant transformation - Circuit integration problems - Donor to donor variability - Manufacturing costs |
| **Retinal prostheses** | 2 commercially available (Argus II and Alpha-IMS): Ph1/2 | - Modest and variable functional improvement - Only for end-stage patients - Limited visual fields - Maximum achievable resolution | Unknown | Low | - Further technological development to provide useful vision |

**4. CLINICAL ASSESSMENT**

**4.1 Diagnosis**

Diagnosis of LCA/EOSRD relies on clinical observation which shows poor visual function and a sluggish or near-absent pupillary response in early life. Visual acuity is severely decreased, and visual field is constricted at young ages. A characteristic finding is the Franceschetti's oculo-digital sign that involves eye poking, pressing, and rubbing. At fundoscopy, attenuation of retinal vessels along with variable signs of retinal degeneration are found. Macular atrophy and peripheral pigment accumulation are also present. Diagnosis is confirmed by photopic and scotopic ERGs close to or below the threshold. Normal ERG responses rule out a diagnosis of LCA. The molecular diagnosis may be performed using an arrayed primer extension (APEX) chip, which tests a subset of known mutations in LCA genes and next-generation sequencing (NGS). Mutations are confirmed by Sanger sequencing and segregation analysis in parents.

Prenatal diagnosis is offered by specialized laboratories for at-risk couples with identified disease-causing mutations.

For further information, check the Genetic Testing Registry (GTR): <https://www.ncbi.nlm.nih.gov/gtr/conditions/C2675186/>

**4.2 Clinical studies**

As of April 24^th,^ 2019, ClinicalTrials.gov does not report any clinical trial on RDH12, while **26 studies could be retrieved on LCA**. As for the trial status, 9 trials are completed, 8 are recruiting, 6 are active, not recruiting, and 3 not yet recruiting. 8 out of 26 studies are observational and 18 interventional.

Among the observational studies, there are:

- Retrospective (NCT02970266, NCT02575430) or prospective (NCT02714816, NCT03396042) studies about the natural history of visual function in patients with IRD diagnosed as LCA or RP caused by mutations of RPE65, LRAT and CEP290 or other genes;

- The FFB disease registry addressing several inherited retinal degenerative diseases (<https://www.myretinatracker.org/>; NCT02435940);

- Long-term follow-ups of patients treated with the adeno-associated virus (AAV) coding human RPE65 known as Voretigene neparvovec/Luxturna (NCT02946879).

**The interventional trials are mainly safety and efficacy studies for LCA2 patients treated with Luxturna**. There are also two interventional studies that evaluate safety and efficacy of a **splice-modulating oligonucleotide** (QR-110; NCT03140969) and a **gene editing product** (AGN-151587/EDIT-101; NCT03872479) to eliminate a specific mutation in CEP290 (LCA10). Preliminary results of NCT03140969 support an acceptable safety profile and suggest a potential improvement of visual acuity and light sensitivity (Cideciyan et al., 2019).

Other trials investigated oral administration of a 9-cis-retinyl acetate in RPE65- and LRAT-LCAs. A Chinese phase 1 study, sponsored by Eyecure Therapeutics Inc., evaluates the safety of primary RPE subretinal transplantation in patients suffering from LCA or RP (NCT03566147). Finally, a new trial testing safety and tolerability of subretinal injections of SAR439483 (Sanofi) is going to start in July 2019 in patients with GUCY2D-LCA (NCT03920007).

The majority of these interventional trials are open-label and can be randomized or not.

**4.3 Clinical endpoints**

The clinical endpoints and related outcome measures that are in the clinical trials mentioned above, are listed in Table 4. Some of these are likely to be used also for RDH12-lead LCA future clinical trials.

| **Endpoint** | **Outcome measures** |
| --- | --- |
| Visual function  Visual fields (static and kinetic) | Humphrey computerized testing, static perimetry, microperimetry, Goldmann kinetic perimetry, visual field area.  Vision-guided behavior |
| Visual acuity | Measurement of the ability to read letters on a standardized chart from a specific distance (Early Treatment Diabetic Retinopathy Study ETDRS) |
| Pupillometry (Pupillary light reflex) | Objective physiological testing: latency of pupil movement, amplitude of pupil constriction |
| Light sensitivity | Full-field light sensitivity threshold (FST) testing (dark- and light-adapted conditions with chromatic stimuli to capture rod and cone function) |
| Contrast sensitivity | Lea symbols/Pelli-Robson chart |
| Retinal structure | - Optical coherence tomography (OCT): high-resolution cross-sectional images of the retinal architecture.  - Fundal autofluorescence, color fundus photography |
| Retinal function | Electroretinogram (ERG) |
| Functional endpoint | Standardized multi-luminance mobility test (MLMT)* |
| Visual function and Quality of Life (QoL) | National Eye Institute Visual Function Questionnaire-25 (NEI-VFQ-25) and QoL questionnaire |

**Table 4.** Outcome measures. *MLMT is a standardized, lab-based test that simulates everyday walking environments. Participants were observed while navigating a course with obstacles of varying height under different levels of illumination. The MLMT has demonstrated construct and content validity and was specifically designed to measure the impact of LUXTURNA on activities of daily life.

According to Hendrik Scholl, MLMT would not work in an RDH12 trial whereas Full-field Stimulus Testing (FST) that was used as a secondary outcome measure in the RPE65 trials, can provide an early efficacy readout. From his point of view, a major obstacle with MLMT is that the test is currently available only in Philadelphia and Iowa and it involves significant effort and costs. For a registration trial, preliminary discussions with the FDA and EMA would be required; the regulators may need specific mobility tests or real-life outcome measures to show clinical utility.

According to J.A. Sahel, the primary outcomes that are likely to be used, are FST and mobility test that work better than other tests based on what was learned from RPE65 trials. He believes that there can be additional valuable measures such as visual acuity, central vision, dark-adaptation, OCT, and autofluorescence imaging. In addition, he is working on patient-reported outcomes (PROs) with the Foundation Fighting Blindness and believes that PROs are very useful, though they cannot replace objective tests such as mobility test.

**4.4 Medical devices**

There are no specific medical devices for people affected by *RDH12*-associated LCA or LCA. However, subjects with impaired vision can take advantage of various types of devises that can be classified in three main categories:

1. Cortical prostheses
2. Optic nerve prostheses
3. Retinal prostheses

Cortical prostheses

Cortical prostheses are neuro-prosthetics made up of an implantable multielectrode array that directly stimulates the visual cortex. They could be used when visual pathways are not preserved. Positioned on the surface of the visual cortex, the first models provided poor spatial resolution and could cause discomfort or damage to the surrounding tissues and focal epileptic activity. Instead, the most developed Utah Electrode Array (UEA) uses deeper cortical stimulation that, in principle, can provide higher resolution images needing less power and causing less damage.

Electrical stimulation of the primary visual cortex is one possible scenario that is currently in clinical trials. An early feasibility study is evaluating the safety of the visual cortical prosthesis Orion Visual Cortical Prosthesis System and surgery, as well as the reliability of the system and the usefulness of any restored vision (NCT03344848).

Optic nerve prostheses

Optic nerve prostheses are used for blind persons with functional retinal ganglion cells (RGCs). They consist of microelectrodes inserted onto the optic nerve. These are connected to a stimulator implanted in a small depression in the skull. The stimulator receives signals from an externally worn camera that are translated into electrical signals directly stimulating the optic nerve.

Prostheses that target the optic nerve and the visual cortex are under development.

Retinal prostheses

Retinal prostheses are implantable devices that replace phototransduction in the eyes of individuals with retinal diseases like RP, LCA and macular degeneration. They receive and process incoming light, transmit the information in the form of electrical impulses to the inner retinal layers that are spared by the degenerative process, deliver stimuli to axons of RGCs within the optic nerve and evoke a response in the visual cortex to induce phosphenes. The energy required by these devices can be provided by an external power source or by incident light acting through photoelectric cells.

Retinal prostheses can be surgically implanted at advanced stages of disease when degeneration has proceeded, and the patient retains just light perception or is totally and bilaterally blind. However, RGCs and the visual pathways need to be viable for these devices to function.

Retinal prostheses can be of two types: 1) microelectrode and photovoltaic devices based on inorganic technologies and 2) new generation-prosthetics based on organic molecules, such as organic photo-switches and conjugated polymers.

**1) Inorganic retina prostheses**

Electrical impulse delivery is accomplished by micro-photodiode arrays or micro-electrode arrays. Retinal prostheses are categorized based on where they are implanted in the eye and how they deliver the electrical stimulus to the RGCs. Electrical array chips can be placed in the epiretinal, subretinal or suprachoroidal position.

**Epiretinal devices** adjoin the RGC layer and directly stimulate the final common output from the retina. This system has the advantages to be easier and less risky to implant, has a long-term stability and allows for heat dissipation. Nonetheless, it eliminates the use of eye movements, requiring an external camera coupled with an image processing unit (VPU), and bypasses the residual intraretinal processing system with limited recreation of the physiological retinal topographic organization. Examples of epiretinal devices are:

- Argus II Retinal Prosthesis System (Second Sight Medical Products)

- EPI-RET3 (EPI-RET Project)

- IRIS V2 (Pixium Vision)

Argus II and IRIS V2 are both electrode-based devices characterized by relatively low resolution of the chips. Higher resolution prototypes have also been proposed (Ahnood et al., 2017).

**Subretinal prostheses** consist of a microphotodiode array placed behind the retina, between the sclera and the bipolar cells. The incident light is transformed into electrical potentials that excite bipolar cells to form an image sensation. Subretinal implants may benefit by utilizing intact middle retinal layer processing pathways formed by amacrine, horizontal, and bipolar cells. Moreover, they preserve the natural eye-head coordination in vision. However, retinal remodeling and gliosis render this approach more challenging in the advanced stages of disease. Other disadvantages are: long surgery times, potential thermal injury and limited size of the device. Examples of subretinal devices are:

- Retina Implant Alpha IMS (Retina Implant AG, which has discontinued its business activities at the beginning of 2019);

- Subretinal Retinal Prosthesis (Boston Retinal Implant Project);

- Photovoltaic Retinal Implant (Prima; Pixium).

Alpha and Prima are active chips in which a large array of photodiode/electrode units converts light into an electrical stimulation of the external retina. Alpha is sensitive to ambient light but needs wiring for power supply. On the other hand, Prima is fed wirelessly with an infrared beam that powers and sends the visual information to the photovoltaic chip.

**Suprachoroidal prostheses** are placed between the posterior blood supply of the eye (choroid) and the outer white layer of the eye (sclera), with this surgical location primarily being chosen for stability and safety. Since it does not need transvitreal surgery, it is potentially less invasive and more easily accessible for repair or replacement. However, the suprachoroidal space is highly vascularized and there is a significant risk of hemorrhage and post-implantation fibrosis. Furthermore, due to its distance from the neurosensory retina, this design requires greater stimulation power to elicit visual percepts. Examples of suprachoroidal prostheses are:

- Suprachoroidal Retinal Prosthesis by Bionic Vision Australia

- Suprachoroidal-transretinal stimulation (STS) system (Japan’s Artificial Vision Project and NIDEK).

Implants of the first retinal prostheses began in 2002 with phase I clinical trials for the Argus I. In 2011, the next generation Argus II received marketing authorization in Europe after successful implantation in 30 patients (Phase II multicenter trial NCT00407602). Safety and efficacy on functional visual and real-world task performance were evaluated and overall the subjects performed better with the device on than off (Humayun et al., 2009; Dorn et al., 2013; Ahuja et al., 2011). The 10-year follow-up study will be completed in 2019. In 2013, Argus II became the first retinal prosthetic device to be approved by the FDA for late-stage RP. Currently, it is the most used retinal prosthesis worldwide with over 250 patients implanted to date. However, NICE assessment of the Argus II required more data to ascertain patient benefit in terms of quality of life.

The IRIS V2 obtained CE approval in 2016 and early results were promising. Pixium has postponed the trial for further refinement of the device and surgical method due to a lifespan of the device shorter than expected.

The Alpha IMS subretinal device revealed an acceptable safety profile and significant improvement in terms of functional outcome measures (NCT01024803). Moreover, the next version, Alpha AMS, received CE approval in 2016 and showed similar safety and functional benefits. However, Hendrik Scholl informed us that the German company, Retina Implant AG, that developed and marketed the retinal prostheses in Europe, discontinued this business in March 2019. This was due to the innovation-hostile climate of Europe's rigid regulatory and health systems and the fact that, despite a positive feedback from the patients, the results ultimately did not meet the expectations.

As for Prima, 5 patients with dry AMD have been implanted in France with 5 more planned in the US, to assess safety and performance over 36 months. Preliminary results are anticipated this year.

Several groups in the world are currently working on retinal prostheses. As mentioned above, some studies are at the stage of human testing while other have already obtained FDA/EMA approvals.

**2) Organic retinal prostheses**

Common problems posed by inorganic-based devices are: 1) limited biocompatibility of the rigid chips, 2) inability to cover large retinal areas (narrow “tunnel vision”) and 3) heat production. Organic electronic devices based on combinations of semi conductive and conductive polymers, can overcome these problems. Some examples are: laser micro-structured diamond electrode arrays (Prawer et al., 2018), liquid crystal polymers (Jeong et al., 2013), or a composition of a passive substrate (silk fibroin or polyethylenterephthalate), an organic conductive layer of PEDOT:PSS and a light-sensitive semi conductive layer of P3HT (Ghezzi et al., 2011 and 2013). This latter device proved safe and able to rescue visual functions (evaluated as pupillary reflex, visually driven behavior, visually evoked potentials and visual acuity) in a rat model of RP (Maya-Vetencourt et al., 2017)

Organic retinal prostheses have several convenient features:

- they are tissue-friendly because of their conformability and intrinsic biocompatibility;

- they are fabricated with straightforward and inexpensive processes;

- they are ultrathin, lightweight and deformable;

- they have greater longevity and chemical stability than inorganic counterparts;

- they don’t need a power supply or an external camera;

- they can potentially cover large retina areas.

Further development and experimentation in clinical studies are foreseen (Bloch et al., 2019).

**4.5 Patient registry and biobank**

No RDH12 patient registry has been established so far although all KOLs interviewed in this study agreed that it would be paramount to further research and knowledge. In fact, being RDH12 a very rare disease, patients’ data collected by single research and clinical institutions led to the publication of a few natural history studies.

In 2018, Zou et al. published the results from a phenotypic study performed in a patient cohort of 38 subjects. Genetic analyses including next generation sequencing (NGS), Sanger and segregation analysis, were performed and subjects underwent full ophthalmic examination. Twenty-five different mutations were found (the vast majority of these were missense) plus 6 new variants. Another natural history study published the same year (Aleman et. al, 2018), described the phenotype of 21 subjects from 14 different families and concluded that RDH12-associated degeneration of the retina is an early-onset disease affecting predominantly the central part of the retina, with a less severe impact on rods. More recently Fahim et al. (2019) described RDH12 disease natural history through a retrospective study conducted on 57 patients. The authors confirmed that RDH12-induced degeneration has an early onset with all affected individuals presenting macular atrophy, significantly reduced if not absent ERG, loss of retinal structure/function within the age of 10.

However useful and informative, these single-center studies are usually limited in numbers and data collected cannot not be shared widely nor made available to external investigators.

Therefore, the availability of an RDH12 registry, pooling data from multiple sources/centers, would enable the comprehensive collection of patient data providing a resource for prospective natural history studies and for future clinical trials.

**Patient registries are important tools in a strategy targeted at accelerating research into rare diseases and development of new drugs.** According to the current definition a patient registry is an *organized system that uses observational study methods to collect uniform data (clinical and other) to evaluate specified outcomes for a population defined by a particular disease, condition, or exposure, and that serves one or more predetermined scientific, clinical, or policy purposes*.

A registry can have several different objectives (however, it is recommended to define/select a few at start):

- pooling data for epidemiological analyses;
- recruiting patients for clinical trials or observational studies;
- learning about the natural history of the disease;
- developing research hypotheses;
- improving and monitoring the quality of care;
- monitoring outcomes and studying best practices in care or treatments;
- serving as a recruitment tool for the launch of studies focusing on disease aetiology, pathogenesis, diagnosis or therapy;
- including biological specimens or links to specimen data.

Data included in patient registries may come from a variety of sources, including electronic medical records, clinician- or patient reported clinical outcomes, diagnostic reports or images, hospital records, collected/donated blood or tissue samples, or questionnaires/surveys completed by clinicians or patients (or both).

Essentially, registries can be of two type:

1) registries that are generated by researchers or institutions to collect data that can be used for a specific research agenda;

2) registries that are generated and powered by patients and their organizations that can be similar in several respects to researcher-generated patient registries in terms of objectives and features, with one exception: in patient-powered patient registries, patients/families or patient organizations “power” the registry by managing or controlling the collection of the data and the research agenda for the data, and by participating to the translation and dissemination of the results obtained from the data.

**In recent years, due to the growing need to** **more directly focus research on patients’ needs, organizations have created and operated “patient-powered” registries. Usually, these registries are managed by patients through a disease advocacy organization receiving guidance and input from a scientific board of advisors.**

This is the case of My Retina Tracker^^[[5]](#footnote-6)^^ a global patient registry for IRDs established in 2014 by the Foundation Fighting Blindness. The overall aim of My Retina Tracker is to provide pre-screened researchers and companies with de-identified patient and disease data for relevant studies, including IRD clinical trials and natural history studies. The registry is patient controlled; each patient uploads and maintains his/her own record. However, for reasons related to its design, this registry does not presently allow longitudinal studies.

It is shared among stakeholders that, in addition to a patient registry, the availability of a global accessible **biobank** collecting patients’ biological samples and/or a repository of RDH12 pre-clinical models would provide investigators with tremendous resources for basic as well as pre-clinical research.

A biobank is a biorepository that collects, stores and distributes biospecimens and associated data for use in research. The data associated with stored biospecimens have increased in complexity from basics data (i.e. date of collection and diagnosis) to information encompassing many features of participant or patient phenotype. Interestingly, virtual biobanks are also developed to help investigators locate biospecimens for testing and data mining. Such virtual biobanks can be accessed through software or web portals devised to connect biobanks and investigators around the world.

In general, **optimum collection, processing, storage, tracking and shipment of biospecimens are key to an effective biobank**. Therefore, the creation of such infrastructures in the RDH12 field would require that a number of factors (including availability of sustained funding and expert personnel) be taken into consideration. These need to be addressed altogether in order to achieve high quality biospecimens with well-characterized data and ensure that best practices are applied.

**5. KEY MESSAGES AND GAPS IDENTIFIED**

An overview of the key messages from this study is provided below. In the context of a refinement study to develop a strategic plan focused on the most feasible and promising actions to undertake, such information could provide a platform for discussion with a plenary scientific advisory board involving experts in relevant fields.

1. With the exception of RPE65, LCA remains untreatable. However, high interest of the eye research community towards the development of potential therapies for LCA as well as RDH12-LCA is noticed;

2. Mouse models developed so far do not recapitulate the human RDH12 phenotype. Therefore, better models are needed to increase our knowledge of disease mechanisms and to enable clinical translation;

3. iPSCs and organoids would be extremely valuable for pre-clinical testing of new therapeutic approaches. Efforts are being made in this direction from different labs around the world and there is growing expectation that new cellular models will soon be available for drug screenings/tests;

4. In terms of therapy developments, gene therapy approaches are the most promising and advanced ones, with a proof-of-concept study recently published from Debra Thompson’s lab. Other investigators are actively pursuing this avenue; a clinical trial may be launched in one year by the group of Jean Bennet. The field sees high competition in this respect;

5. Cell therapies would be worth pursuing in order to address the potential pitfall due to photoreceptors degeneration; however, research in this direction is still far from translation and several concerns need to be addressed including: ethical issues for ESCs and fetus-derived cells, risk of immune system rejection in case of allogeneic grafts and consequent need for immunosuppressive treatment, potential malignant transformation, difficulty of integration in the pre-existing retinal circuitry, etc.;

6. Several different pharmacological approaches to slow down PR degeneration target common pathways among IRDs and those include: neurotrophic factors, anti-apoptotic agents, antioxidant agents, and others. However, there is no pharmacological therapy that has been undoubtedly proven to prevent the development and/or the progression of LCA and RP or to restore vision. An ad hoc study on pharmacological interventions involving expert pharmacologists as well as physicians/ophthalmologists could be carried out;

7. An RDH12 patient registry as well as biobanks and/or other accessible biorepositories for cell and animal models, would be crucial to foster research, increase knowledge of the disease and accelerate clinical translation. However, setting up such infrastructures requires that a number of factors be addressed in order to ensure high quality data/biospecimens availability for use in research.

8. The RDH12 field sees several outstanding research teams actively involved. Therefore, there is a real opportunity for more collaboration and data sharing that will surely speed up research for the benefit of the patients.

Pre-clinical research

- - Lack of functional in vivo models that resemble the human phenotype, for pre-clinical in vivo testing of therapeutic approaches
  - Lack of biobanks and other accessible repositories of cell and animal models that meet the demand for high quality biospecimens

Clinical knowledge of disease

- - Lack of a disease-specific registry for prospective natural history studies and selection of patient cohorts in future clinical studies
  - Missed diagnoses and uncertain epidemiology

Clinical trials

- - Lack of funds for clinical translation of therapeutic approaches
  - Small patient population, need of multi-center clinical trials
  - Lack of collaboration and data sharing
  - Identification of optimal outcome measures according to the type of intervention

Far from being all-encompassing, the diagram below shows some of the main gaps identified along the path-to-cure that pertain to research infrastructures and dynamics:

**APPENDIX A**

**RDH12 known mutations (Sept. 2019)**

| **DNA** | **Protein** | **Exon** | **Features** | **Zigosity** | **Disease** | **References** | **Frequency** |
| --- | --- | --- | --- | --- | --- | --- | --- |
| c.2T> C | M1? | 1 | First codon. | Compound heterozygous with G145E |  | Thompson et al., 2005; Valverde et al., 2009 |  |
| c.38 C>A | Ser13X | 1 | Expected to result in NMD and null phenotype, but 45% RDH12mRNA in blood cells. A downstream methionine at position 17 with codon ATG may serve as an alternative translation start site and account for the relatively mild phenotype in this individual. | Homozygous | early-onset | Fahim et al., 2019 |  |
| c.57_60delTCCA | p.Ala19Alafs | 1 | Frameshift. |  | LCA | Walia et al., 2010; Fahim et al., 2019; Carver lab. |  |
| c.57_60del | p.P20del | 1 |  | Compound heterozygous with c.506G>A p. R169Q |  | McKay et al., 2011 |  |
|  | p.lIe22Gly | 1 | MS | Comp hz with Leu214Pro |  | Aleman et al., 2018. |  |
| c.133 A>G | p.Thr45Ala | 2 | MS. Predicted prob damaging (polyphen), damaging (SIFT), deleterius (provean) | Comp hz with Ala269del |  | Aleman et al., 2018; Fahim et al., 2019 |  |
|  | p.G46G | 2 | On first glance appears to be a silent mutation, can lead to an altered splice-acceptor site and the production of a truncated protein. | heterozygous | LCA | Sun et al., 2007 |  |
| c.146C>T | p.T49M | 2 | Transition. Increased degradation via the proteasome. Aberrant/reduced enzymatic activity in COS-7 cells. It interferes with the binding of NADP as aa49 is located in the P-binding loop and the larger hydrophobic side chain of Met could perturb co-factor binding. Reduced affinity for NADPH and increased proteosomal degradation. Reduction of RDH activity. Predicted prob damaging (polyphen), deleterius (provean),damaging (SIFT). | Homozygous or Compound heterozygous with p.R62X or p.A269fsX270 | Juvenile RP (cHz); LCA | Janecke et al., 2004; Sun et al., 2007; Valverde et al., 2009; Lee et al., 2010; McKay et al., 2011; Zou et al., 2018; Aleman et al., 2018; Carver lab. | 9.2% in the Chinese population |
| c.146C>A | p.T49K | 2 |  |  |  | McKay et al., 2011; |  |
| c.146 C>A | p.Thr49Lys | 2 | Predicted poss damaging (polyphen), deleterius (provean), damaging (SIFT). |  |  | Fahim et al., 2019. |  |
| c.152T>A | p.I51N | 2 | Increased degradation via the proteasome. | Compound heterozygous with p.Arg62X |  | Lee et al., 2010. |  |
| c.178 G>C | p.Ala60Pro | 2 | Ala60 may be liable of cone dysfunction. |  | cone dystrophy | Abu-Safieh et al., 2013 |  |
| c.178G>A | p.Ala60Thr | 2 | Predicted prob damaging (polyphen), deleterius (provean), damaging (SIFT). Ala60 maybe liable of cone dysfunction. | Comp hz with p.Val146Asp or p.Ala47Thr | CORD | Zou et al., 2018; Fahim et al., 2019. |  |
| c.184C<T | p.R62X | 2 | Transition. Null, nonsense mutation. Italian-Greek ancestry. | Compound heterozygous with p.T49M or I51N or p.A269AfsX1 or Leu99Ile |  | Janecke et al., 2004; Perrault et al., 2004; McKay et al., 2011; Aleman et al., 2018; Zou et al., 2018; Fahim et al., 2019. | 14.5% in the Chinese population. |
| c.185 G>T | p.Arg62Leu | 2 | Predicted prob damaging (polyphen), deleterius (provean), tolerant (SIFT). |  |  | Fahim et al., 2019. |  |
| c.139G>A | p.A47T | 2 | Dramatically reduced ability to convert all-trans RAL to all-trans ROL (5-18% wt). Predicted prob damaging (polyphen), deleterius (provean), damaging (SIFT). | Comp hz with p.Ala60Thr | LCA/EOSRD | Thompson et al., 2005; Zou et al., 2018; Fahim et al., 2019; Carver lab. |  |
| c.164C>T | p.T55M | 2 | Dramatically reduced ability to convert all-trans RAL to all-trans ROL (5-18% wt). | Comp hz with R295X | LCA/EOSRD | Thompson et al., 2005; Walia et al., 2010; Carver lab. |  |
| c.164C>A | p.T55K | 2 | Transversion. Predicted as probably damaging by both SIFT (0) and PolyPhen-2 (1). | Compound hz with p.H179D | LCA | Li et al., 2017 |  |
| c.99_102dupAAAT | p.V35fsX62 | 2 |  | Compound heterozygous with L99I |  | Thompson et al., 2005; Valverde et al., 2009 |  |
| c.226G>A | p.G76R | 3 | MS. Predicted prob damaging (polyphen), deleterius (provean), damaging (SIFT). | Homozygous or compound heterozygous with S175L or Ala126Val | LCA | Aldahmesh et al., 2009; Wang et al., 2015; Aleman et al., 2018; Fahim et al., 2019. |  |
| c.250C>T | p.R84X | 3 |  | Comp hz with p.G127GfsX1 |  | McKay et al., 2011 |  |
| c.295C>A | p.L99I | 3 | Dramatically reduced ability to convert all-trans RAL to all-trans ROL (5-18% wt). Predicted intolerant (SIFT), probably damaging (polyphen2), neutral (pMut), deleterius (provean) | Homozygous or compound hz with p.Gly127X; A269fsX270 or Arg62Stop, A47T or p.L149P | LCA/EOSRD or arRP | Perrault et al., 2004; Thompson et al., 2005; Valverde et al., 2009; Walia et al., 2010; MacKay et al., 2011; Chacon-Camacho et al., 2013; Aleman et al., 2018; Fahim et al., 2019; Carver lab |  |
| c.193C>T | p.R65X | 3 | Nonsense. | Homozygous or Compound hz with V146D or p.A269GfsXI | LCA | Thompson et al., 2005; Schuster et al., 2007; McKay et al., 2011; Wang et al., 2015; Zou et al., 2018; Fahim et al., 2019. |  |
| c.209G>A | p.C70Y | 3 | Predicted intolerant (SIFT) and pathological (pMUT), probably damaging (Polyphen2), deleterius (provean). | Comp hz with p.A269AfsX1 |  | McKay et al., 2011; Fahim et al., 2019. |  |
| c.302 A>G | p.Asp101Gly | 3 | MS. Predicted prob damaging (polyphen), neutral (provean), damaging (SIFT). | Comp hz with Arg295Stop |  | Aleman et al., 2018; Fahim et al, 2019. |  |
| c.316 C>T | p.R106X | 3 |  | Comp. hz with p.A269AfsX1 |  | McKay et al., 2011; Fahim et al., 2019. |  |
| c.325 G>C | p.Ala109Pro |  | MS. Predicted prob damaging (polyphen), damaging (SIFT), neutral (provean) | Comp hz with Tyr226Cys |  | Moosaje (unpublished); Fahim et al., 2019. |  |
| c.375T>A | p.N125K | 4 |  |  |  | Thompson et al., 2005 |  |
| c.377 C>A | p.Ala126Glu | 4 | MS. German ancestry. Predicted prob damaging (polyphen), deleterius (provean), damaging (SIFT). | Comp hz with Ala269del | LCA | Aleman et al., 2018; Fahim et al, 2019; Carver lab. |  |
| c.377 C>T | p.Ala126Val | 4 | MSPredicted prob damaging (polyphen), deleterius (provean), damaging (SIFT). | Comp hz with Gly76Arg | LCA | Aleman et al., 2018; Fahim et al, 2019; Carver lab. |  |
| c.381_delA | p.G127GfsX1 | 4 |  | Comp hz with c.250C>T, p.R84X |  | McKay et al., 2011 |  |
| c.379G>T | p.G127X | 4 | Nonsense mutation. | Homozygous or compound hz with p.Leu99Ile or p.R84X | LCA/EOSRD | Perrault et al., 2004; Thompson et al., 2005; Schuster et al., 2007; McKay et al., 2011; Fahim et al., 2019. |  |
| c.383 T>G | p.Val128Gly |  | Predicted prob damaging (polyphen), deleterius (provean), damaging (SIFT). |  |  | Fahim et al., 2019. |  |
| c.400 T>C | p.Ser134Pro | 4 | MS. Predicted prob damaging (polyphen), deleterius (provean), tolerant (SIFT). | Comp hz with Val233Leu |  | Aleman et al., 2018; Fahim et al, 2019. |  |
| c.437T>A | p.V146D | 4 | NAD(P)-binding domain of RDH12. The substituted amino acid is predicted to alter the hydrophobicity of RDH12 protein. Probably to be damaging to protein function (PolyPhen2 scores close to 1.0). Highly conserved position. | Homozygous or compound hz with p.R65X or p.C201AfsX77 or p.S241del. | arRP53 | Gong et al., 2015; Zou et al., 2018 | Val146Asp more represented (23.7%)in the Chinese population. |
| c.434G>A | p.G145E | 4 | Dramatically reduced ability to convert all-trans RAL to all-trans ROL (5-18% wt). | Compound heterozygous with M1? |  | Thompson et al., 2005; Valverde et al., 2009 |  |
| c.429_432del4insGGT | p.His143GlnfsX19 | 4 |  |  |  | Thompson et al., 2005 |  |
| c.146 C>T | p.Thr49Met |  | Reduced affinity for NADPH and increased proteosomal degradation. Reduction of RDH activity. Predicted prob damaging (polyphen), deleterius (provean),damaging (SIFT). | Homozygous or compound hz with p.R62X. |  | Janecke et al., 2004; Thompson et al., 2005; Lee et al., 2007; Lee et al., 2010; Fahim et al., 2019. |  |
| c.446T>C | p.L149P |  |  | Compound heterozygous with p.L99I | arRP | Chacon-Camacho et al., 2013 |  |
| c.451C>G | p.His151Asp | 5 | Positively charged his into a not-charged amino acid. Dramatically reduced ability to convert all-trans RAL to all-trans ROL (5-18% wt). Predicted intolerant (SIFT), probably damaging (polyphen2), neutral (pMut), deleterius (provean). | Homozygous or hz with p.A269AfsX1 |  | McKay et al., 2011; Fahim et al., 2019. |  |
|  | p.His151Asn | 5 | Positively charged his into a negatively charged amino acid. |  |  | Perrault et al., 2004 |  |
| c.582C>A | p.Y194X | 5 |  |  |  | Thompson et al., 2005 |  |
| c.464C>T | p.T155I | 5 | Dramatically reduced ability to convert all-trans RAL to all-trans ROL (5-18% wt). Predicted prob damaging (polyphen), deleterius (provean), damaging (SIFT). | Homozygous |  | Thompson et al., 2005; Schuster et al., 2007; Valverde et al., 2009; Fahim et al., 2019. |  |
| c.468C.G | p.Tyr156X | 5 |  | Compound heterozygous with Arg169Trp | EOSRD | Zou et al., 2018 |  |
| c.617C>A | p.A206D | 5 | Dramatically reduced ability to convert all-trans RAL to all-trans ROL (5-18% wt). |  | LCA/EOSRD | Thompson et al., 2005; Carver lab. |  |
| c. 658+1G>A | aberrant splicing | 5 |  | compound hz with p.A269fsX270 | LCA/EOSRD | Perrault et al., 2004 |  |
| c.524C>T | p.S175L | 5 | Predicted intolerant (SIFT), prob damaging (polyphen2), neutral (pMut). | Compound heterozygous with S241del or p.R169W or p.V208E | LCA | Coppieters et al., 2010; McKay et al., 2011; Wang et al., 2015 |  |
| c.523T>C | p.Ser175Pro | 5 |  | Compound hz with p.Tyr226Cys |  | Perrault et al., 2004 |  |
| c.535C>G | p.H179D | 5 | Transversion. Predicted as benign by both SIFT (0.36) and PolyPhen-2 (0.188). | Compound hz with p.T55K | LCA | Li et al., 2017 |  |
| c.565C>T | p.Q189X | 5 | STOP. Protein truncation or NMD. Loss of enzymatic activity. | Homozygous | LCA | Schuster et al., 2007; Janecke et al., 2004 |  |
|  | N125K | 5 | Mild phenotype. <10% normal activity in vitro. Dramatically reduced ability to convert all-trans RAL to all-trans ROL (5-18% wt). | Compound heterozygous with Arg234His |  | Thompson et al., 2005; Valverde et al., 2009; Fahim et al., 2019 |  |
|  | p.S165del5bp | 5 |  | Com hz with A62X (??) |  | Walia et al., 2010. |  |
| c.481C>T | p.R161W | 5 | Exon 5. Predicted tolerant (SIFT), possibly damaging (polyphen2),pathological (pMut), deleterius (provean). | Comp hz with p.V238VfsX34 or p.A269AfsX1 |  | McKay et al., 2011; Fahim et al., 2019. |  |
| c.482G>A | p.R161Q | 5 | Assumed to be a polymorphism in a previous work (Thompson et al., 2005). Far from the catalytic, substrate and co-factor binding sites, does not interfere with catalysis. The enzymatic activity is similar to wt. Predicted tolerant (SIFT),benign (polyphen2), pathological (pMut). | Homozygous or heterozygous | LCA/CORD/RP |  |  |
| c.506G>A | p.R169Q | 5 | Exon 5. Predicted intolerant (SIFT), probably damaging (polyphen2), pathological (pMut), deleterius (provean). | Homozygous or comp hz with p.P20del or Tyr156Ter | EOSRD; LCA | McKay et al., 2011; Zou et al., 2018; Fahim et al., 2019; Carver lab. |  |
| c.454T>A | p.F152I | 5 | Predicted intolerant (SIFT), probably damaging (polyphen2), neutral (pMut), deleterius (provean). | Homozygous |  | McKay et al., 2011; Fahim et al., 2019. |  |
| c.599A>G | p.Y200C | 5 | Predicted intolerant (SIFT), probably damaging (polyphen2), pathological (pMut). | Homozygous |  | McKay et al., 2011 |  |
| c.601 T>C | p.C201R | 5 | Its proximity to and likely disruption of the catalytic site causes altered activity. 201 is inside the catalytic motif (YXXXK), immediately after the Tyr residue. Moreover, it can perturb oligomers formation./Reduced (30%) expression when expressed in Sf9 cells/Catalytically inactive (<10%).The change from a neutral nonpolar residue to a basic polar residue within the catalytic motif can disrupt the proper alignment of residues involved in catalysis. Predicted tolerant (SIFT), possibly damaging (polyphen2),pathological (pMut), deleterius (provean). | Homozygous | LCA | Sun et al., 2007; McKay et al., 2011; Fahim et al., 2019. | The most frequent mutation in pts with Indian descent |
| c.601delT | p.C201AfsX77 | 5 |  | Compound hz with p.V146D |  | Wang et al., 2015 |  |
| c.609C>A | p.S203R | 5 | Predicted intolerant (SIFT), probably damaging (polyphen2),neutral(pMut),deleterius (provean) | Homozygous |  | McKay et al., 2011 |  |
| c.619A>G | p.N207D | 5 | Exon 5. Predicted intolerant (SIFT), probably/poss damaging (polyphen2),neutral(pMut), deleterius (provean). | Homozygous |  | McKay et al., 2011; Fahim et al., 2019; Moosaje. |  |
| c.505C>G | p.R169G | 5 |  | Compound heterozygous with R295X |  | Wang et al., 2015 |  |
| c.505C>T | p.R169W | 5 | Predicted intolerant (SIFT), probably damaging (polyphen2), pathological (pMut). | Comp hz with p.S175L |  | McKay et al., 2011 |  |
| c.623T>A | p.V208E |  | MS | Compound heterozygous with p.S175L | LCA | Coppieters et al., 2010; Wang et al., 2015 |  |
|  | p.Leu214Pro |  | MS | Comp hz with p.lIe22Gly |  | Aleman et al., 2018. |  |
| c.680_684delinsT | p.Ala227Valfs*50 |  | Frameshift. |  |  | Fahim et al., 2019. |  |
| c.701G>A | p.R234H | 6 | Retained 44% the activity level of wildtype. Predicted benign (polyphen), neutral (provean), tolerated (SIFT).I It is likely an hypomorphic allele. Preserved visual funcion also late in adulthood | Compound heterozygous with N125K and with Ala269Glyfs*2 | mild phenotype | Thompson et al., 2005; Valverde et al., 2009; Fahim et al., 2019 |  |
| c.710T.C | p.Leu237Pro | 6 |  | Homozygous | LCA | Zou et al., 2018 |  |
| c.714insC | p.V238VfsX34 | 6 | Null mutation. | Homozygous or Comp hz with p.R161W | LCA | Walia et al., 2010; McKay et al., 2011; Aleman et al; 2018; Carver lab. |  |
| c.715C>G | p.R239W | 6 | Dramatically reduced ability to convert all-trans RAL to all-trans ROL (5-18% wt). Predicted intolerant (SIFT),prob damaging (polyphen2), pathological (pMut), deleterius (provean). | Compound hz with p.A269AfsX1 | LCA/EOSRD | Thompson et al., 2005; McKay et al., 2011; Fahim et al., 2019; Carver lab. |  |
| c.714_715insC | p.Arg239Argfs | 6 | Frameshift. |  |  | Fahim et al., 2019. |  |
| c.721_723delTCC | p.S241del | 6 |  | Compound heterozygous with V146D |  | Wang et al., 2015 |  |
| c.671 C>T | p.Thr224Ile |  | Predicted probably damaging (polyphen), deleterius (provean), damaging (SIFT). |  |  | Fahim et al., 2019. |  |
| 677A>G | p.Y226C | 6 | Diminished enzymatic activity in COS-7 cells. Predicted damaging (Polyphen), deleterious (provean), damaging (SIFT). | Homozygous or compound hz with p.Ser175Pro or p.Ala109Pro | LCA | Schuster et al., 2007; Janecke et al., 2004; Perrault et al., 2004; Fahim et al., 2019; Moosaje (Unpublished) |  |
| c.821T>C | p.L274P | 6 | Dramatically reduced ability to convert all-trans RAL to all-trans ROL (5-18% wt). | Homozygous | LCA/EOSRD | Thompson et al., 2005; Schuster et al., 2007 |  |
| c.687C>G | p.Pro230Ala | 6 |  |  |  |  |  |
| c.700G>C | Val233Leu | 6 | MS mutation in exon 6 predicted to be neutral. Predicted intolerant (SIFT), probably damaging (polyphen2), neutral (pMut), deleterious (provean). | Compound heterozygous with Ala269del or Ser134Pro |  | McKay et al., 2011; Aleman et al., 2018; Fahim et al., 2019. | third most frequent in Aleman et al., 2018 (24%) |
| c.698T>A | Val233Glu | 6 | MS mutation. Val233 is highly conserved. | Homozygous |  | Sodi et al., 2010. |  |
| c.698insGT | p.V233VfsX45 | 6 | Frameshift | Heterozygous |  | McKay et al., 2011; Fahim et al., 2019. |  |
| c.698 T>A | p.Val233Asp | 6 | Predicted probably damaging (polyphen), deleterious (provean), damaging (SIFT). | Comp hz with del 4 bp TCCA codon 19 |  | Walia et al., 2010; Fahim et al., 2019. |  |
| c.776delG |  |  | FS mutation and a premature termination at codon 277. It does not directly alter the dehydrogenase functional domain; however, it alters or eliminates the terminal 57 aas of RDH12, which are highly conserved. It alters RDH12 structure. | Heterozygous | autosomal dominant RP53 (adRP) | Fingert et al., 2008 |  |
| c.738_746delGCTCTGGCG | p.Leu247_Arg249del |  |  | Comp hz with p.Gly145Arg | arRP | Zou et al., 2018 |  |
| c.759del | p.Phe254Leufs*24 | 6 | Abnormal protein that is toxic to the photoreceptor. | Heterozygous | autosomal dominant RP53 | Moosaje; Rosin et al. 2018 (abstract) |  |
| c.776delG | p.Arg259 del1cG | 6 |  |  | LCA | Carver lab |  |
| c.780delG | p.Glu260 del1gaG | 6 |  |  | LCA | Carver lab |  |
| c.806_810delCCCTG | p.A269fsX270 | 6 | 5 bps-deletion at codon 269 in exon 6. Defective enzymatic activity (0 in COS-7 cells) .Loss of the residues needed to form a seventh β-strand next to canonical sixth β-strand in the Rossmann fold. Defective enzymatic activity. Null mutation. | Homozygous or Compound hz with L99I or p.Pro230Ala or p.T49M or p.R161W or Val233Leu or Arg295Stop or p.Ala126Glu or Thr45Ala | LCA/EOSRD | Janecke et al., 2004; Thompson et al., 2005; Sun et al., 2007; Valverde et al., 2009; Aleman et al., 2018; Fahim et al., 2019 | The most frequent mutations in pts with British Caucasian descendant (Fahim et al., 2019) |
| c.448+1 G>A | aberrant splicing |  |  |  |  | McKay et al., 2011; Fahim et al., 2019. |  |
| c.883C>T | p.R295X | 7 | Null mutation at the tail end (last 21 aa) of the protein. It likely leads to NMD. | Homozygous or Compound heterozygous with R169G or Ala269del or Asp101Gly or T55M. | LCA/EOSRD | Thompson et al., 2005; Walia et al., 2010; Wang et al., 2015; Aleman et al., 2018; Fahim et al., 2019; Carver lab. | Second most frequent mutation in Aleman et al., 2018 (33%) |
|  | p.R193C |  | retained 79% the activity level of wildtype. May not disease causing. | Homozygous |  | Thompson et al., 2005 |  |
|  | p.A269GfsX1 |  |  | Homozygous | LCA | Schuster et al., 2007 |  |
| c.854G>A | p.C285Y | 7 | Dramatically reduced ability to convert all-trans RAL to all-trans ROL (5-18% wt). | Homozygous | LCA/EOSRD | Thompson et al., 2005; Walia et al., 2010 |  |
| c.910 T>C | p.Trp304Arg | 7 | Predicted probably damaging (polyphen), deleterius (provean), damaging (SIFT). |  |  | Fahim et al., 2019. |  |
| c.912G>A | p.Trp304Stop | 7 | Trp304 is highly conserved across different species. It is a nonsense mutation causing a premature STOP. | Homozygous |  | Sodi et al., 2010. |  |
| c.IVS2 as-1G.A | p.splice defect |  |  | Comp hz with p.Val146Asp |  | Zou et al., 2018 |  |
| c.343+1G.A | p.splice defect |  |  | Comp hz with c.343+1G.A (splice defect) | arRP | Zou et al., 2018 |  |

**APPENDIX B**

RDH12 BIBLIOGRAPHY

March 2019

- Aleman TS, Uyhazi KE, Serrano LW, Vasireddy V, Bowman SJ, Ammar MJ, Pearson DJ, Maguire AM, Bennett J. RDH12 mutations cause a severe retinal degeneration with relatively spared rod function. Invest Ophthalmol Vis Sci. 2018 Oct 1;59(12):5225-5236.

- Barnea-Cramer AO, Wang W, Lu SJ, Singh MS, Luo C, Huo H, McClements ME, Barnard AR, MacLaren RE, Lanza R. Function of human pluripotent stem cell-derived photoreceptor progenitors in blind mice. Sci Rep. 2016 Jul 13;6:29784.

- Belyaeva OV, Korkina OV, Stetsenko AV, Kim T, Nelson PS, Kedishvili NY. Biochemical properties of purified human retinol dehydrogenase 12 (RDH12): Catalytic efficiency toward retinoids and C9 aldehydes and effects of cellular retinol-binding protein type I (CRBPI) and cellular retinaldehyde binding protein (CRALBP) on the oxidation and reduction of retinoids. Biochemistry 2005;44(18): 7035–7047.

- Belyaeva OV, Lee SA, Kolupaev OV, Kedishvili NY. Identification and characterization of retinoid-active short-chain dehydrogenases/reductases in Drosophila melanogaster. Biochim Biophys Acta. 2009 Oct;1790(10):1266-73.

- Bhalla S, Joshi D, Bhullar S, Kasuga D, Park Y, Kay CN, 2013. Long-term follow-up for efficacy and safety of treatment of retinitis pigmentosa with valproic acid. Br J Ophthalmol 97, 895–899.

- Birch DG, Bennett LD, Duncan JL, Weleber RG, Pennesi ME, 2016. Long-term follow-up of patients with retinitis pigmentosa receiving intraocular ciliary neurotrophic factor implants. Am. J. Ophthalmol. 170, 10–14.

- Boatright JH, Nickerson JM, Moring AG, Pardue MT. Bile acids in treatment of ocular disease. J Ocul Biol Dis Infor. 2009 Sep;2(3):149-159.

- Byrne LC, Dalkara D, Luna G, Fisher SK, Clerin E, Sahel JA, Leveillard T, Flannery JG, 2015. Viral-mediated RdCVF and RdCVFL expression protects cone and rod photoreceptors in retinal degeneration. J. Clin. Invest 125, 105–116.

- Campochiaro PA, Strauss RW, Lu L, et al. Is there excess oxidative stress and damage in eyes of patients with retinitis pigmentosa? Antioxid Redox Signal. 2015;23:643–648.

- Chen C, Thompson DA, Koutalos Y. Reduction of All-trans-retinal in vertebrate rod photoreceptors requires the combined action of RDH8 and RDH12. J Biol Chem. 2012 Jul 13;287(29):24662-70.

- Chen FK, McLenachan S, Edel M, Da Cruz L, Coffey PJ, Mackey DA. iPS Cells for Modelling and Treatment of Retinal Diseases. J Clin Med. 2014 Dec 19;3(4):1511-41.

- Chen Y, Palczewska G, Masuho I, Gao S, Jin H, Dong Z, Gieser L, Brooks MJ, Kiser PD, Kern TS, Martemyanov KA, Swaroop A, Palczewski K. Synergistically acting agonists and antagonists of G protein-coupled receptors prevent photoreceptor cell degeneration. Sci Signal. 2016 Jul 26;9(438):ra74.

- Chrispell JD, Feathers KL, Kane MA, Kim CY, Brooks M, Khanna R, Kurth I, Hübner CA, Gal A, Mears AJ, Swaroop A, Napoli JL, Sparrow JR, Thompson DA. Rdh12 activity and effects on retinoid processing in the murine retina. J Biol Chem. 2009 Aug 7;284(32):21468-77.

- Cideciyan AV, Swider M, Aleman TS, et al. ABCA4-associated retinal degenerations spare structure and function of the human parapapillary retina. Invest Ophthalmol Vis Sci 2005;46:4739–46.

- Cideciyan AV, Jacobson SG, Beltran WA, et al. Human retinal gene therapy for Leber congenital amaurosis shows advancing retinal degeneration despite enduring visual improvement. Proc Natl Acad Sci U S A. 2013;110: E517-E525.

- Cideciyan AV and Jacobson SG. Leber Congenital Amaurosis (LCA): potential for improvement of vision. Invest Ophthalmol Vis Sci. 2019 Apr 1;60(5):1680-1695.

- Cideciyan AV, Jacobson SG, Drack AV, et al. Effect of an intravitreal antisense oligonucleotide on vision in Leber congenital amaurosis due to a photoreceptor cilium defect. Nat Med. 2019;25:225–228.

- da Cruz L, Fynes K, Georgiadis O, et al. Phase 1 clinical study of an embryonic stem cell-derived retinal pigment epithelium patch in age-related macular degeneration Nature Biotechnology, vol. 36, no. 4, pp. 328–337, 2018.

- D'Aniello E, Ravisankar P, Waxman JS. Rdh10a Provides a Conserved Critical Step in the Synthesis of Retinoic Acid during Zebrafish Embryogenesis. PLoS One. 2015 Sep 22;10(9):e0138588.

- Day, T.P., Byrne, L.C., Schaffer, D.V., Flannery, J.G., 2014. Advances in AAV vector development for gene therapy in the retina. Adv. Exp. Med. Biol. 801, 687–693.

- Dias MF, Joo K, Kemp JA, Fialho SL, da Silva Cunha A Jr, Woo SJ, Kwon YJ. Molecular genetics and emerging therapies for retinitis pigmentosa: Basic research and clinical perspectives. Prog Retin Eye Res. 2018 Mar; 63:107-131.

- Doonan F, O'Driscoll C, Kenna P, Cotter TG, 2011. Enhancing survival of photoreceptor cells in vivo using the synthetic progestin Norgestrel. J. Neurochem. 118, 915–927.

- Duncan JL, Pearce EA, et al. Inherited Retinal Degenerations: current landscape and knowledge gaps. Transl Vis Sci Technol. 2018 Jul 18;7(4):6.

- Elachouri G, Lee-Rivera I, Clérin E, Argentini M, Fridlich R, Blond F, Ferracane V, Yang Y, Raffelsberger W, Wan J, Bennett J, Sahel J-A, Zack DJ, Léveillard T. Thioredoxin rod-derived cone viability factor protects against photooxidative retinal damage. Free Radic. Biol. Med. 81, 22–29 (2015).

- Fahim AT, Bouzia Z, Branham KH, Kumaran N, Vargas ME, Feathers KL, Perera ND, Young K, Khan NW, Heckenlively JR, Webster AR, Pennesi ME, Ali RR, Thompson DA, Michaelides M. Detailed clinical characterisation, unique features and natural history of autosomal recessive RDH12-associated retinal degeneration. Br J Ophthalmol. 2019 Apr 12.

- Feathers KL, Jia L, Perera ND, Chen A, Presswalla FK, Khan NW, Fahim AT, Smith AJ, Ali RR, Thompson DA. Development of a gene-therapy vector for *RDH12*-associated retinal dystrophy. Hum Gene Ther. 2019 Jun 25. doi: 10.1089/hum.2019.017.

- Fingert JH. Association of a novel mutation in the retinol dehydrogenase 12 (RDH12) gene with autosomal dominant retinitis pigmentosa. Arch Ophthalmol. 2008;126:1301–1307.

- Garg A, Lee W, Sengillo JD, Allikmets R, Garg K, Tsang SH. Peripapillary sparing in RDH12-associated Leber congenital amaurosis. Ophthalmic Genet. 2017 December; 38(6): 575–579.

- Ghazi NG, Abboud EB, Nowilaty SR, et al. Treatment of retinitis pigmentosa due to MERTK mutations by ocular subretinal injection of adenoassociated virus gene vector: results of a phase I trial. Hum Genet. 2016; 135:327-343.

- Goldmann T, Overlack N, Moller F, Belakhov V, vanWyk M, Baasov T, Wolfrum U and Nagel-Wolfrum K. (2012) A comparative evaluation of NB30, NB54 and PTC124 in translational read-through efficacy for treatment of an USH1C nonsense mutation. EMBO Mol. Med., 4, 1186–1199.

- Gorbatyuk MS, Knox T, LaVail MM, Gorbatyuk OS, Noorwez SM, Hauswirth

WW, Lin JH, Muzyczka N, Lewin AS, 2010. Restoration of visual function in P23H rhodopsin transgenic rats by gene delivery of BiP/Grp78. Proc Natl Acad Sci USA. 107, 5961–5966.

- Haeseleer F, [Jang GF](https://www.ncbi.nlm.nih.gov/pubmed/?term=Jang%2520GF%255BAuthor%255D&cauthor=true&cauthor_uid=12226107), [Imanishi Y](https://www.ncbi.nlm.nih.gov/pubmed/?term=Imanishi%2520Y%255BAuthor%255D&cauthor=true&cauthor_uid=12226107), [Driessen CAGG](https://www.ncbi.nlm.nih.gov/pubmed/?term=Driessen%2520CAGG%255BAuthor%255D&cauthor=true&cauthor_uid=12226107), [Matsumura M](https://www.ncbi.nlm.nih.gov/pubmed/?term=Matsumura%2520M%255BAuthor%255D&cauthor=true&cauthor_uid=12226107), [Nelson PS](https://www.ncbi.nlm.nih.gov/pubmed/?term=Nelson%2520PS%255BAuthor%255D&cauthor=true&cauthor_uid=12226107), Palczewski K. Dual-substrate specificity short chain retinol dehydrogenases from the vertebrate retina. [J Biol Chem.](https://www.ncbi.nlm.nih.gov/pubmed/?term=rdh12%252C+haseleer) 2002 Nov 22;277(47):45537-45546.

- Hai T et al. Creation of miniature pig model of human Waardenburg syndrome type 2A by ENU mutagenesis. Hum Genet. 2017 Nov;136(11-12):1463-1475.

- Hofmann L, Tsybovsky Y, Alexander NS, Babino D, Leung NY, Montell C, Banerjee S, von Lintig J, Palczewski K. Structural Insights into the Drosophila melanogaster Retinol Dehydrogenase, a Member of the Short-Chain Dehydrogenase/Reductase Family. Biochemistry. 2016 Nov 29; 55(47):6545-6557.

- Human Gene Mutation Database (HGMD®). <http://www.hgmd.cf.ac.uk>

- Iraha S, Hirami Y, Ota S, Sunagawa GA, Mandai M, Tanihara H, Takahashi M, Kurimoto Y. 2016. Efficacy of valproic acid for retinitis pigmentosa patients: a pilot study. Clin Ophthalmol 10, 1375–1384.

- Jacobson SG, Aleman TS, Cideciyan AV, et al. Identifying photoreceptors in blind eyes caused by RPE65 mutations: prerequisite for human gene therapy success. Proc Natl Acad Sci U S A. 2005;102:6177–6182.

- Jacobson SG, Cideciyan AV, Aleman TS, et al. RDH12 and RPE65, visual cycle genes causing Leber congenital amaurosis, differ in disease expression. Invest Ophthalmol Vis Sci. 2007; 48:332–338.

- Jacobson SG, Cideciyan AV, Ratnakaram R, et al. Gene therapy for Leber congenital amaurosis caused by RPE65 mutations: safety and efficacy in 15 children and adults followed up to 3 years. Arch Ophthalmol. 2012;130: 9-24.

- Janecke AR, Thompson DA, Utermann G, Becker C, Hèubner CA, Schmid E, McHenry CL, Nair AR, Rèuschendorf F, Heckenlively J, Wissinger B, Nèurnberg P, Gal A. Mutations in RDH12 encoding a photoreceptor cell retinol dehydrogenase cause childhood-onset severe retinal dystrophy. Nat Genet 2004;36:850–854.

- Jornvall H, Persson B, Krook M, Atrian S, Gonzalez-Duarte R, Jeffery J, et al. Short-chain dehydrogenases/reductases (SDR). Biochemistry 1995;34(18):6003–6013.

- Kashani AH, Lebkowski JS, Rahhal FM et al. A bioengineered retinal pigment epithelial monolayer for advanced, dry age-related macular degeneration. Science Translational Medicine, vol. 10, no. 435, 2018.

- Koenekoop RK. An overview of Leber congenital amaurosis: a model to understand human retinal development. Surv Ophthalmol 2004;49:379–98.

- Koenekoop RK, Sui R, Sallum J, van den Born LI, Ajlan R, Khan A, den Hollander AI, Cremers FP, Mendola JD, Bittner AK, Dagnelie G, Schuchard RA, Saperstein DA. Oral 9-cis retinoid for childhood blindness due to Leber congenital amaurosis caused by RPE65 or LRAT mutations: an open-label phase 1b trial. Lancet. 2014 Oct 25;384(9953):1513-20.

- Kumar A, Midha N, Gogia V, Gupta S, Sehra S, Chohan A. 2014. Efficacy of oral valproic acid in patients with retinitis pigmentosa. J Ocul Pharmacol Ther 30, 580–586.

- Kumaran N, Leber congenital amaurosis/early-onset severe retinal dystrophy: clinical features, molecular genetics and therapeutic interventions. Br J Ophthalmol 2017;101:1147–1154.

- Kuniyoshi K, Sakuramoto H, Yoshitake K, et al. Longitudinal clinical course of three Japanese patients with Leber congenital amaurosis/early-onset retinal dystrophy with RDH12 mutation. Doc Ophthalmol. 2014;128:219–228.

- Kuriyan AE, Albini TA, Townsend JH, Rodriguez M, Pandya HK, Leonard 2nd,

RE, Parrott MB, Rosenfeld PJ, Flynn Jr H, Goldberg JL. 2017. Vision loss after intravitreal injection of autologous "stem cells" for AMD. N. Engl. J. Med. 376, 1047–1053.

- Kurth I, Thompson DA, Ruther K, Feathers KL, Chrispell JD, Schroth J, et al. Targeted disruption of the murine retinal dehydrogenase gene Rdh12 does not limit visual cycle function. Molecular and Cellular Biology 2007;27(4):1370–1379.

- Lamba DA, Gust J, and Reh TA. Transplantation of human embryonic stem cell-derived photoreceptors restores some visual function in Crx-deficient mice. Cell Stem Cell, vol. 4, no. 1, pp. 73–79, 2009.

- Lee SA, Belyaeva OV, Kedishvili NY. Disease-associated variants of microsomal retinol dehydrogenase 12 (RDH12) are degraded at mutant-specific rates. FEBS Letters 584 (2010) 507–510.

- Lee SY, Usui S, Zafar AB, Oveson BC, Jo YJ, Lu L, Masoudi S, Campochiaro PA, 2011 N-acetylcysteine promotes long-term survival of cones in a model of retinitis pigmentosa. J. Cell Physiol. 226, 1843–1849.

- Leonard KC, Petrin D, Coupland SG, Baker AN, Leonard BC, LaCasse EC, Hauswirth WW, Korneluk RG, Tsilfidis C, 2007. XIAP protection of photoreceptors in animal models of retinitis pigmentosa. PLoS One 2, e314.

- Liang FQ, Aleman TS, Dejneka NS, Dudus L, Fisher KJ, Maguire AM, Jacobson SG, Bennett J, 2001. Long-term protection of retinal structure but not function using RAAV.CNTF in animal models of retinitis pigmentosa. Mol. Ther. 4, 461–472.

- Lotery AJ, Yang GS, Mullins RF, et al. Adeno‐associated virus type 5: transduction efficiency and cell-type specificity in the primate retina. Hum Gene Ther 2003;14:1663-1671.

- Lukovic D, Artero Castro A, León M, Del Buey Furió V, Cortón M, Ayuso C, Erceg S. Generation of a human iPSC line from a patient with Leber congenital amaurosis caused by mutation in AIPL1. Stem Cell Res. 2018 Dec;33:151-155.

- Mackay DS, Dev Borman A, Moradi P, et al. RDH12 retinopathy: novel mutations and phenotypic description. Mol Vis. 2011;17:2706–2716.

- Maeda A, Maeda T, Imanishi Y, Sun W, Jastrzebska B, Hatala DA, et al. Retinol dehydrogenase (RDH12) protects photoreceptors from light-induced degeneration in mice. Journal of Biological Chemistry 2006;281(49):37697–37704.

- Maeda A, Maeda T, Sun W, Zhang H, Baehr W, Palczewski K. Redundant and unique roles of retinol dehydrogenases in the mouse retina. Proc Natl Acad Sci U S A. 2007 Dec 4;104(49):19565-70.

- Maeda A, Mandai M, Takahashi M. Gene and induced pluripotent stem cell therapy for retinal diseases. Annu Rev Genomics Hum Genet. 2019 Apr 24.

- Maeder ML, Stefanidakis M, Wilson CJ, Baral R et al. Development of a gene-editing approach to restore vision loss in Leber congenital amaurosis type 10. Nat Med. 2019 Feb;25(2):229-233.

- Mandai M, Watanabe A, Kurimoto Y et al. Autologous induced stem-cell-derived retinal cells for macular degeneration. The New England Journal of Medicine, vol. 376, no. 11, pp. 1038–1046, 2017.

- M’Barek KB and Monville C. Cell Therapy for Retinal Dystrophies: From cell suspension formulation to complex retinal tissue bioengineering. Stem Cells Int. 2019 Jan 23;2019:4568979.

- Marchette LD, Thompson DA, Kravtsova M, Ngansop TN, Mandal MN, Kasus-Jacobi A. Retinol dehydrogenase 12 detoxifies 4-hydroxynonenal in photoreceptor cells. Free Radic Biol Med 2010 January 1; 48(1): 16–25.

- Marchette LD, Wang H, Li F, Babizhayev MA, Kasus-Jacobi A. Carcinine has 4-hydroxynonenal scavenging property and neuroprotective effect in mouse retina. Invest Ophthalmol Vis Sci. 2012 Jun 20;53(7):3572-83.

- Megaw R, Abu-Arafeh H, Jungnickel M, Mellough C, Gurniak C, Witke W, Zhang W, Khanna H, Mill P, Dhillon B, Wright AF, Lako M, French-Constant C. Gelsolin dysfunction causes photoreceptor loss in induced pluripotent cell and animal retinitis pigmentosa models. Nat Commun. 2017 Aug 16;8(1):271.

- Nadauld LD, Chidester S, Shelton DN, Rai K, Broadbent T, Sandoval IT, Peterson PW, Manos EJ, Ireland CM, Yost HJ, Jones DA. Dual roles for adenomatous polyposis coli in regulating retinoic acid biosynthesis and Wnt during ocular development. Proc Natl Acad Sci U S A. 2006 Sep 5;103(36):13409-14.

- Nakazawa M, Ohguro H, Takeuchi K, Miyagawa Y, Ito T, Metoki T, 2011. Effect of nilvadipine on central visual field in retinitis pigmentosa: a 30-month clinical trial. Ophthalmologica 225, 120–126.

- Nakazawa M, Suzuki Y, Ito T, Metoki T, Kudo T, Ohguro H, 2013. Long-term effects of nilvadipine against progression of the central visual field defect in retinitis pigmentosa: an extended study. Biomed. Res. Int. 2013, 585729.

- Orban T, Leinonen H, Getter T, Dong Z, Sun W, Gao S, Veenstra A, Heidari-Torkabadi H, Kern TS, Kiser PD, Palczewski K. A combination of G protein-coupled receptor modulators protects photoreceptors from degeneration. Pharmacol Exp Ther. 2018 Feb;364(2):207-220.

- Parfitt DA, Lane A, Ramsden CM, Carr AJ, Munro PM, Jovanovic K, Schwarz N, Kanuga N, Muthiah MN, Hull S, Gallo JM, da Cruz L, Moore AT, Hardcastle AJ, Coffey PJ, Cheetham ME. Identification and correction of mechanisms underlying inherited blindness in human iPSC-derived optic cups. Cell Stem Cell. 2016 Jun 2;18(6):769-81.

- Pawlyk BS, Li T, Scimeca MS, Sandberg MA, Berson EL, 2002. Absence of photoreceptor rescue with D-cis-diltiazem in the rd mouse. Invest Ophthalmol. Vis Sci 43, 1912–1915.

- Pearce-Kelling SE, Aleman TS, Nickle A, Laties AM, Aguirre GD, Jacobson SG, Acland GM, 2001. Calcium channel blocker D-cis-diltiazem does not slow retinal degeneration in the PDE6B mutant rcd1 canine model of retinitis pigmentosa. Mol Vis 7, 42–47.

- Phillips MJ, Walker TA, Choi HY, Faulkner AE, Kim MK, Sidney SS, Boyd AP, Nickerson JM, Boatright JH, Pardue MT. Tauroursodeoxycholic acid preservation of photoreceptor structure and function in the rd10 mouse through postnatal day 30. Invest Ophthalmol Vis Sci. 2008 May;49(5):2148-55.

- Pierce EA, Bennett J. The status of RPE65 gene therapy trials: safety and efficacy. Cold Spring Harb Perspect ed. 2015; 5:a017285.

- Platania CBM, Dei Cas M, Cianciolo S, Fidilio A, Lazzara F, Paroni R, Pignatello R, Strettoi E, Ghidoni R, Drago F, Bucolo C. Novel ophthalmic formulation of myriocin: implications in retinitis pigmentosa. Drug Deliv. 2019 Dec;26(1):237-243.

- Ramachandran PS, Lee V, Wei Z, Song JY, Casal G, Cronin T, et al. Evaluation of dose and safety of AAV7m8 and AAV8BP2 in the non-human primate retina. Hum Gene Ther. 2017;28(2):154–67.

- Ramsden CM, Nommiste B, R Lane A, Carr AF, Powner MB, JK Smart M, Chen LL, Muthiah MN, Webster AR, Moore AT, Cheetham ME, da Cruz L, Coffey PJ. Rescue of the MERTK phagocytic defect in a human iPSC disease model using translational read-through inducing drugs. Sci Rep. 2017 Mar 3;7(1):51.

- Russell S, Bennett J, Wellman JA, et al. Efficacy and safety of voretigene neparvovec (AAV2-hRPE65v2) in patients with RPE65-mediated inherited retinal dystrophy: a randomised, controlled, open-label, phase 3 trial. Lancet. 2017;390:849–860.

- Sahel JA, Dalkara D. Gene therapy for retinal dystrophy. Nat Med. 2019 Feb;25(2):198-199.

- Sandberg MA, Rosner B, Weigel-DiFranco C, Berson, EL, 2011. Lack of scientific rationale for use of valproic acid for retinitis pigmentosa. Br J Ophthalmol 95, 744.

- Schimel AM, Abraham L, Cox D, et al. N-acetylcysteine amide (NACA) prevents retinal degeneration by up-regulating reduced glutathione production and reversing lipid peroxidation. Am J Pathol. 2011;178:2032–2043.

- Scholl HPN et al. RET IRD 01 Study Group, Safety and proof-of-concept study of oral

QLT091001 in retinitis pigmentosa due to inherited deficiencies of retinal pigment epithelial 65 protein (RPE65) or lecithin: retinol acyltransferase (LRAT). PLOS ONE 10, e0143846 (2015).

- Scholl HP, Strauss RW, Singh MS, Dalkara D, Roska B, Picaud S, Sahel JA. Emerging therapies for inherited retinal degeneration. Sci Transl Med. 2016 Dec 7;8(368):368rv6.

- Schuster A, Janecke AR, Wilke R, et al. The phenotype of early-onset retinal degeneration in persons with RDH12 mutations. Invest Ophthalmol Vis Sci. 2007;48:1824–1831.

- Schwarz N, Carr AJ, Lane A, Moeller F, Chen LL, Aguilà M, Nommiste B, Muthiah MN, Kanuga N, Wolfrum U, Nagel-Wolfrum K, da Cruz L, Coffey PJ, Cheetham ME, Hardcastle AJ. Translational read-through of the RP2 Arg120stop mutation in patient iPSC-derived retinal pigment epithelium cells. Human Molecular Genetics, 2015, Vol. 24, No. 4 972–986.

- Schwartz SD, Hubschman JP, Heilwell G et al. Embryonic stem cell trials for macular degeneration: a preliminary report. The Lancet, vol. 379, no. 9817, pp. 713–720, 2012.

- Schwartz SD, Regillo CD, Lam BL et al. Human embryonic stem cell-derived retinal pigment epithelium in patients with age-related macular degeneration and Stargardt’s macular dystrophy: follow-up of two open-label phase 1/2 studies. The Lancet, vol. 385, no. 9967, pp. 509–516, 2015.

- Shanmugam PM, Minija CK, Ramanjulu R, Tekwani P, Saxena M, 2012. Effect of short-term oral valproic Acid on vision and visual field in retinitis pigmentosa. Ophthalmol Ther 1, 6.

- Shirai H, Mandai M, Matsushita K, et al. Transplantation of human embryonic stem cell-derived retinal tissue in two primate models of retinal degeneration. Proceedings of the National Academy of Sciences, vol. 113, no. 1, pp. E81–E90, 2016.

- Simunovic MP, Shen W, Lin JY, Protti DA, Lisowski L, Gillies MC. Optogenetic approaches to vision restoration. Exp Eye Res. 2019 Jan;178:15-26.

- Singh R, Shen W, Kuai D, Martin JM, Guo X, Smith MA, Perez ET, Phillips MJ, Simonett JM, Wallace KA, Verhoeven AD, Capowski EE, Zhang X, Yin Y, Halbach PJ, Fishman GA, Wright LS, Pattnaik BR, Gamm DM. iPS cell modeling of Best disease: insights into the pathophysiology of an inherited macular degeneration. Hum Mol Genet. 2013 Feb 1;22(3):593-607.

- Song JY, Aravand P, Nikonov S, Leo L, Lyubarsky A, Bennicelli JL, Pan J, Wei Z, Shpylchak I, Herrera P, Bennett DJ, Commins N, Maguire AM, Pham J, den Hollander AI, Cremers FPM, Koenekoop RK, Roepman R, Nishina P, Zhou S, Pan W, Ying GS, Aleman TS, de Melo J, McNamara I, Sun J, Mills J, Bennett J. Amelioration of neurosensory structure and function in animal and cellular models of a congenital blindness. Mol Ther. 2018 Jun 6;26(6):1581-1593.

- Song WK, Park KM, Kim HJ et al. Treatment of macular degeneration using embryonic stem cell-derived retinal pigment epithelium: preliminary results in Asian patients. Stem Cell Reports, vol. 4, no. 5, pp. 860–872, 2015.

- Stone EM. Leber congenital amaurosis - a model for efficient genetic testing of

heterogeneous disorders: lxiv Edward Jackson Memorial Lecture. Am J Ophthalmol

2007;144:791–811.

- Strettoi E, Gargini C, Novelli E, Sala G, Piano I, Gasco P, Ghidoni R. Inhibition of ceramide biosynthesis preserves photoreceptor structure and function in a mouse model of retinitis pigmentosa. Proc Natl Acad Sci U S A. 2010 Oct 26;107(43):18706-11.

- Sun W, Novel RDH12 mutations associated with Leber congenital amaurosis and cone-rod dystrophy: Biochemical and clinical evaluations. [Vision Res.](https://www.ncbi.nlm.nih.gov/pubmed/?term=Novel+RDH12+mutations+associated+with+Leber+congenital+amaurosis+and+cone-rod+dystrophy%253A+Biochemical+and+clinical+evaluations) 2007 Jul;47(15):2055-66.

- Testa F, Maguire AM, Rossi S, et al. Three-year follow-up after unilateral subretinal delivery of adeno-associated virus in patients with Leber congenital amaurosis type 2. Ophthalmology. 2013; 120:1283-1291.

- Thompson DA, Janecke AR, Lange J, Feathers KL, Hubner CA, McHenry CL, Stockton DW, Rammesmayer G, Lupski JR, Antinolo G, Ayuso C, Baiget M, Gouras P, Heckenlively JR, den Hollander A, Jacobson SG, Lewis RA, Sieving PA, Wissinger B, Yzer S, Zrenner E, Utermann G, Gal A. Retinal degeneration associated with RDH12 mutations results from decreased 11-cis retinal synthesis due to disruption of the visual cycle. Hum Mol Genet 2005;14:3865–3875.

- Travis GH, Golczak M, Moise AR, Palczewski K. Diseases caused by defects in the visual cycle: retinoids as potential therapeutic agents. Annu Rev Pharmacol Toxicol. 2007 ; 47: 469–512.

- Tucker BA, Mullins RF, Streb LM, Anfinson K, Eyestone ME, Kaalberg E, Riker MJ, Drack AV, Braun TA, Stone EM. Patient-specific iPSC-derived photoreceptor precursor cells as a means to investigate retinitis pigmentosa. Elife. 2013 Aug 27;2:e00824.

- Tucker BA, Cranston CM, Anfinson KA, Shrestha S, Streb LM, Leon A, Mullins RF, Stone EM. Using patient-specific induced pluripotent stem cells to interrogate the pathogenicity of a novel retinal pigment epithelium-specific 65 kDa cryptic splice site mutation and confirm eligibility for enrollment into a clinical gene augmentation trial. Transl Res. 2015 Dec;166(6):740-749.e1.

- Vancura P, Csicsely E, Leiser A, Iuvone PM, Spessert R. Rhythmic regulation of photoreceptor and RPE genes important for vision and genetically associated with severe retinal diseases. [Invest Ophthalmol Vis Sci.](https://www.ncbi.nlm.nih.gov/pubmed/?term=vancura%252C+rdh12) 2018 Aug 1;59(10):3789-3799.

- Vandenberghe LH, Bell P, Maguire AM, et al. Dosage thresholds for AAV2 and AAV8 photoreceptor gene therapy in monkey. Sci Transl Med. 2011; 3:88ra54.

- Vang S, Longley K, Steer CJ, Low WC. The unexpected uses of urso- and tauroursodeoxycholic acid in the treatment of non-liver diseases. Glob Adv Health Med. 2014 May;3(3):58-69.

- Van Hooser JP, Aleman TS, He Y-G, Cideciyan AV, Kuksa V, Pittler SJ, Stone EM, Jacobson SG, Palczewski K. Rapid restoration of visual pigment and function with oral retinoid in a mouse model of childhood blindness. Proc Natl Acad Sci USA 97, 8623–8628 (2000).

- Van Hooser JP, Liang Y, Maeda T, Kuksa V, Jang GF, He YG, Rieke F, Fong HK, Detwiler PB, Palczewski K. Recovery of visual functions in a mouse model of Leber congenital amaurosis. J. Biol. Chem. 277, 19173–19182 (2002).

- Wang X, Wang T, Jiao Y, von Lintig J, Montell C. Requirement for an enzymatic visual cycle in Drosophila. Curr Biol. 2010 Jan 26;20(2):93-102.

- Ward R, Sundaramurthi H, Di Giacomo V, Kennedy BN. Enhancing Understanding of the Visual Cycle by Applying CRISPR/Cas9 Gene Editing in Zebrafish. Front Cell Dev Biol. 2018 Apr 11;6:37.

- Yang GS, Schmidt M, Yan Z, et al. Virus‐mediated transduction of murine retina with

adeno‐associated virus: effects of viral capsid and genome size. J Virol 2002;76:7651-7660.

- Zhang H, Huang J, Li Z, Qin G, Zhang N, Hai T, Hong Q, Zheng Q, Zhang Y, Song R, Yao J, Cao C, Zhao J, Zhou Q. Rescuing ocular development in an anophthalmic pig by blastocyst complementation. EMBO Mol Med. 2018 Dec;10(12).

- Zhang X, Zhang D, Chen SC, Lamey T, Thompson JA, McLaren T, De Roach JN, Chen FK, McLenachan S. Establishment of an induced pluripotent stem cell line from a retinitis pigmentosa patient with compound heterozygous CRB1 mutation. Stem Cell Res. 2018 Aug;31:147-151.

- Zhong X, Gutierrez C, Xue T, Hampton C, Vergara MN, Cao LH, Peters A, Park TS, Zambidis ET, Meyer JS, Gamm DM, Yau KW, Canto-Soler MV. Generation of three-dimensional retinal tissue with functional photoreceptors from human iPSCs. Nat Commun. 2014 Jun 10;5:4047.

- Zou X, Fu Q, Fang S, Li H, Ge Z, Yang L, Xu M, Sun Z, Li H, Li Y, Dong F, Chen R, Sui R. Phenotypic variability of recessive RDH12-associated retinal dystrophy.  Retina. 2018 Aug 21.

**APPENDIX C**

Bibliographic analysis performed through Scopus and PubMed in March 2019

A first PubMed search for RDH12 meant to identifying the major players in the field and the prominent areas of investigations, returned the following results:

Articles’ types

86 papers (any type) on RDH12 were published. 70 were classified as Journal articles, 9 Case Reports, 6 Review articles, 0 Clinical Trial and 1 Observational Study.

Animals’ species:

Animals’ species found in the retrieved papers were human (28), mouse (18), fly (3), rat (2), pig (2), fish (1), dog (1) and non-human primates (1).

A second search was performed through Scopus. Aims were: 1) to depict the “geography” of RDH12-associated LCA and LCA; 2) to identify the most relevant areas of active investigation.

First query had “RDH12” as keyword to search titles, abstracts, papers’ keywords.

**Results**: **98** papers published from 2002 (when *RDH12* gene was cloned) (in Appendix C, the complete list of these papers is provided) with the following highlights:

**Years of publication:**


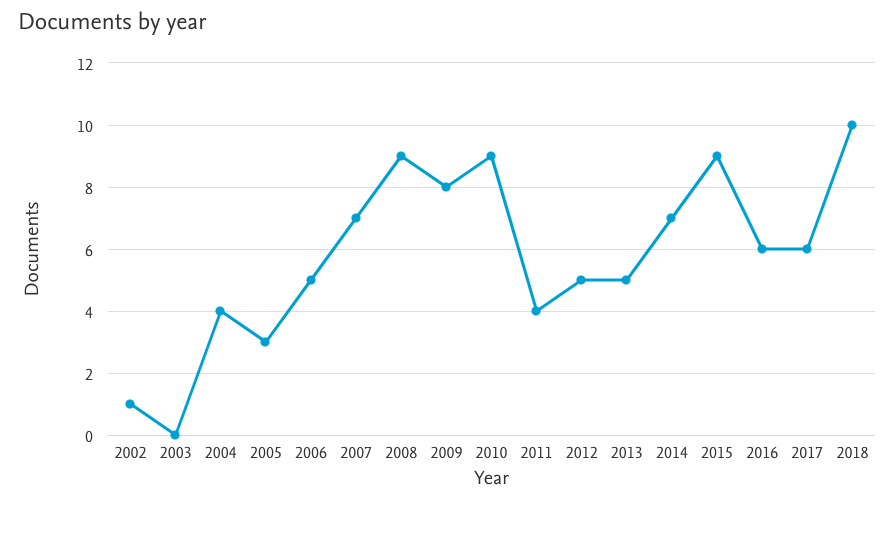


**Authors:**


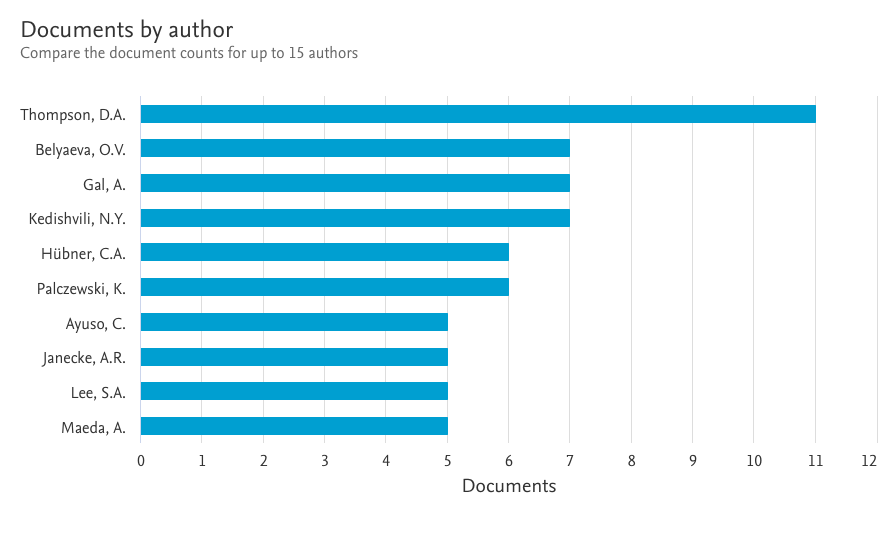


**Affiliations:**


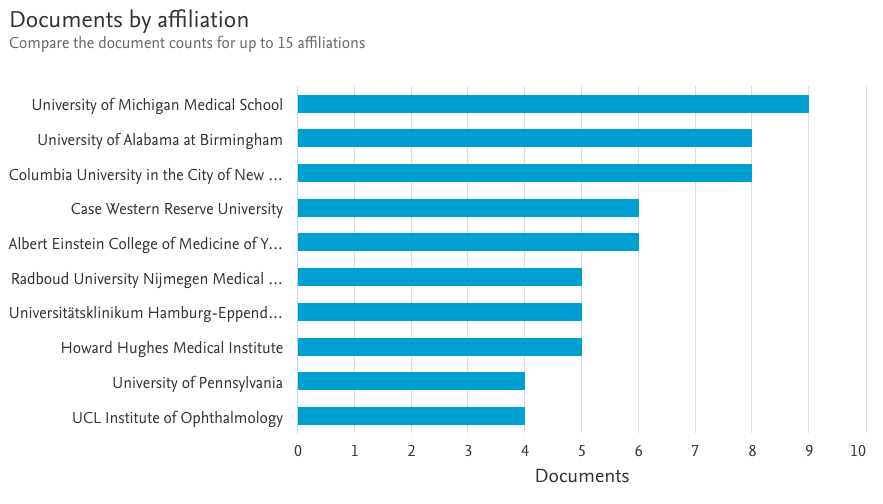


**Countries:**


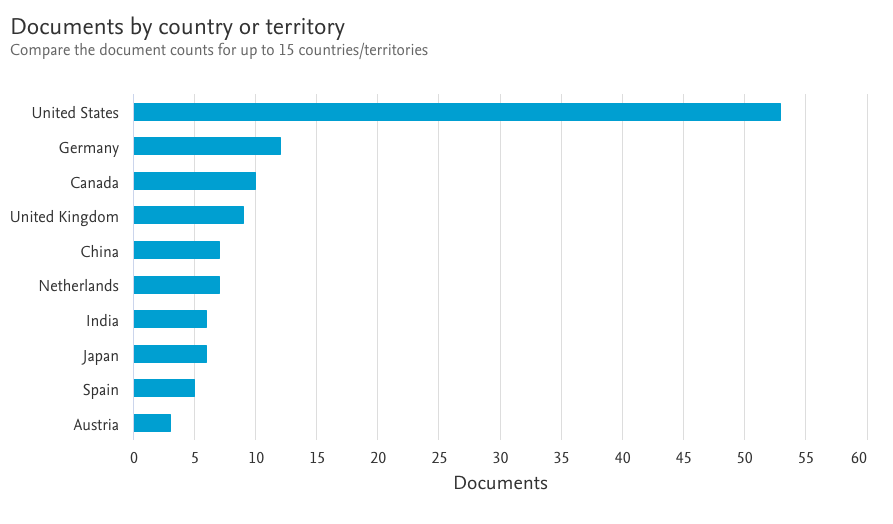


**Subject area:**


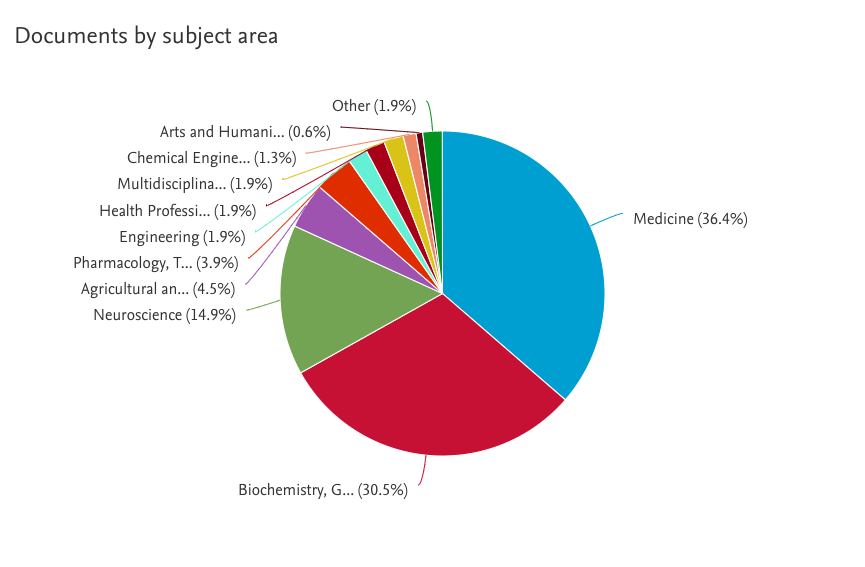


A second query addressed Leber congenital amaurosis as a whole and returned: **1,757** papers**.**

**Years of publication:**


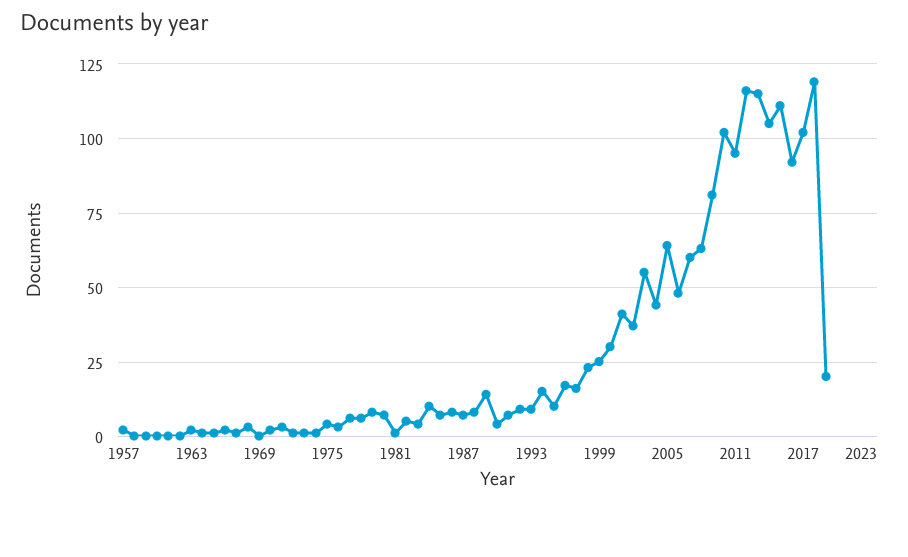


**Authors:**


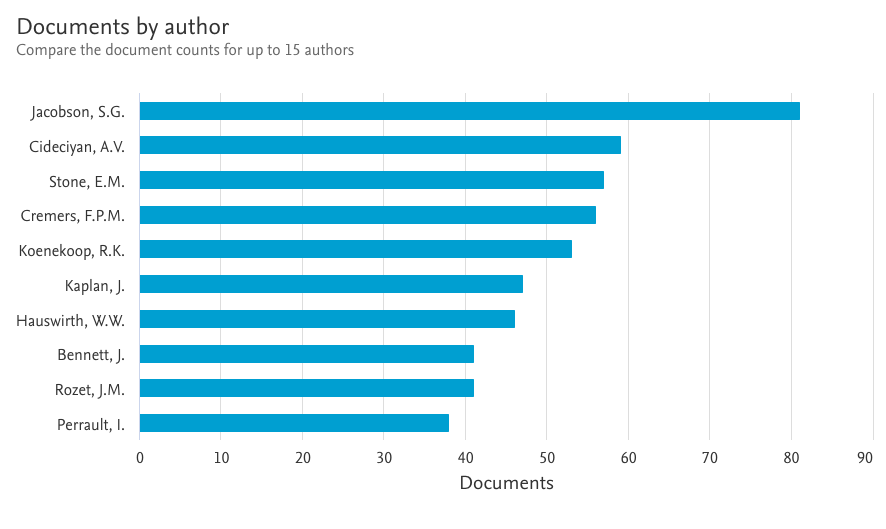


**Affiliations:**


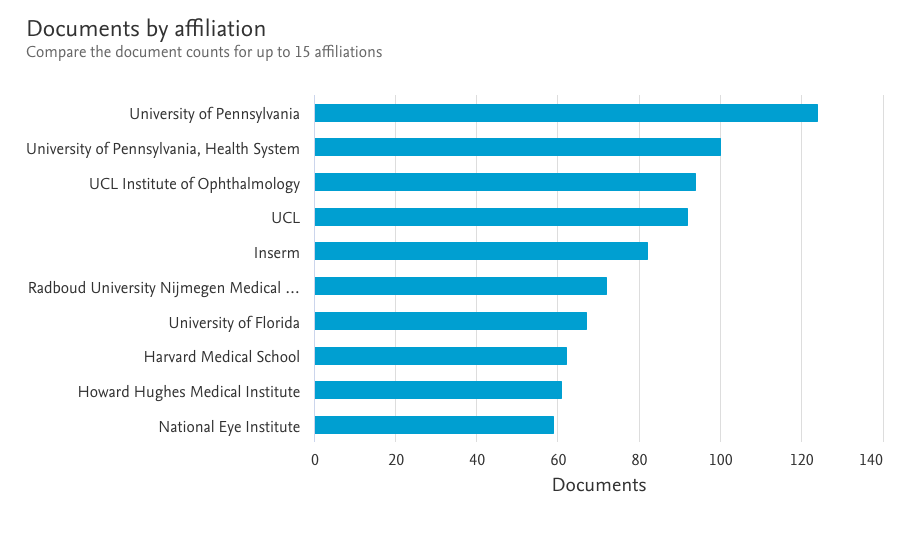


**Countries:**


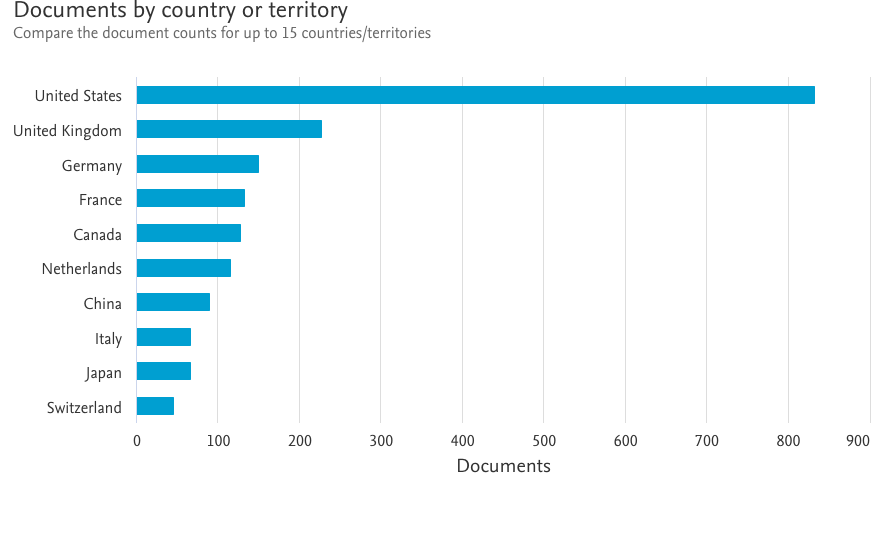


**Subject area:**


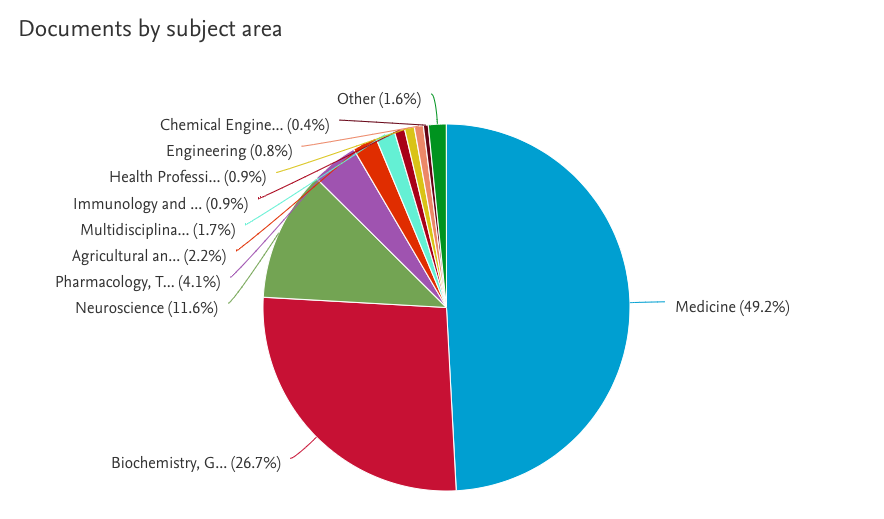


A final search regarded the Italian experts in the field of retinal diseases and resulted in a list of 12 people whose names, affiliation and expertise are reported in table below.

| **Name** | **Affiliation** | **Scientific Interest (keywords)** |
| --- | --- | --- |
| **Monica Gori**  [monica.gori@iit.it](mailto:monica.gori@iit.it)  <https://www.iit.it/it/research/lines/unit-for-visually-impaired-people> | Unit of Visually Impaired People, IIT, Genova | Audio-motor training; multisensory integration; rehabilitation; spatial cognition in visually impaired children |
| **Valeria Marigo**  [valeria*.*marigo@unimore.it](mailto:valeria.marigo@unimore.it) | Università Modena e Reggio Emilia | Photoreceptor degeneration; molecular mechanism of retinitis pigmentosa (RP); eye genetic diseases; cGMP targeting |
| **Francesca Fanelli**  [francesca.fanelli@unimore.it](mailto:francesca.fanelli@unimore.it)  <http://www.csbl.unimore.it/proff-francesca-fanelli/> | Università Modena e Reggio Emilia | RP; computational biology; structural modeling; G protein-Coupled Receptors (GPCRs) |
| **Leonardo Colombo**  [leonardo.colombo.82@gmail.com](mailto:leonardo.colombo.82@gmail.com) | Retinal Dystrophies Unit, San Paolo Hospital, Milan and UNIMI | Argus II artificial retina implant; vitreo-retinal surgery; retinal dystrophies; RP; clinical research.  Collaboration with Sahel (Paris) |
| **Riccardo Ghidoni**  [riccardo.ghidoni@unimi.it](mailto:riccardo.ghidoni@unimi.it) | San Paolo Hospital, Milan and UNIMI | Inherited retinal degenerations; biochemistry;  serine palmitoyltransferase inhibitors |
| **Enrica Strettoi**  [enrica.strettoi@in.cnr.it](mailto:enrica.strettoi@in.cnr.it) | Istituto di Neuroscienze, CNR, Pisa | Retinal neurobiology; photoreceptor degeneration; retinal remodeling; RP; environmental enrichment; pharmacology |
| **Alberto Auricchio**  [auricchio@tigem.it](mailto:auricchio@tigem.it)  <http://www.tigem.it/research/faculty/auricchio> | Tigem (NA) | Gene therapy; adeno-associated virus (AAV); Leber Congenital Amaurosis (LCA); Stargardt macular degeneration |
| **Francesca Simonelli**  [Francesca.simonelli@unina2.it](mailto:Francesca.simonelli@unina2.it) | UOC Oculistica, Seconda Universita’ di Napoli | Clinical research; ophthalmology; retinal dystrophies; LCA; RP; Stargardt; gene therapy |
| **Andrea Sodi**  [a.sodi@tin.it](mailto:a.sodi@tin.it) | Azienda Ospedaliero Universitaria Careggi (FI) | Genetics; ophthalmology; inherited retinal dystrophies; LCA |
| **Giacomo M. Bacci**  [g.bacci@meyer.it](mailto:g.bacci@meyer.it) | Pediatric  Ophthalmology Unit, Azienda Ospedaliero Universitaria Meyer | Genetics; ophtalmology |
| **Paolo E. Bianchi**  [pe.bianchi@smatteo.pv.it](mailto:pe.bianchi@smatteo.pv.it)  [pe.bianchi@unipv.it](mailto:pe.bianchi@unipv.it) | Clinica Oculistica, Universita’ di Pavia, Policlinico San Matteo (PV) | Ophthalmology, Surgery, Neuro-ophthalmology; glaucoma; LCA; clinical research  Nel direttivo di IALCA onlus |
| **Elisa Fazzi**  [efazzi@unipv.it](mailto:efazzi@unipv.it)  or [elisa.fazzi@unibs.it](mailto:elisa.fazzi@unibs.it) | Neuroftalmologia dell'Età Evolutiva, IRCCS Mondino Institute of Neurology (PV) or Neuropsichiatria infantile, Spedali Civili (BS) | LCA; clinical research; Neuro-ophtalmology; Pediatric; Infantile Neuropsychiatry  Direttore scientifico di IALCA onlus (Associazione Italiana Amaurosi Congenita di Leber) |

1. Science Compass is an Italy-based consulting firm that enables charities, research organizations and patient associations to outsource scientific office tasks. Services provided range from scientific assessment/monitoring in any given field of biomedicine (but mainly in the rare disease field), strategic planning and management of research-funding activities including design of calls for proposals, peer review and research portfolio administration. For more information, visit: [www.sciencecompass.it](http://www.sciencecompass.it) [↑](#footnote-ref-2)
2. Scopus (<https://www.scopus.com/home.uri>) is the largest abstract and citation database of peer-reviewed literature. It features a handful of tools to track, analyze and visualize research. [↑](#footnote-ref-3)
3. Protocols for generation of iPS cells require that a skin biopsy is collected by a non-invasive and safe procedure. Then, patient-specific fibroblasts are derived, maintained in culture, and stored in appropriate cell banks. These cells can be reprogrammed to iPSCs through a mixture of key factors. The time lag from biopsy to validation of the best iPSC clone may take 2-3 months. [↑](#footnote-ref-4)
4. Mutations of patient derived-fibroblasts and blood cells are the following:

   - c.759del, p.Phe254Leufs*24: autosomal dominant heterozygous;

   - c.619A>G, p.Asx207Asp: autosomal recessive homozygous;

   - c.325G>A and c.677A>G, p.Ala109Pro and p.Tyr226Cys: autosomal recessive, compound heterozygote. [↑](#footnote-ref-5)
5. <https://www.myretinatracker.org> [↑](#footnote-ref-6)
